# NEK2A regulates PLIN2 expression through a SERBP1 dependent pathway

**DOI:** 10.1016/j.isci.2026.116254

**Published:** 2026-06-09

**Authors:** Tomohiko Makiyama, Toshihiro Aiuchi, Tomoko Mikajiri, Takashi Obama, Masahiro Chatani, Yuki Azetsu, Atsushi Yamada, Kiyohito Sasa, Hiroyuki Itabe

**Affiliations:** 1 Department of Biological Chemistry, Showa Medical University Graduate School of Pharmacy, 1-5-8 Hatanodai, Shinagawa, Tokyo 142-8555, Japan; 2 Department of Pharmacology, Graduate School of Dentistry, Showa Medical University, 1-5-8 Hatanodai, Shinagawa, Tokyo 142-8555, Japan; 3 Pharmacological Research Center, Showa Medical University, 1-5-8 Hatanodai, Shinagawa, Tokyo 142-8555, Japan; 4 Department of Biochemistry, Graduate School of Dentistry, Showa Medical University, 1-5-8 Hatanodai, Shinagawa, Tokyo 142-8555, Japan; 5 Department of Oral Physiology, Graduate School of Dentistry, Showa Medical University, 1-5-8 Hatanodai, Shinagawa, Tokyo 142-8555, Japan

**Keywords:** health sciences, biological sciences, biochemistry, molecular biology, metabolomics

## Abstract

Lipid droplets are key organelles in lipid metabolism, and expression of the lipid droplet protein PLIN2 is tightly regulated. Here, we identify serine/threonine kinase NEK2A as a negative regulator of PLIN2 expression in human hepatoma cells. NEK2A depletion increased PLIN2 protein and mRNA levels, whereas its overexpression suppressed both. NEK2A contains two domains, a kinase domain and a coiled-coil domain. Notably, both the kinase-dead mutant and the truncated kinase domain suppressed PLIN2 expression, indicating that this regulation depends on the kinase domain but not its catalytic activity. Proteomic analysis identified SERBP1 as a NEK2A-binding partner required for this regulation. SERBP1 depletion abrogated NEK2A-mediated PLIN2 suppression, and the increase in PLIN2 expression was reversed by a PPARγ inhibitor. These findings define a kinase-independent NEK2A-SERBP1 pathway that regulates PLIN2 expression without altering triacylglycerol levels and provide insight into lipid droplet protein regulation beyond lipid storage.

## Highlights

• NEK2A negatively regulates PLIN2 expression in hepatoma cells
• PLIN2 expression depends on the NEK2A kinase domain but not its kinase activity
• SERBP1 is required for NEK2A-mediated PLIN2 suppression
• The NEK2A-SERBP1 axis modulates the PPARγ-dependent transcription of PLIN2

## Graphical abstract

**Figure:**
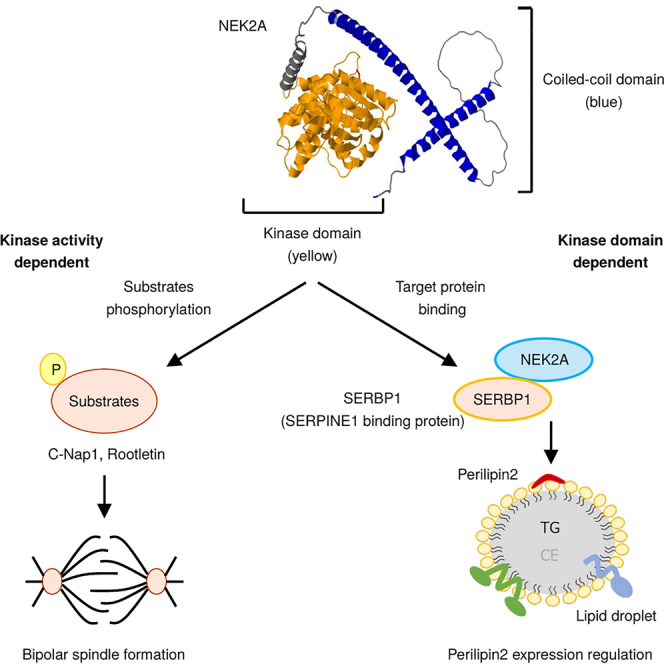

## Introduction

Lipid droplets (LDs) are ubiquitous intracellular organelles found in many cell types that store triacylglycerol (TG) and cholesteryl ester.^1^^,^^2^ Recent studies have shown that LDs accumulate in liver cells in metabolic dysfunction-associated fatty liver disease (MAFLD; formerly called NAFLD) model mice^3^ and in hepatocellular carcinoma (HCC).^4^ LD protein composition varies depending on the cell type,^5^^,^^6^ and many studies have suggested that LD formation is a regulated process that has not been fully studied.^7^^,^^8^^,^^9^^,^^10^^,^^11^ The perilipin (PLIN) family proteins, PLIN1–PLIN5, are well-known LD-associated proteins that possess conserved domains.^12^^,^^13^ PLIN2 is ubiquitously expressed and is a major LD protein in the liver.^13^ PLIN3, which is also widely expressed in many types of cells,^13^^,^^14^ can transfer between organelles such as LD and lysosomes, and it is thought to compensate for PLIN2 to maintain LD stability when PLIN2 on the LD surface decreases.^15^ Indeed, in *Plin2*-deficient models, increased the expression of PLIN3 has been observed, consistent with compensatory remodeling of LD-coating proteins.^16^^,^^17^^,^^18^ PLIN2 and PLIN3 represent the predominant LD proteins in hepatocytes and play central roles in maintaining LD homeostasis. Both PLIN2 and PLIN3 are widely used as LD marker proteins,^13^^,^^14^ thus understanding their intracellular distribution and protein expression levels is necessary to elucidate LD homeostasis.

Other PLIN family members show distinct tissue-specific functions. PLIN1 is predominantly expressed in adipocytes and plays a critical role in LD stabilization and regulation of lipolysis.^19^ PLIN4 (also known as S3-12) is enriched in adipose tissue and contributes to LD coating^20^; however, its function in lipid metabolism is not well defined, as *Plin4* knockout mice do not exhibit a significant phenotype in body weight or adipose tissue mass.^21^ PLIN5 is highly expressed in oxidative tissues such as heart and skeletal muscle, where it regulates lipid metabolism and promotes LD-mitochondria interactions.^22^ In contrast, PLIN2 and PLIN3 are the major LD proteins in the liver.

Early studies showed that PLIN2 is transcriptionally induced when TG accumulates in cells, and PLIN2 is reduced by the ubiquitin-proteasome pathway upon the regression of LDs.^23^^,^^24^ However, recent studies have highlighted the role of PLIN2 in cellular processes beyond lipid storage, including autophagy, metabolism, and cell proliferation, suggesting that its regulation may not be solely dependent on the intracellular lipid content.^25^^,^^26^ We previously reported that PLIN2 protein levels decreased during mitosis, even though LDs remained throughout the mitotic processes.^27^ This finding suggests that PLIN2 may be regulated by another process beyond lipid storage. In another study, we found a serine/threonine kinase NEK2A, a regulator of spindle formation, dynamically increased during mitosis,^28^ raising the possibility that NEK2A might contribute to PLIN2 regulation. These observations prompted the exploration of upstream regulators of PLIN2, particularly in the context of dynamic cellular events such as mitosis and cell cycle progression.

NIMA-related kinase (NEK) family members are serine/threonine kinases that have been shown to play important roles in cell-cycle progression.^29^ The mammalian NEK family consists of eleven members (NEK1–NEK11), and each of them contains a conserved N-terminal serine/threonine kinase domain and variable C-terminal regulatory regions that confer substrate specificity and subcellular localization.^30^ Among the NEK family proteins, NEK2A is localized in both the nucleus and cytoplasm and exhibits a cell-cycle-dependent expression pattern.^31^^,^^32^^,^^33^ It is low in the G1 phase, increases during the G1/S transition, and is degraded via the anaphase-promoting complex/cyclosome (APC/C) pathway at the onset of mitosis.^28^^,^^34^^,^^35^ NEK2A phosphorylates intracentriolar linker proteins, including rootletin and centrosomal protein 250 (CEP250, also known as C-Nap1), thereby promoting centrosome separation and bipolar spindle assembly.^29^^,^^31^ In addition to cell cycle regulation, NEK2A is also involved in anti-cancer drug resistance,^36^ autophagosome formation,^37^ and cancer progression.^38^ Interestingly, although all NEK family members harbor a kinase domain, several—such as NEK6 and NEK9—have been reported to exert functions that depend on their kinase domain structure rather than catalytic activity. NEK6 participates in spindle formation and apoptosis through scaffold-like interactions, while NEK9 regulates microtubule organization and centrosome dynamics via its N-terminal domain independent of enzymatic activity.^30^^,^^39^ These precedents raise the possibility that NEK2A, which is best known for its kinase-dependent role in spindle regulation, might also exert a non-canonical function in modulating PLIN2 expression. This supports the hypothesis that NEK2A-mediated PLIN2 regulation extends across the cell cycle, rather than being restricted to mitosis. In parallel, increasing attention has been paid to the transcriptional regulation of LD-associated proteins and their importance in metabolic diseases and cellular homeostasis.^40^^,^^41^^,^^42^ However, no studies have examined the role of NEK2A in PLIN2 regulation.

We investigated whether NEK2A regulates PLIN2 expression. By combining knockdown, overexpression, and domain-mapping approaches, we demonstrated that NEK2A negatively modulates PLIN2 expression in a manner independent of its kinase activity. Furthermore, we identified SERBP1 as an NEK2A-associated factor required for this regulation. SERBP1 is an RNA-binding protein known to participate in transcriptional and post-transcriptional regulation, and our findings suggest that the NEK2A-SERBP1 axis contributes to LD protein homeostasis through a kinase-independent transcriptional mechanism.

## Results

### NEK2A-dependent PLIN2 expression in cells

Recently, we showed that PLIN2 protein levels decrease during mitosis and that intracellular LDs are aligned outside the bipolar spindle.^27^ Since the expression of NEK2A, a regulator of spindle formation, increased during mitosis,^28^^,^^30^^,^^34^ we have investigated whether it also regulates PLIN2 protein expression during mitosis.

**Figure 1:**
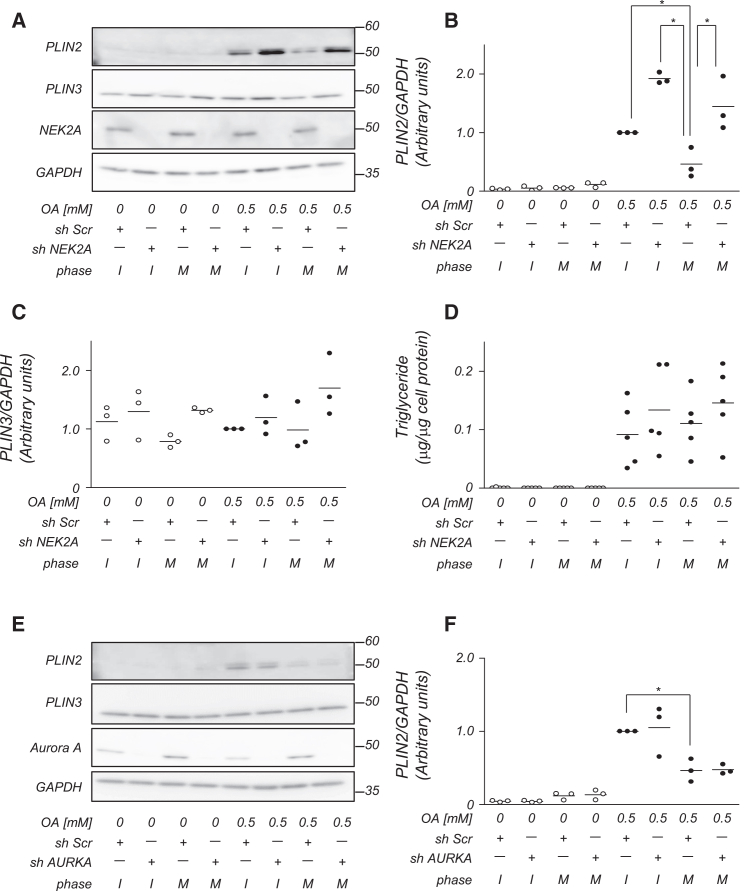
NEK2A-dependent PLIN2 expression in cells HuH7 cells were treated with 9 μM RO-3306, a selective cyclin-dependent kinase 1 inhibitor, for 20 h to synchronize cells at the G2/M boundary. HuH7 cells were treated with scrambled short hairpin RNA (sh Scr) or shRNA targeting NEK2A (sh *NEK2A*) and incubated with or without 0.5 mM oleic acid (OA). (A) Cell lysates were analyzed by immunoblotting with antibodies against PLIN2, PLIN3, NEK2A, and glyceraldehyde 3-phosphate dehydrogenase (GAPDH). Data shown are representative of three independent experiments. (B and C) Relative protein levels of PLIN2 and PLIN3 were quantified, normalized to those of GAPDH, and expressed in arbitrary units. Protein levels in OA-treated sh Scr cells during interphase were set to 1.0. The results are shown as scattered dot plots (mean ± SD). (D) Intracellular triglyceride (TG) levels were quantified and presented as scattered dot plots (mean ± SD). (E) HuH7 cells treated with sh Scr or shRNA against Aurora A (sh *AURKA*) were incubated with or without 0.5 mM OA. Cell lysates were analyzed by immunoblotting using antibodies against PLIN2, PLIN3, Aurora A, and GAPDH. Data shown are representative of three independent experiments. (F) Relative protein levels of PLIN2 were quantified, normalized to those of GAPDH, and expressed in arbitrary units. PLIN2 levels in OA-treated sh Scr cells during interphase were set to 1.0. The results are shown as scattered dot plots (mean ± SD). I: interphase; M: mitotic phase. ∗*p* < 0.05, one-way ANOVA followed by Tukey-Kramer post hoc test.

NEK2A knockdown using short hairpin RNA (shRNA) effectively reduced NEK2A protein levels compared with cells treated with scrambled control shRNA (sh Scr) (Figure 1A). Intracellular PLIN2 levels increased after oleic acid (OA) treatment, whereas PLIN2 levels decreased in the mitotic phase compared to those in interphase, as shown previously.^27^ Interestingly, in sh NEK2A-treated cells, PLIN2 levels were significantly higher than those in sh Scr-treated cells (Figures 1A and 1B). In contrast, PLIN3 protein levels remained unchanged in both sh NEK2A-treated and sh Scr-treated cells (Figures 1A and 1C). Furthermore, the TG levels did not differ significantly under these conditions (Figure 1D).

Since Aurora A kinase is also involved in bipolar spindle formation,^43^^,^^44^ we investigated whether Aurora A kinase is involved in PLIN2 protein expression. Aurora A protein levels were reduced in cells treated with shRNA targeting Aurora A kinase (sh AURKA) when compared to those treated with sh Scr (Figure 1E). In shAURKA-treated cells, PLIN2 levels did not change compared to those in sh Scr-treated cells in either interphase or mitotic phase (Figures 1E and 1F).

**Figure 2:**
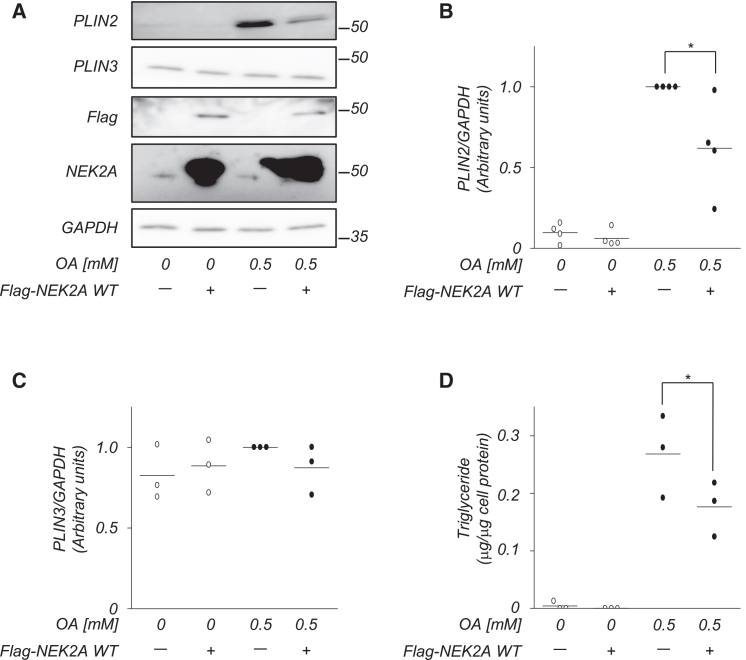
PLIN2 expression suppression by NEK2A overexpression in interphase HuH7 cells overexpressing either mock or Flag-NEK2A WT were incubated with or without 0.5 mM oleic acid (OA). (A) Cell lysates were analyzed by immunoblotting with antibodies against PLIN2, PLIN3, Flag (DYKDDDDK), NEK2A, and glyceraldehyde-3-phosphate dehydrogenase (GAPDH). Data shown are representative of three independent experiments. (B and C) Relative protein levels of PLIN2 and PLIN3 were quantified, normalized to those of GAPDH, and expressed in arbitrary units. The protein levels in OA-treated cells were set to 1.0. The results are shown as scattered dot plots (mean ± SD). (D) Intracellular triglyceride (TG) levels were quantified and presented as scattered dot plots (mean ± SD). ∗*p* < 0.05, one-way ANOVA followed by Tukey-Kramer post hoc test.

When Flag tag-fused NEK2A wild-type (Flag-NEK2A WT) was overexpressed in cells, the PLIN2 levels increased after OA treatment, as expected, but they were notably reduced when compared to those in control cells (Figures 2A and 2B). The PLIN3 levels remained unchanged under all conditions (Figures 2A and 2C). Intracellular TG levels increased after OA treatment but decreased in cells overexpressing Flag-NEK2A WT (Figure 2D).

These findings indicate that NEK2A negatively regulates PLIN2 protein expression independent of the cell cycle phase.

### Behavior of the LD protein in flag-NEK2A WT overexpressing cells

**Figure 3:**
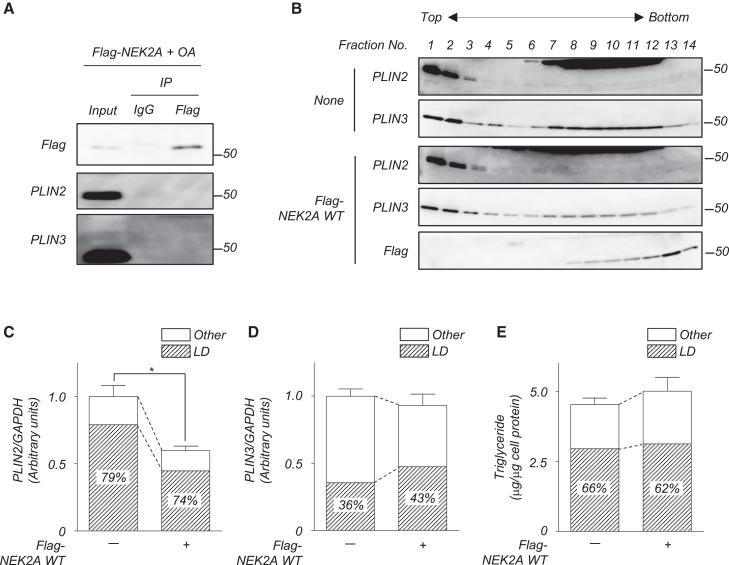
Behavior of the LD protein in Flag-NEK2A WT-overexpressing cells (A) HuH7 cells overexpressing either Flag-NEK2A WT were incubated with or without 0.5 mM oleic acid (OA). Cell lysates were subjected to immunoprecipitation with control IgG or anti-Flag antibodies. The immunoprecipitants were analyzed by immunoblotting with antibodies against Flag (DYKDDDDK), PLIN2, and PLIN3. IP, immunoprecipitation. (B) Lysates from mock- or Flag-NEK2A WT-overexpressing cells containing LDs were fractionated by sucrose density gradient centrifugation. An aliquot of each fraction was analyzed by immunoblotting using antibodies against PLIN2, PLIN3, and Flag. We have previously validated our subcellular fractionation by density gradient ultracentrifugation under the same experimental conditions, using organelle-specific markers. Markers for mitochondria (Bcl-2), Golgi apparatus (GM130), lysosome (LAMP1), and ER (calnexin) were recovered in fractions 12–14. Markers for endosome (EEA1), ER-Golgi transport vesicle (Sec22b), and soluble protein (GFP) were recovered mainly in fractions 8–12 (previously published in our paper^46^). (C and D) The ratio of PLIN2 (C) or PLIN3 (D) band intensity in the LD fractions (lanes 1 and 2, hatched bars) or the non-LD fractions (lanes 3–14, open bars) relative to the total across all of the lanes was calculated. Total intensity was calculated using data from Figures 2B and 2C. (E) Intracellular TG levels were quantified and presented as scattered dot plots (mean ± SD). The ratio of TG levels in LD fractions (lanes 1 and 2, hatched bars) or non-LD fractions (lanes 3–14, open bars) relative to the total fractions was calculated. Total TG levels were determined using data from Figure 2D. ∗*p* < 0.05, one-way ANOVA followed by Tukey-Kramer post hoc test.

Since NEK2A is a Ser/Thr kinase involved in the phosphorylation and stabilization of components required for bipolar spindle formation,^45^ we investigated whether PLIN2 physically associates with NEK2A in cells. Flag-NEK2A WT was immunoprecipitated with an anti-Flag antibody, but not with non-immune IgG; however, no interaction was detected between Flag-NEK2A WT and either PLIN2 or PLIN3 (Figure 3A).

LD-free PLIN2 is reportedly degraded via the ubiquitin-proteasome pathway.^23^^,^^24^ Thus, we have examined the distribution pattern of PLIN2 in cells overexpressing Flag-NEK2A WT using density gradient fractionation. A cell lysate adjusted to 26% sucrose was placed in the middle of a stepwise 2–51% sucrose gradient. After centrifugation, the entire sample was divided into 14 fractions by sequentially collecting equal-volume aliquots from the top. As shown previously,^27^^,^^46^^,^^47^ PLIN2 was mainly found in the top fractions (1 and 2), which were defined as the “LD fraction.” Flag-NEK2A WT was detected in fractions 8–14, indicating that NEK2A is not a component of LDs in cells. Although the ratio of PLIN2 localized in the LD fraction relative to the total did not change upon Flag-NEK2A WT overexpression, the total PLIN2 content in the lysate was found to be reduced to approximately 60% of that in control cells (Figures 3B and 3C). In contrast to PLIN2, the proportion of PLIN3 in the LD fraction increased from 36% to 43% in Flag-NEK2A WT-overexpressing cells (Figures 3B and 3D). This reciprocal change suggests that PLIN3 may compensate for reduced PLIN2 on the LD surface, thereby maintaining overall LD stability. The TG levels in the LD fractions did not change significantly between the two cell types (Figure 3E).

### Transcriptional regulation of PLIN2 in a NEK2A-dependent manner

As mentioned above, LD-free PLIN2 is degraded via the ubiquitin-proteasome pathway.^23^^,^^24^ LDs are also degraded through an autophagy pathway known as lipophagy.^48^^,^^49^^,^^50^^,^^51^ Therefore, we examined whether NEK2A promotes PLIN2 degradation via either of these pathways.

NEK2A, which is degraded via the ubiquitin-proteasome pathway,^28^^,^^34^^,^^35^ was stabilized in the cells treated with MG132 (a proteasome inhibitor) (Figure S1A). Chloroquine, an autophagy inhibitor, leads to the accumulation of LC3B in cells. Under these conditions, neither MG132 nor chloroquine restored the PLIN2 levels in Flag-NEK2A WT-overexpressing cells (Figures S1A and S1B). Both inhibitors caused a slight increase in PLIN2 levels regardless of NEK2A overexpression, indicating partial inhibition of basal degradation pathways. PLIN3 levels remained unchanged under all conditions studied (Figures S1A and S1C). These results indicate that NEK2A does not affect PLIN2 degradation via either the proteasomal or autophagic pathways, although intracellular PLIN2 levels were decreased by the overexpression of Flag-NEK2A WT, as shown in Figure 2B.

Because NEK2A is involved in cell cycle progression, we investigated whether NEK2A overexpression or knockdown induced G2/M cell-cycle arrest. In Figures S2A and S2B, neither NEK2A overexpression nor NEK2A depletion caused cell-cycle arrest at the G2/M phase, suggesting that NEK2A regulates PLIN2 expression without affecting cell cycle progression.

**Figure 4:**
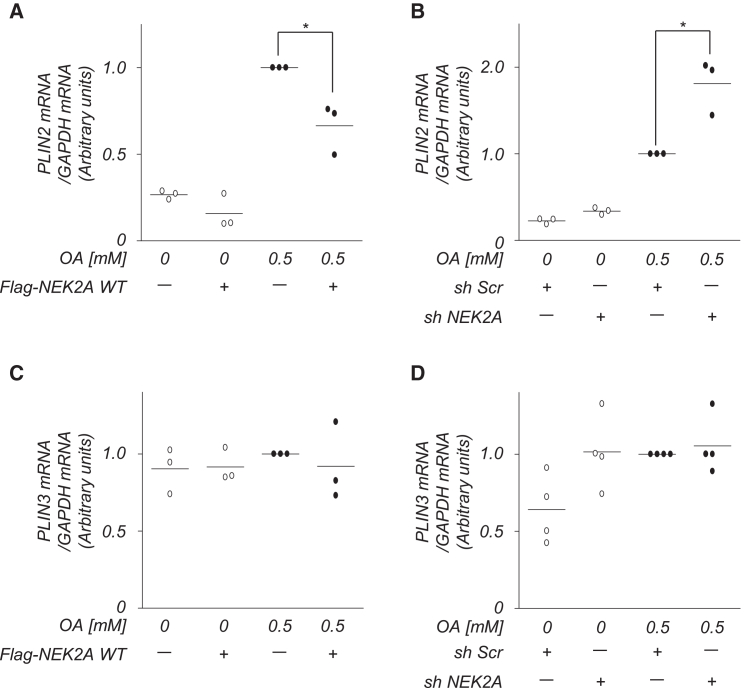
Transcriptional regulation of PLIN2 in a NEK2A-dependent manner HuH7 cells overexpressing Flag-NEK2A WT (A and C) or treated with shRNA targeting *NEK2A* (B and D) were incubated with or without 0.5 mM oleic acid (OA). Total RNA was isolated, and RT-PCR was performed to determine the mRNA levels of *PLIN2* (A and B) and *PLIN3* (C and D), normalized to *GAPDH*. The expression levels in OA-treated cells were set to 1.0. The results are shown as scattered dot plots (mean ± SD). ∗*p* < 0.05, one-way ANOVA followed by Tukey-Kramer post hoc test.

Overexpression of NEK2A in cells decreased the *PLIN2* mRNA levels (Figure 4A), and, in sh NEK2A-treated cells, conversely, the *PLIN2* mRNA levels increased (Figure 4B). *PLIN3* mRNA levels did not change significantly in NEK2A-overexpressing or NEK2A-depleted cells (Figures 4C and 4D). These results suggested that NEK2A regulates PLIN2 expression at the transcriptional level.

### Overexpression and depletion of NEK2A had no effect on LD number and size in cells

**Figure 5:**
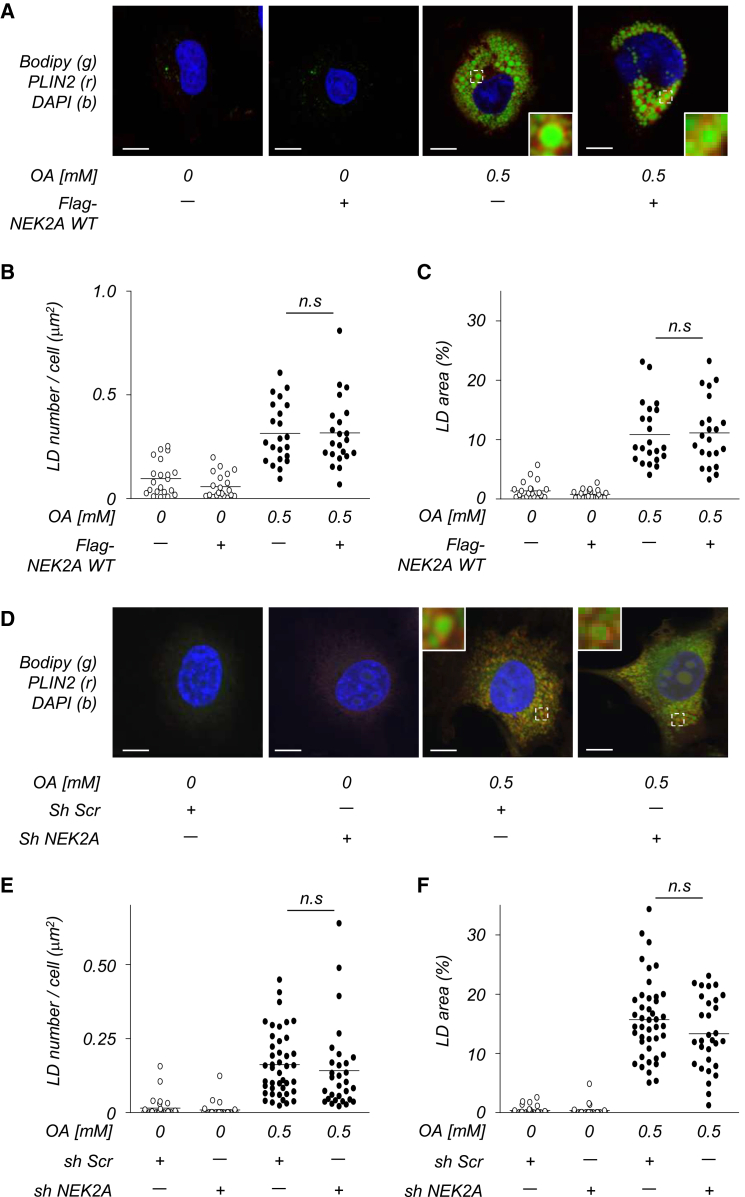
Intracellular distribution of PLIN2 in cells manipulated by NEK2A overexpression (A) HuH7 cells overexpressing either mock or Flag-NEK2A WT were incubated with or without 0.5 mM oleic acid (OA). The cells were stained for Bodipy (green, g), PLIN2 (red, r), and DAPI (blue, b). Scale bars, 10 μm. (B) LD number per cell was quantified from (A). Results are presented as scattered dot plots (mean ± SD). (C) LD area was quantified as a ratio of the total cell area from (A). (D) HuH7 cells treated with shRNA targeting NEK2A (sh *NEK2A*) or scrambled shRNA (sh Scr) were incubated with or without 0.5 mM OA. The cells were stained for Bodipy (green, g), PLIN2 (red, r), and DAPI (blue, b). Scale bars, 10 μm. (E) LD number per cell was quantified from (D). The results are shown as scattered dot plots (mean ± SD). (F) LD area was quantified as a ratio of the total cell area from (D). ns, not significant, one-way ANOVA followed by Tukey-Kramer post hoc test.

PLIN2 is known to stabilize LDs^52^^,^^53^; thus, we examined the number and total area of LDs in cells. In the absence of OA, LD signals were barely detectable and remained below the detection limit in our confocal imaging, consistent with the very low basal lipid content of HuH7 cells. In cells without OA treatment, overexpression of Flag-NEK2A WT did not significantly alter the LD number or total area. In OA-treated cells, the number and total area of LDs increased; however, there was no significant difference with or without Flag-NEK2A WT-overexpression (Figures 5A–5C). Similarly, NEK2A knockdown did not affect the LD number or area, regardless of OA treatment (Figures 5D–5F).

Additionally, PLIN3 distribution was analyzed by immunostaining. PLIN3 was found to have a ring-like structure in both NEK2A overexpressing and sh NEK2A-treated cells (Figures S3A and S3B). When PLIN2 expression was altered, PLIN3 may have compensated for PLIN2 at the LD surface.

### NEK2A kinase activity is not necessary for PLIN2 expression regulation

**Figure 6:**
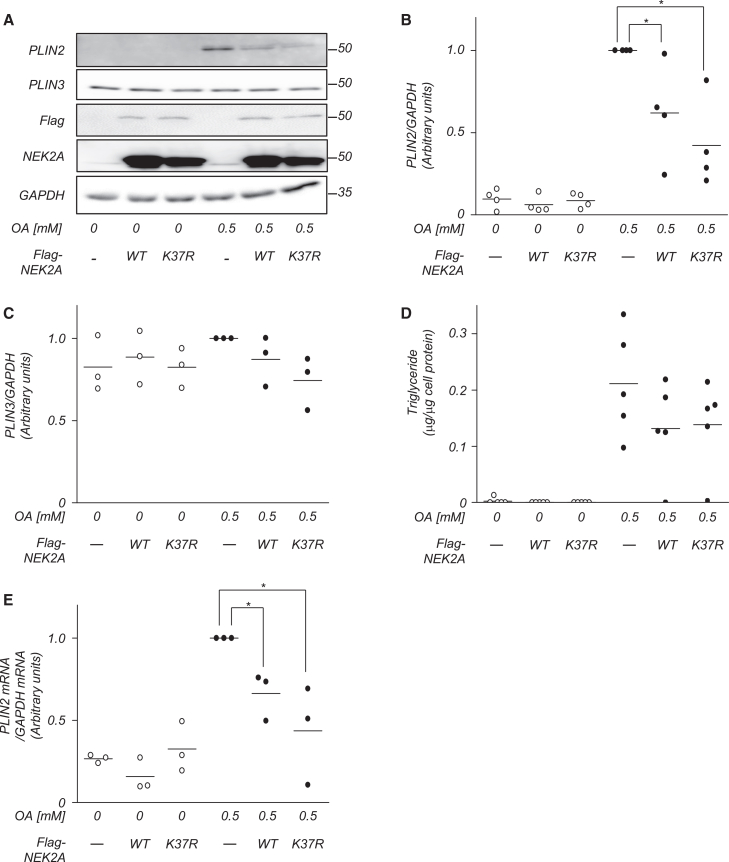
NEK2A kinase activity-independent PLIN2 expression regulation HuH7 cells overexpressing mock, Flag-NEK2A WT, or Flag-NEK2A K37R were incubated with or without 0.5 mM oleic acid (OA). (A) Cell lysates were analyzed by immunoblotting with antibodies against PLIN2, PLIN3, Flag (DYKDDDDK), NEK2A, and glyceraldehyde 3-phosphate dehydrogenase (GAPDH). Data shown are representative of at least three independent experiments. (B and C) The relative protein levels of PLIN2 and PLIN3 were quantified, normalized to those of GAPDH, and expressed in arbitrary units. The protein levels in the OA-treated mock-overexpressing cells during interphase were set to 1.0. The results are shown as scattered dot plots (mean ± SD). (D) Intracellular TG levels were quantified and presented as scattered dot plots (mean ± SD). (E) Total RNA was isolated, and RT-PCR was performed to determine the mRNA levels of *PLIN2* normalized to *GAPDH.* The expression level in OA-treated mock-overexpressing cells was set to 1.0. The results are shown as scattered dot plots (mean ± SD). ∗*p* < 0.05, one-way ANOVA followed by Tukey-Kramer post hoc test.

Since NEK2A is a Ser/Thr kinase, we investigated whether NEK2A kinase activity plays a crucial role in the regulation of PLIN2 expression. We utilized the K37R mutant of NEK2A, which is known as a kinase-dead mutant.^54^ This lysine-to-arginine substitution occurs at the conserved ATP-binding site within the catalytic domain and markedly reduces autophosphorylation and substrate phosphorylation activity *in vitro*.^55^ Overexpression of Flag-NEK2A WT significantly suppressed PLIN2 protein levels in OA-treated cells, and this suppression was also observed in cells expressing the Flag-NEK2A K37R mutant (Figures 6A and 6B). In addition, Flag-NEK2A K37R inhibited *PLIN2* mRNA expression in OA-treated cells (Figure 6E). In contrast, PLIN3 expression levels did not change significantly under any of the conditions studied (Figures 6A and 6C). TG levels slightly decreased in cells overexpressing either WT or K37R NEK2A, although the difference was not statistically significant (Figure 6D). These results suggest that NEK2A kinase activity is not essential for regulating PLIN2 expression at the protein and mRNA levels.

### Kinase domain of NEK2A is necessary for PLIN2 regulation

**Figure 7:**
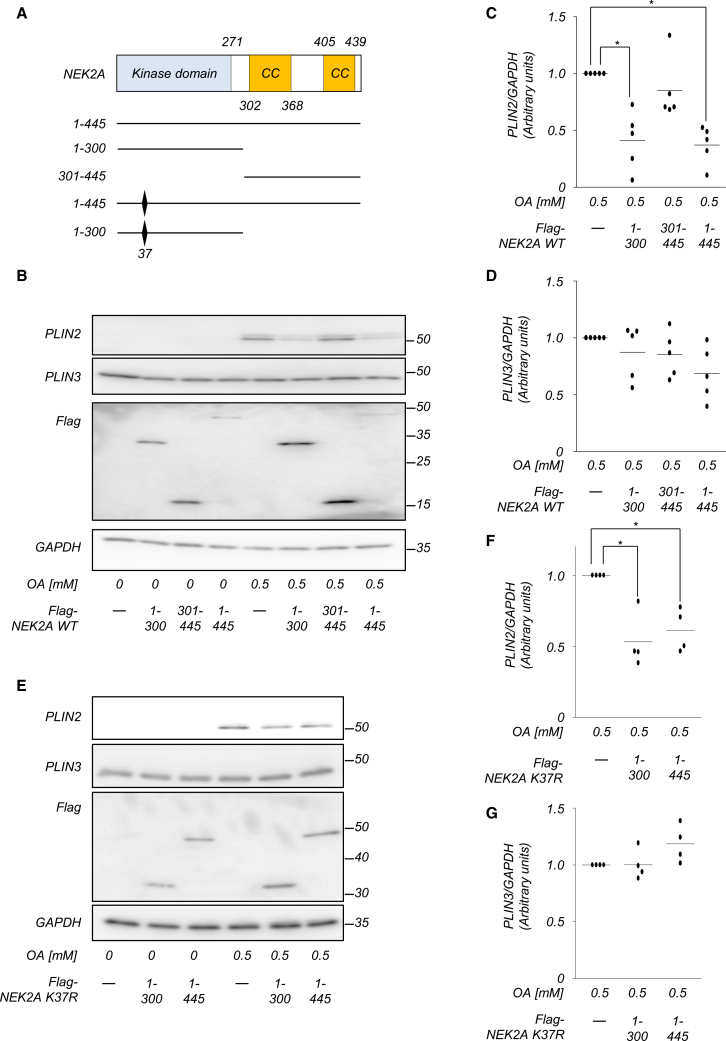
Kinase domain of NEK2 WT-dependent PLIN2 regulation (A) Schematic representation of the domain structure of NEK2A and the Flag-tagged truncation constructs used in this experiment. CC: coiled-coil domain. (B) HuH7 cells expressing the indicated Flag-NEK2A WT truncation mutants were incubated with or without 0.5 mM oleic acid (OA). Cell lysates were analyzed by immunoblotting with antibodies against PLIN2, PLIN3, Flag (DYKDDDDK), and glyceraldehyde 3-phosphate dehydrogenase (GAPDH). Data shown are representative of five independent experiments. (C and D) The relative protein levels of PLIN2 (C) and PLIN3 (D) were quantified, normalized to GAPDH, and expressed in arbitrary units. The protein levels in the OA-treated mock-overexpressing cells during interphase were set to 1.0. The results are shown as scattered dot plots (mean ± SD). (E) HuH7 cells expressing the indicated Flag-NEK2A K37R truncation mutants were incubated with or without 0.5 mM OA. Cell lysates were analyzed by immunoblotting with antibodies against PLIN2, PLIN3, Flag, and GAPDH. Data shown are representative of four independent experiments. (F and G) The relative protein levels of PLIN2 (C) and PLIN3 (D) were quantified, normalized to GAPDH, and expressed in arbitrary units. PLIN2 and PLIN3 levels in OA-treated mock-overexpressing cells during interphase were set to 1.0. The results are shown as scattered dot plots (mean ± SD). ∗*p* < 0.05, one-way ANOVA followed by Tukey-Kramer post hoc test.

NEK2A comprises a kinase domain and two coiled-coil (CC) domains.^33^^,^^56^ To further investigate the structural requirements for PLIN2 regulation by NEK2A, we constructed a Flag-tagged NEK2A full-length protein (1–445 aa) and two truncation mutants: an N-terminal fragment (1–300 aa) and a C-terminal fragment (301–445 aa) (Figure 7A). All constructs generated proteins with the predicted molecular weights and comparable expression levels (Figure 7B). Increase in PLIN2 levels after OA treatment was suppressed in cells expressing either Flag-NEK2A WT or NEK2A (1–300 aa), but not in cells expressing the C-terminal fragment of NEK2A (301–445 aa) (Figures 7B and 7C). The PLIN3 levels remained unchanged under all conditions (Figures 7B and 7D).

In order to confirm the role of the NEK2A kinase domain (but not its kinase activity) in PLIN2 expression, we generated a full-length (1–445 aa) and an N-terminally truncated (1–300 aa) construct of the NEK2A K37R mutant (Figure 7A). The constructs generated proteins with the predicted molecular weights and comparable expression levels (Figure 7E). PLIN2 levels increased after OA treatment, and PLIN2 expression decreased in cells overexpressing either NEK2A K37R (1–300 aa) or NEK2A-K37R (1–445 aa) (Figures 7E and 7F). The PLIN3 levels remained unchanged under all conditions (Figures 7E and 7G). As shown in Figure 6E, the full-length constructs of both WT and K37R mutant of NEK2A suppressed *PLIN2* mRNA expression in OA-treated cells. Furthermore, we confirmed truncated forms of NEK2A, namely Flag-NEK2A WT (1–300 aa) and Flag-NEK2A K37R (1–300 aa), suppressed *PLIN2* mRNA significantly (Figure S4). These results suggested that the NEK2A kinase domain itself, but not its kinase activity, is essential for the regulation of PLIN2 expression in cells.

### SERBP1 as a NEK2A-interacting protein

Abundant NEK2A is present in the cytosol, especially in the centrosomes, and a portion is present in the nucleus.^33^ NEK2A is known as a multifunctional protein and is reported to interact with many proteins.^57^ To the best of our knowledge, NEK2A does not contain any known DNA- or RNA-binding motif. Therefore, we hypothesized that NEK2A regulated PLIN2 expression by interacting with other proteins.

**Figure 8:**
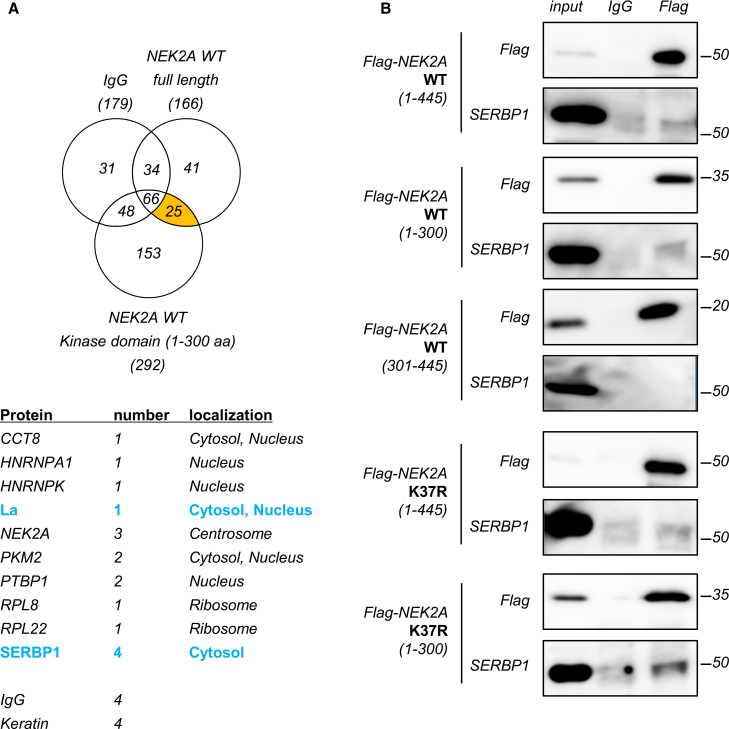
SERBP1 as a NEK2A-interacting protein (A) Venn diagram depicts the results of co-immunoprecipitation proteomics analysis. Each circle represents a group of proteins co-immunoprecipitated with either a Flag-tagged NEK2A-WT truncation mutant or control IgG. The overlap of the circles indicates proteins co-immunoprecipitated with multiple conditions. The numbers within each region indicate the number of proteins identified by LC-MS/MS. The 25 proteins identified are listed in the table. For some proteins, multiple isoforms are predicted by Protein Pilot calculation. La-related protein 3 (LARP3) is listed as “La” in the Protein Pilot program. (B) HuH7 cells express the indicated Flag-NEK2A truncation mutants were subjected to immunoprecipitation with control IgG or anti-Flag-tag antibodies. The immunoprecipitants were analyzed by immunoblotting using antibodies against Flag and SERBP1.

To identify the potential partner proteins involved in NEK2A-dependent PLIN2 regulation, we immunoprecipitated Flag-NEK2A WT and Flag-NEK2A (1–300 aa), and the co-immunoprecipitants were subjected to proteomic analysis using LC-MS/MS. As a result, 25 proteins were identified as common interactors with both full-length NEK2A and the N-terminal kinase domain fragment (1–300 aa) (Figure 8A). Among these coimmunoprecipitants, we focused on SERPINE1 mRNA-binding protein 1 (SERBP1, also known as CGI-55). SERBP1 is an RNA-binding protein^58^^,^^59^^,^^60^ and has been implicated in the progression from metabolic dysfunction-associated steatohepatitis (MASH) to HCC.^61^

We investigated whether the kinase domain of NEK2A alone interacts with SERBP1. SERBP1 co-immunoprecipitated with Flag-NEK2A WT, Flag-NEK2A (1–300 aa), Flag-NEK2A K37R (1–445 aa), and Flag-NEK2A K37R (1–300 aa), but not with Flag-NEK2A (301–445 aa) or control IgG (Figure 8B). Thus, we could conclude that the NEK2A kinase domain, but not its kinase activity, is important for the interaction between NEK2A and SERBP1 in cells.

### NEK2A-dependent PLIN2 regulation via SERBP1

**Figure 9:**
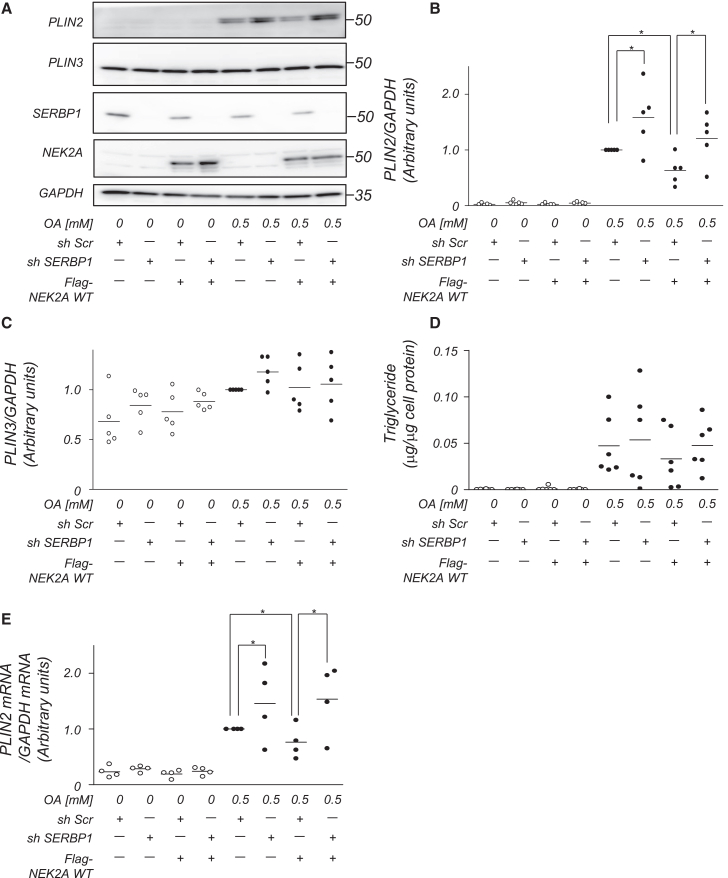
Suppressive of PLIN2 induced by NEK2A via SEBRP1 HuH7 cells expressing mock or Flag-NEK2A WT were treated with scrambled short hairpin RNA (sh Scr) or shRNA against SERBP1 (sh *SERBP1*). The cells were incubated with or without 0.5 mM oleic acid (OA). (A) Cell lysates were analyzed by immunoblotting using antibodies against PLIN2, PLIN3, SERBP1, NEK2A, and glyceraldehyde 3-phosphate dehydrogenase (GAPDH). Data shown are representative of five independent experiments. (B and C) The relative protein levels of PLIN2 (C) and PLIN3 (D) were quantified, normalized to those of GAPDH, and expressed in arbitrary units. The protein levels in OA-treated cells were set to 1.0. The results are shown as scattered dot plots (mean ± SD). (D) Intracellular TG levels were quantified and presented as scattered dot plots (mean ± SD). (E) Total RNA was isolated, and RT-PCR was performed to determine the mRNA levels of *PLIN2* normalized to *GAPDH.* The expression level in OA-treated sh Scr cells was set to 1.0. The results are shown as scattered dot plots (mean ± SD). ∗*p* < 0.05, one-way ANOVA followed by Tukey-Kramer post hoc test.

To elucidate whether SERBP1 was involved in NEK2A-dependent PLIN2 expression, we prepared sh SERBP1 and/or NEK2A-overexpressing cells. Flag-NEK2A WT overexpression significantly suppressed PLIN2 expression after OA treatment. Interestingly, this suppression was reversed by co-treatment with sh SERBP1, thus suggesting that NEK2A regulates PLIN2 expression via a SERBP1-dependent mechanism (Figures 9A and 9B). PLIN3 expression levels did not change significantly under any conditions (Figures 9A and 9C). We found no significant differences in TG levels among OA-treated cells (Figure 9D). Consistent with the protein expression data, *PLIN2* mRNA levels increased in sh SERBP1-treated cells after OA treatment, regardless of Flag-NEK2A WT overexpression (Figure 9E).

Among the 25 proteins identified in the immunoprecipitate, SERBP1 and La-related protein 3 (LARP3) are cytoplasmic RNA-binding proteins, consistent with the subcellular localization of NEK2A. To confirm that SERBP1, and not LARP3, was responsible for PLIN2 regulation, we investigated the effect of LARP3 knockdown. In sh LARP3-treated cells, *LARP3* mRNA levels decreased significantly compared to those in sh Scr-treated cells (Figure S5A). PLIN2 expression did not change significantly upon the depletion of *LARP3* mRNA (Figures S5B and S5C), suggesting that LARP3 is not involved in PLIN2 expression. Similar to other conditions, PLIN3 expression did not change significantly upon LARP3 suppression (Figures S5B and S5D).

When taken together, these results indicate that SERBP1, but not LARP3, is a NEK2A-interacting protein required for PLIN2 transcriptional regulation.

### No interaction between SERBP1 protein and PLIN2 mRNA

SERBP1 has been reported as an RNA-binding protein despite lacking a typical RNA-binding domain.^58^ To determine whether SERBP1 recognizes *PLIN2* mRNA, we performed an RNA immunoprecipitation (RIP) assay. Although Flag-SERBP1 was successfully immunoprecipitated (Figure S6A), the RNA recovered by Flag-SERBP1 was no more than that recovered by IgG and was much less relative to the total RNA in the cell lysate (Figure S6B). No significant increase in *PLIN2* mRNA was detected in co-immunoprecipitants with Flag-SERBP1 (Figure S6C). These results indicated that SERBP1 does not directly bind to *PLIN2* mRNA, suggesting that it regulates PLIN2 expression through an indirect mechanism in cells.

### SERBP1 knockdown and mRNA stability

**Figure 10:**
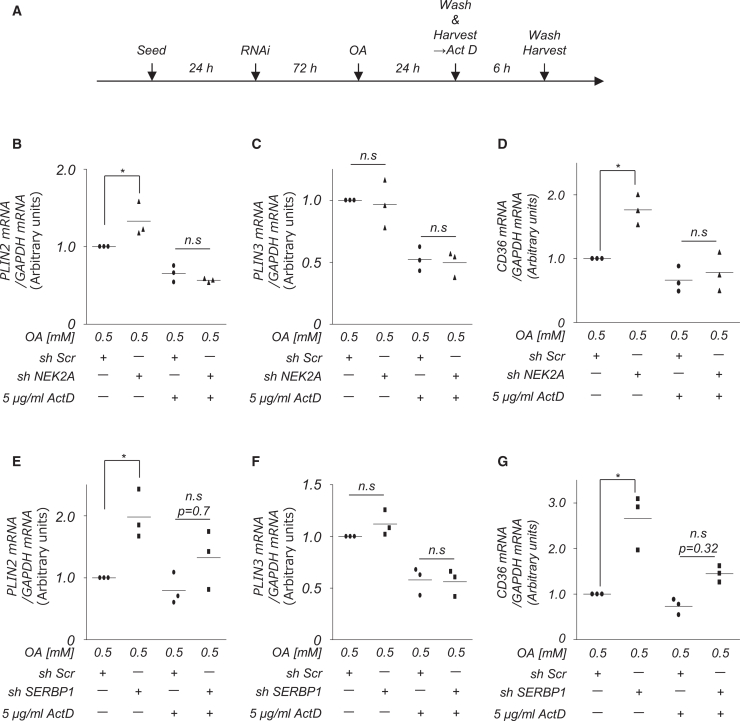
mRNA stability analysis by actinomycin D treatment (A) HuH7 cells treated with scrambled shRNA (sh Scr) or shRNA against NEK2A (sh *NEK2A*), or SERBP1 (sh *SERBP1*) were incubated with or without 0.5 mM oleic acid (OA). The cells were washed twice with PBS and then treated with or without 5 μg/mL actinomycin D for 6 h. (B–G) Total RNA was isolated, and RT-PCR was performed to determine the mRNA levels of *PLIN2* (B, E), *PLIN3* (C, F), and *CD36* (D and G), all normalized to GAPDH. The expression level in sh Scr-treated cells after OA treatment was set to 1.0. The results are shown as scattered dot plots (mean ± SD). ∗*p* < 0.05, ns; not significant, one-way ANOVA followed by Tukey-Kramer post hoc test.

Next, we investigated whether NEK2A and SERBP1 were involved in the regulation of *PLIN2* mRNA stability in cells using actinomycin D, an RNA polymerase inhibitor. Cells were treated with actinomycin D and harvested at two time points (0 and 6 h) to assess PLIN2 transcript stability (Figure 10A). *PLIN2* mRNA levels increased in sh NEK2A-treated cells compared to in sh Scr-treated cells under OA-treated conditions (Figure 10B), which is consistent with the results shown in Figure 4B. However, this increase was abrogated following actinomycin D treatment, suggesting that *PLIN2* mRNA was less stable in the absence of NEK2A (Figure 10B). In contrast to *PLIN2*, *PLIN3* mRNA was not altered by NEK2A treatment (Figure 10C). Similarly, *PLIN2* mRNA levels were higher in sh SERBP1-treated cells than in sh Scr-treated cells; this increase by sh SERBP1 was also diminished by actinomycin D treatment (Figure 10E). As expected, *PLIN3* mRNA stability was not significantly affected by SERBP1 knockdown (Figure 10F). These results suggested that the absence of NEK2A or SERBP1 increased PLIN2 expression by enhancing its transcription in cells.

PLIN2 expression is transcriptionally regulated by peroxisome proliferator-activated receptors (PPARs), namely PPARα, PPARβ/δ, and PPARγ.^62^^,^^63^ Since the NEK2A-SERBP1 axis appeared to influence these lipid metabolism-related transcriptional factors, we also examined CD36, a representative target gene of PPARγ, to examine whether this regulation occurs at the transcriptional level.^64^ Interestingly, CD36 mRNA levels were higher in both sh NEK2A- and sh SERBP1-treated cells; however, this increase by both shRNA was also diminished by actinomycin D treatment (Figures 10D and 10G). These results suggest that the NEK2A-SERBP1 axis regulates PPAR target genes in a transcriptional manner.

Collectively, these findings raised the possibility that the NEK2A-SERBP1 axis modulates PLIN2 expression through mechanisms involving PPAR signaling rather than by affecting mRNA stability.

**Figure 11:**
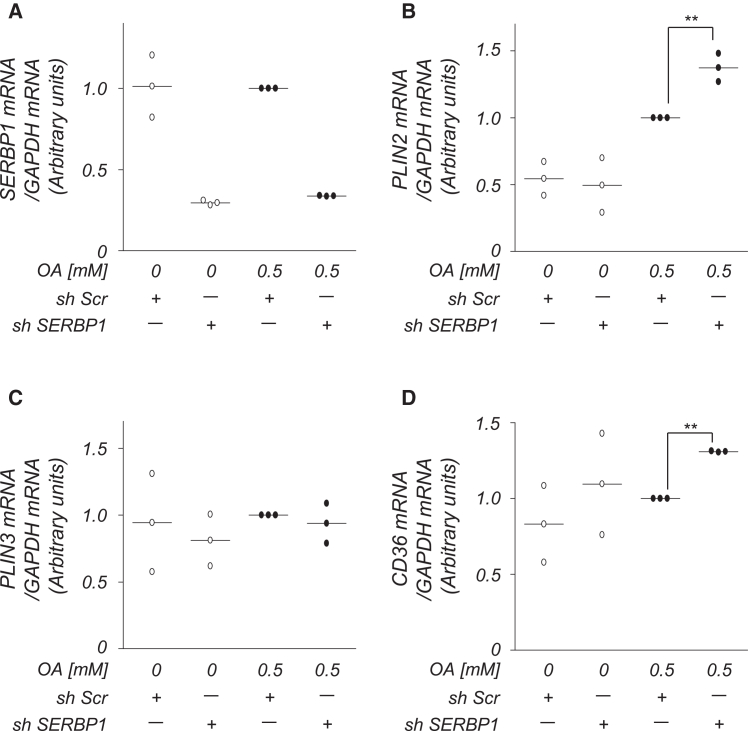
The effects of SERBP1 depletion on PPARγ target genes in HepG2 cells HepG2 cells were treated with scrambled short hairpin RNA (sh Scr) or shRNA against SERBP1 (sh *SERBP*). The cells were incubated with or without 0.5 mM oleic acid (OA). Total RNA was isolated, and RT-PCR was performed to determine the mRNA levels of *SERBP1* (A), *PLIN2* (B), *PLIN3* (C), and *CD36* (D), normalized to *GAPDH*. The expression level in OA-treated sh Scr was set to 1.0. The results are shown as scattered dot plots (mean ± SD). ∗∗*p* < 0.01, one-way ANOVA followed by Tukey-Kramer post hoc test.

To assess whether these regulatory effects are generalizable across hepatic cell models, we further examined their reproducibility in another human hepatocarcinoma, HepG2 cells. SERBP1 knockdown similarly increased PLIN2 and CD36 mRNA levels in HepG2, consistent with the trend observed in HuH7 cells (Figure 11). These data support the cross-cell line robustness of the NEK2A-SERBP1 axis and its influence on PPARγ-associated transcriptional programs.

### Inhibition of PPARγ suppresses PLIN2 expression mediated by the NEK2A-SERBP1 axis

**Figure 12:**
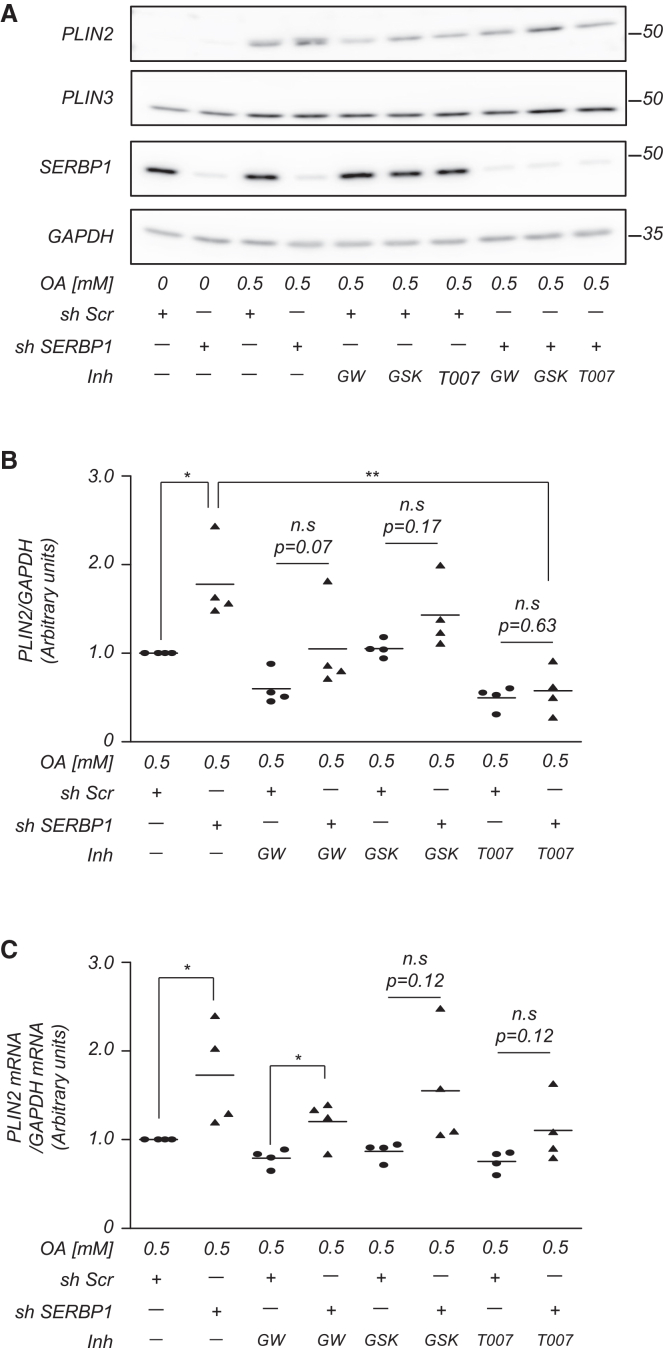
Effects of PPAR inhibitors on PLIN2 and PLIN3 expression HuH7 cells were treated with vehicle, GW6471 (a PPARα inhibitor), GSK3787 (a PPARβ/δ inhibitor), or T0070907 (a PPARγ inhibitor) and incubated with or without 0.5 mM oleic acid (OA). (A) Cell lysates were analyzed by immunoblotting using antibodies against PLIN2, PLIN3, SERBP1, and glyceraldehyde 3-phosphate dehydrogenase (GAPDH). Data shown are representative of four independent experiments. (B) The relative protein levels of PLIN2 were quantified, normalized to those of GAPDH, and expressed in arbitrary units. PLIN2 levels in vehicle-treated cells with OA were set to 1.0. The results are shown as scattered dot plots (mean ± SD). (C) Total RNA was isolated, and RT-PCR was performed to determine the mRNA levels of *PLIN2* normalized to *GAPDH.* The expression level in vehicle-treated cells with OA was set to 1.0. The results are shown as scattered dot plots (mean ± SD). Inh: Inhibitor GW:GW6471 GSK: GSK3787 T007: T0070907. ∗*p* < 0.05, ∗∗*p* < 0.01, ns, not significant, one-way ANOVA followed by Tukey-Kramer post hoc test.

In order to further investigate the role of PPAR signaling in the SERBP1-mediated transcriptional regulation of PLIN2, we treated cells with each PPAR inhibitor targeting either PPARα, PPARβ/δ, or PPARγ. Among these, the PPARγ inhibitor T0070907 exhibited the strongest suppressive effect on both the PLIN2 protein and mRNA levels (Figure 12). In sh SERBP1-treated cells, both protein and mRNA of PLIN2 were increased after OA treatment; however, this increase was almost abolished in the presence of the PPARγ inhibitor. These results suggest that PPARγ plays an essential role in NEK2A-SERBP1-mediated PLIN2 expression in cells.

Since pharmacological inhibition suggested that PPARγ activity is required for NEK2A-SERBP1-dependent PLIN2 expression, we next asked whether this regulation was accompanied by changes in the overall expression of PPARγ. Immunoblot analysis revealed that the total PPARγ protein level remained largely unchanged in SERBP1-depleted cells (Figures S7A and S7B), indicating that SERBP1 does not markedly affect PPARγ abundance. These results suggest that SERBP1 may not regulate PPARγ directly.

### OA treatment-independent intracellular SERBP1 localization

Finally, to clarify whether NEK2A also influences the intracellular distribution of its downstream partner, we analyzed the subcellular localization of SERBP1. SERBP1 was absent from LD fractions (fractions 1 and 2) and was only recovered in the bottom fractions (fractions 8–14), indicating that SERBP1 is not a component of LDs in cells. This localization did not change significantly upon NEK2A (Figure S8A).

We investigated SERBP1 intracellular localization using a microscope. In sh Scr-treated cells, SERBP1 was found throughout the cytoplasm with a portion forming foci (Figure S8B). In the sh SERBP1-treated cells, both diffuse cytoplasmic and foci-like signals were reduced, suggesting that SERBP1 is normally localized to specific cytoplasmic regions. This localization was also observed after OA treatment (Figure S8C); however, the SERBP1 signal did not colocalize with NEK2A (Figure S8D). Together, these findings indicate that NEK2A and OA treatment do not alter the overall localization of SERBP1.

## Discussion

We identified a regulatory pathway in which the NEK2A-SERBP1 axis controls PLIN2 expression independently of lipid accumulation, revealing an unrecognized mechanism of PLIN2 regulation. We previously demonstrated that intracellular LDs are transferred to daughter cells without losing their spherical structure during mitosis.^27^ Simultaneously, PLIN2 protein levels decrease in mitotic cells compared with those in interphase cells.^27^ In addition, we previously found that the centrosome cycle is controlled by a temporal change in the expression level of Ser/Thr kinase NEK2A.^28^ These findings prompted us to investigate whether NEK2A contributes to mitotic PLIN2 downregulation.

To determine the effect of NEK2A depletion on PLIN2 expression, we examined PLIN2 levels in mitotic cells. Consistent with earlier observations, PLIN2 levels were reduced in the mitotic cells (Figures 1A and 1B). Interestingly, NEK2A knockdown reversed this mitotic reduction and elevated PLIN2 levels in interphase cells (Figures 1A and 1B). NEK2A consists of an N-terminal kinase domain and a C-terminal CC domain.^33^^,^^56^ Through domain mapping, we demonstrated that the N-terminal kinase domain (1–300 aa) of NEK2A, but not the CC domain (301–445 aa), was sufficient to suppress PLIN2 expression (Figures 7B and 7C). Furthermore, the kinase domain (1–300 aa) containing the kinase-dead NEK2A mutation K37R^54^ also suppressed OA-induced PLIN2 protein expression in cells (Figures 7E and 7F). Consistently, RT-PCR analysis demonstrated that the truncated construct of kinase-dead NEK2A K37R (1–300 aa) suppressed *PLIN2* mRNA expression to a similar extent as the corresponding WT construct (Figure S4). These results suggest that domain structure, but not catalytic activity, is required for PLIN2 regulation by NEK2A.

To clarify whether NEK2A downregulates PLIN2 through (1) protein degradation or (2) transcriptional repression, we first examined the possibility of protein degradation. Immunoprecipitation assays revealed that neither PLIN2 nor PLIN3 interacted with NEK2A (Figure 3A). Moreover, treatment with the proteasome inhibitor MG132 or the autophagy inhibitor chloroquine failed to restore PLIN2 levels reduced by NEK2A overexpression (Figures S1A and S1B). Although both inhibitors slightly increased basal PLIN2 expression, they did not prevent the reduction of PLIN2 induced by NEK2A overexpression, suggesting that proteasomal or lysosomal degradation is not the major regulatory route. These findings indicate that NEK2A does not significantly regulate PLIN2 through protein degradation, and we next examined whether NEK2A affects PLIN2 transcription indirectly. We found that NEK2A regulates PLIN2 protein and mRNA levels independently of its kinase activity, at least in part through its interactions with SERBP1.

NEK2A has recently been implicated in various cellular functions, such as autophagy and anticancer drug resistance, in addition to its role in mitosis.^36^^,^^37^^,^^38^ However, NEK2A has not been reported to bind directly to any specific partner molecules, including DNA, RNA, or lipids, suggesting that NEK2A may regulate gene expression indirectly through other proteins. We performed proteomic analysis to identify NEK2A-interacting proteins involved in PLIN2 transcriptional regulation. Among the identified candidates, SERBP1 is an RNA-binding protein but lacks typical RNA-binding motifs.^58^ SERBP1 is highly expressed in glioblastoma cells, and it is associated with poor prognosis.^65^ Recent studies have implicated SERBP1 in the progression of MASH (formerly NASH) to HCC.^61^ Gene Ontology analysis has shown that SERBP1 interacts with several proteins, including stress granules, P-bodies, and Cajal bodies.^66^^,^^67^^,^^68^ Stress granules and P bodies have been shown to be cytoplasmic, monolayer ribonucleoprotein granules involved in post-transcriptional regulation, particularly RNA degradation.^66^^,^^67^ In contrast, Cajal bodies are subnuclear structures involved in transcription and RNA processing.^66^^,^^68^ Given these reported roles in RNA metabolism, we next examined whether SERBP1 directly interacts with *PLIN2* mRNA in our experimental system.

SERBP1 was reported to be an mRNA-binding protein; however, it did not bind to *PLIN2* mRNA (Figure S6). It has been reported that SERBP1 interacts with *PLIN2* mRNA in HeLa cells by the RIP assay.^69^ In that study, GST-tagged SERBP1 was stably overexpressed, and the cells were synchronized to a specific cell cycle phase. In contrast, our experiments were performed in HuH7 cells under asynchronous conditions using exogenously expressed Flag-tagged SERBP1. These differences in cell type, SERBP1 expression levels, and cell cycle status may account for the discrepancies seen between our findings and those previously reported. Furthermore, Baudin et al., reported that the sequence 5′-GCGCGGG-3′ was identified as a SERBP1 consensus binding motif,^58^ however, this sequence is absent in the PLIN2 cDNA sequence.^70^

*PLIN2* mRNA levels were increased both in sh NEK2A-treated and sh SERBP1-treated cells compared to those in sh Scr-treated cells following OA treatment, and this increase was abolished by actinomycin D (Figures 10B and 10E), suggesting that NEK2A and SERBP1 suppress PLIN2 transcription. Although *PLIN2* mRNA stability modestly decreased in the absence of NEK2A and SERBP1, the primary mechanism underlying PLIN2 upregulation in the knockdown cells was likely to be the exclusion of transcriptional repression. These results support a model in which NEK2A regulates PLIN2 expression primarily at the transcriptional level rather than through protein degradation or altering mRNA stability.

PLIN2 expression is transcriptionally regulated by PPARs.^71^ Using PPAR inhibitors (α, β/δ, and γ), we demonstrated that PPARγ inhibitor, T0070907, exerted the strongest suppressive effect on PLIN2 expression. The increase in PLIN2 expression in SERBP1 knockdown cells was abolished in the presence of PPARγ inhibitor (Figure 11), suggesting that SERBP1 may function as a modulator of PPARγ activity. PPARs are activated by fatty acids, including OA, and PLIN2 upregulation by NEK2A or SERBP1 knockdown was not observed in the absence of OA. Given that fatty acids, including OA, are physiological ligands of PPARs. Our results suggest that SERBP1 is a modulator of activated PPARγ, which is a key transcriptional regulator of PLIN2. To further test this idea, we examined PPARγ target gene expression. CD36 mRNA, a representative downstream target of PPARγ, was analyzed as an indicator of transcriptional activity. In both NEK2A- and SERBP1-depleted cells, the effects of actinomycin D on CD36 transcripts were similar to what we observed for *PLIN2* mRNA (Figures 10D and 10G). These results further support a role of the NEK2A-SERBP1 axis in influencing PPARγ-associated transcriptional activity. Furthermore, we investigated whether this observed regulation was cell line-specific and found that *PLIN2* and *CD36* mRNA were increased in sh SERBP1-treated HepG2 cell lines (Figure 11).

Neither OA treatment nor NEK2A overexpression altered SERBP1 protein expression in the cells (Figures 9 and S8A). SERBP1 was distributed throughout the cytoplasm with a portion forming discrete foci (Figure S8B). Because NEK2A localizes to the centrosome in cells, we investigated whether SERBP1 co-localizes with NEK2A at that site. Although both NEK2A and SERBP1 appeared as punctate signals under the microscope, NEK2A was confined to a single focus, whereas SERBP1 formed multiple cytoplasmic foci that did not overlap with the centrosome. Thus, we speculated that only a portion of SERBP1 could interact with NEK2A at a time, and the NEK2A-SERBP1 complex functions in the cytoplasm, but not at the centrosome, in regulating PLIN2 expression.

Large tumor suppressor kinase 1 (LATS1), a Ser/Thr kinase belonging to the protein kinase A/G/C family, regulates cell toxicity through functional interactions with the second mitochondria-derived activator of caspases (SMACs).^72^ Although the physical nature of the LATS1–SMAC association remains unclear, these proteins have been proposed to form a functional complex that is critical for inducing cytotoxic responses. Interestingly, this interaction is dependent on the kinase domain of LATS1, but it does not require its enzymatic activity. This mode of interaction is reminiscent of the NEK2A-SERBP1 complex observed in our study, in which the kinase domain of NEK2A is required for binding to SERBP1, whereas its catalytic activity is dispensable. By analogy, we can speculate that NEK2A and SERBP1 may also operate as functional complexes in the cytoplasm, even in the absence of direct enzymatic action, to regulate PLIN2 expression. Although we were unable to determine the precise subcellular location of the NEK2A-SERBP1 complex, it is plausible that only a minor fraction of either protein participates in this functional interaction. The complex may exist transiently or be masked within heterogeneous cytoplasmic compartments, making visualization technically challenging using confocal microscopy.

Next, we investigated whether NEK2A-SERBP1 affects PPARγ expression and potential activity. The total amount of PPARγ protein remained essentially unchanged with or without SERBP1 depletion (Figure S7). Although total expression was stable, subtle alterations in its subcellular distribution or transcriptional activity cannot be excluded. This observation suggests that SERBP1 may modulate PPARγ function primarily at the level of activity or localization, rather than through changes in overall abundance. Because PPARγ activity is tightly linked to its nuclear translocation upon ligand stimulation, it is also possible that SERBP1 indirectly affects the efficiency or dynamics of PPARγ nuclear import. Even modest changes in this process could account for the altered transcriptional outputs observed for PLIN2 and CD36. PPARγ activity is known to be modulated by multiple nuclear receptor co-regulators. For example, PGC-1α coactivates PPARγ to control mitochondrial and lipid metabolism,^73^^,^^74^ while SRC-1/2 (NCOA1/2) act as versatile coactivators of nuclear receptors, including PPARγ.^75^ In contrast, corepressors such as NCOR1 and SMRT (NCOR2) can attenuate PPARγ-dependent transcription and modulate insulin sensitivity.^76^ Such mechanisms are consistent with our finding that SERBP1 depletion selectively altered PPARγ target genes such as PLIN2 and CD36.

Comparable kinase domain-independent roles have also been reported for other NEK family members. For example, NEK6 has been implicated in cell cycle progression and apoptosis, and although not explicitly termed “kinase domain-independent,” studies have suggested domain-mediated functions beyond its catalytic activity.^39^ More recently, kinase-dead NEK6 mutants were shown to retain specific subcellular localization and binding ability, indicating a potential non-canonical mechanism.^39^ Likewise, NEK9 participates in spindle assembly through its N-terminal domain, acting as a scaffold independently of its kinase function.^30^^,^^39^ These precedents suggest that kinase domain-dependent functions may be a broader property within the NEK family, although the underlying mechanisms differ. Our findings, therefore, expand this emerging concept by identifying a kinase domain-dependent NEK2A-SERBP1 axis that potentially modulates PLIN2 transcription.

The current finding that the NEK2A-SERBP1 axis regulates PLIN2 expression without affecting LD number or size (Figure 5) suggests that PLIN2 may have functions in cells beyond its classical role in lipid storage. This possibility is supported by previous studies. Garcia et al. reported that PLIN2 is associated with abnormal spindle formation involving multiple poles,^77^ while Ramosaj et al. showed that the heterologous expression of PLIN2 in neural stem cells regulates cell proliferation and differentiation.^26^ These findings raise the possibility that PLIN2 contributes to cellular processes independent of TG metabolism and that its expression is modulated accordingly.

While these findings point to lipid-independent roles of PLIN2, its well-established function in LD stabilization remains critical in the context of metabolic diseases. Hepatic LD accumulation is a clinical symptom of conditions, such as MASH or obesity.^4^^,^^50^ Among the LD components, PLIN2 is a major LD surface protein that stabilizes LDs by protecting them from autophagic degradation.^52^^,^^53^ PLIN2 overexpression causes LD accumulation in cells.^5^^,^^6^ PLIN2-knockout mice showed resistance to obesity and MASH when fed a methionine-choline-deficient diet.^78^ We demonstrated that NEK2A regulated PLIN2 expression via SERBP1. Since SERBP1 has been implicated in the progression from MASH to HCC^61^ and NEK2A expression is associated with poor prognosis in patients with cancer,^36^^,^^38^ it is possible that the NEK2A-SERBP1-mediated modulation of PLIN2 expression contributes to this pathological progression.

### Limitations of the study

Although the precise mechanism by which SERBP1 regulates PLIN2 transcription remains unclear, our findings suggest that the NEK2A-SERBP1 axis may be associated with the PPARγ-dependent transcriptional regulation of PLIN2. Several issues remain to be addressed. First, whether SERBP1 influences the nuclear translocation and DNA-binding capacity of PPARγ requires further investigation using subcellular fractionation and immunofluorescence analyses. Second, chromatin immunoprecipitation (ChIP) assays will be necessary to determine whether PPARγ directly binds to the PLIN2 promoter under NEK2A- or SERBP1-manipulated conditions. Third, because this study is primarily based on *in vitro* experiments using hepatoma cell lines, validation in *in vivo* models will be required to establish physiological relevance. Finally, confirmation in primary hepatocytes or patient-derived cells will strengthen the clinical significance of these findings.

## Resource availability

### Lead contact

Further information and requests for resources and reagents should be directed to and will be fulfilled by the lead contact, Tomohiko Makiyama (t-maki@pharm.showa-u.ac.jp).

### Materials availability

This study did not generate unique reagents.

### Data and code availability

• The raw uncropped immunoblot images supporting this study have been deposited in Mendeley Data and are available at https://doi.org/10.17632/yh446nk38m.1.
• This paper does not report any original code.
• Any additional information required to reanalyze the data reported in this paper is available from the lead contact upon request.

## Acknowledgments

We thank Takako Ueda (Clinical Medicine Joint Research Laboratory, Showa Medical University) for DNA sequencing. This study was supported by the Showa University Grant for Young Researchers and the 10.13039/501100001691JSPS 10.13039/501100001691KAKENHI grant number 24K10084.

## Author contributions

T.M. designed the study, performed all experiments, analyzed the data, directed the project, and prepared the manuscript. T.A. participated in data acquisition and performed the proteomic analysis. T.O. participated in the interpretation of the data and discussion. M.C., Y.A., A.Y., and K.S. participated in data acquisition for the cellular experiments. T.M. participated in the interpretation of the data and discussion. H.I. wrote the manuscript. All authors have read and agreed to the published version of the manuscript.

## Declaration of interests

The authors declare no competing interests.

## STAR★Methods

### Key resources table

**Table:** 

REAGENT or RESOURCE	SOURCE	IDENTIFIER
**Antibodies**		

Anti-PLIN2 antibody	Proteintech	15294-1-AP
Anti-PLIN3 antibody	Proteintech	10694-1-AP
Anti-NEK2A antibody	BD Bioscience	610593
Anti-SERBP1 antibody	Proteintech	10729-1-AP
Anti-SERBP1 antibody	Thermo Fisher Scientific	A303938AT
Anti-GAPDH antibody	Sigma-Aldrich	G9545

**Chemicals, peptides, and recombinant proteins**		

RO-3306	Sigma-Aldrich	SML0569
Bodipy493/503	Invitrogen	D3922
LipidTox Red	Invitrogen	H34476
DAPI	Dojindo	340–07971
Oleic acid	Sigma	O1383
MG132	Selleck	S2619
Chloroquine	Selleck	S6999
GW6471	Cayman	880635-03-0
GSK3787	Cayman	188591-46-0
T0070907	Cayman	10026

**Critical commercial assays**		

RIP-Assay kit	MBL	RN1001
Nucleospin RNA II	Takara	U0955B
PowerUp SYBR Green Master Mix	Applied Biosystems	A25742

**Deposited data**		

All data generated in this study	This paper	Available upon request

**Experimental models: Cell lines**		

HuH7 cells	JCRB Cell Bank	JCRB0403
HepG2 cells	ATCC	HB-8065

**Recombinant DNA**		

SERBP1 expression vector	VectorBuilder	VB900122-0080suf
pSilencer 2.1-U6 Pro	Thermo Fisher Scientific	Am5762
pENTR4 vector	Thermo Fisher Scientific	A10465
pAd/CMV/V5-DEST	Invitrogen	V49320

**Software and algorithms**		

EZR (Easy R)	Ref. 83	N/A
ImageJ/Fiji	NIH	N/A
GraphPad Prism 4	GraphPad Software	N/A

### Experimental model and study participant details

#### Cell lines

Hepatoma cell lines HuH7 and HepG2 were used in this study. HuH7 cells were obtained from JCRB Cell Bank, and HepG2 cells were obtained from ATCC. Cells were maintained under standard culture conditions as described below. HuH7 cells were routinely tested for mycoplasma contamination and confirmed to be negative. HepG2 cells were not specifically tested for mycoplasma contamination in this study but were maintained under standard aseptic conditions. Cell line authentication was not performed in this study.

### Method details

#### Cell culture

HuH7 cells and HepG2 cells were cultured in Dulbecco’s Modified Eagle’s Medium supplemented with 10% fetal bovine serum (Gibco, Waltham, MA, USA), 100 U/ml penicillin, and 100 μg/mL streptomycin at 37°C in a 5% CO_2_ incubator. In order to synchronize cells at the G2/M boundary, cells were treated with 9 μM RO-3306 (Sigma-Aldrich, St. Louis, MO, USA; a selective cyclin-dependent kinase 1 inhibitor) for 20 h as described previously.^27^^,^^79^

#### Plasmid and recombinant adenovirus

Total RNA was isolated from HuH7 cells, and cDNA fragments encoding the open reading frame of human NEK2A wild-type (WT) cells were amplified by reverse transcription PCR. The NEK2A K37R mutation was introduced using Phusion polymerase (New England Biolabs, Ipswich, MA, USA) with primers (forward primer: 5′ gcaagatattagtttggaGGgaacttg 3,’ reverse primer: 5′ tggagccatagtcaagttcCCtccaaa 3′). Truncated forms of NEK2A, including NEK2A WT (1–300 aa), NEK2A WT (301–445 aa), and NEK2A K37R (1–300 aa), were generated using Phusion Polymerase. cDNA fragments encoding the open reading frame of human SERBP1 (pRPExp-Neo-CAG>hSERBP1NM_001018067.2) were purchased from Vector Builder (Chicago, IL, USA). The vector ID was VB900122-0080suf, which could be used to retrieve detailed information about the vector from vectorbuilder.com.

For RNA interference (RNAi), short hairpin RNAs (shRNAs) targeting PLIN2, PLIN3, NEK2A, or SERBP1 were cloned into pSilencer 2.1-U6 Pro vector (Thermo Fisher Scientific, Waltham, MA, USA). The shRNA sequences are listed in Table S1. All of the constructs were subcloned into the pENTR4 vector.

To generate adenoviral vectors, these sequences were introduced into pAd/CMV/V5-DEST vector (Invitrogen, Carlsbad, CA, USA) using the Gateway system. Recombinant adenoviruses were generated according to manufacturer’s instructions. For overexpression and RNAi, cells were infected with each virus for 48 h or 72 h at 37°C in a 5% CO_2_ incubator, respectively.

#### Fluorescence microscopy

The cells grown on coverslips were fixed in 4% paraformaldehyde (Nacalai Tesque, Kyoto, Japan) for 10 min at 20°C–25°C, followed by permeabilization with 0.1% (w/v) Triton X-100 in phosphate-buffered saline (PBS) for 10 min at room temperature. For PLIN3 staining, the cells were fixed in a mixture of 2% paraformaldehyde and 2% glutaraldehyde. The permeabilized cells were blocked in PBS containing 1% bovine serum albumin (BSA) at room temperature for 1 h. The cells were incubated with antibodies against the indicated proteins (Tables S2 and S3) in PBS at room temperature for 1 h. After washing twice with PBS, the cells were treated with secondary antibodies in PBS at room temperature for 1 h. To visualize LDs and nuclei, cells were stained with Bodipy493/503 or LipidTox Red (Invitrogen, Carlsbad, CA, USA) and DAPI (Dojindo, Kumamoto, Japan), respectively. Cells were observed under a confocal laser scanning microscope (FV10i and FV1200; Olympus, Tokyo, Japan).

We repeated the experiment three times and analyzed more than five cells per experiment, resulting in at least 15 analyzed cells. The number and area of LDs and the cytosolic area were calculated using ImageJ software (NIH).

#### Subcellular fraction

Subcellular fractionation was performed as described previously.^27^^,^^46^^,^^47^ Briefly, HuH7 cells were suspended in buffer A (20 mM Tris (pH7.4) containing 1 mM EDTA, 250 mM sucrose, and a protease inhibitor cocktail (Sigma-Aldrich, St. Louis, MO, USA)) and homogenized with 25 strokes using a 27G needle. The sample was kept at 0°C–4°C during the whole process. The homogenate was centrifuged at 3,500 × *g* for 10 min, and the supernatant was used as the post-nuclear fraction (PNS). The sucrose concentration of the PNS was adjusted to 26% and then loaded into the middle of a 2–51% stepwise sucrose gradient (2%, 10%, 18%, the PNS (adjusted to 26%), 35%, 43%, and 51%). The gradient was centrifuged at 110,000 × *g* for 2 h at 4°C, using an RPS40T rotor (Hitachi Koki, Ibaraki, Japan), and aliquots were collected from the top of the gradient.

#### Protein-protein interaction analysis

Cells overexpressing the N-terminal DYKDDDDK (Flag)-fused NEK2A WT (Flag-NEK2A WT) or the Flag-fused NEK2A kinase domain were lysed with immunoprecipitation buffer (50 mM Tris (pH7.5), 150 mM NaCl, 1% (w/v) Triton X-100, a protease inhibitor cocktail (Sigma-Aldrich)), and rotated at 4°C for 30 min. After centrifugation at 15,000 × *g* for 10 min, supernatants were recovered and incubated with antibodies for 4 h at 4°C. The immunocomplexes were precipitated using Protein G Sepharose (GE Healthcare, Waltham, MA, USA), washed three times with immunoprecipitation buffer, and eluted with elution buffer (250 mM glycine, pH2.0 and a protease inhibitor cocktail (Sigma-Aldrich)). The eluate containing Flag-NEK2A WT or Flag-fused NEK2A kinase domain co-immunoprecipitants was fragmented by on-membrane trypsin digestion of the protein, as described previously.^80^ The digested proteins were analyzed by LC-MS/MS using a TripleTOF 5600 mass spectrometer (Sciex, Framingham, MA, USA). MS data were analyzed using ProteinPilot to identify proteins.

#### Immunoblotting

To prepare the cell lysate, cells were treated with lysis buffer (50 mM Tris (pH7.5), 150 mM NaCl, 0.1% (w/v) Triton X-100, and a protease inhibitor cocktail), sonicated for 10 s on ice using UR-20P (Tomy Seiko, Tokyo, Japan), and centrifuged at 15,000 × *g* at 4°C for 10 min. The protein concentration in the cell lysates was determined using a bicinchoninic acid protein assay kit (Thermo Fisher Scientific). Aliquots of cell lysates were subjected to sodium dodecyl sulfate-polyacrylamide gel electrophoresis (SDS-PAGE), followed by immunoblotting. The bands were visualized using the ECL Prime Western Blotting System (Cytiva, Marlborough, MA, USA) and recorded using LAS500 (GE Healthcare). Glyceraldehyde 3-phosphate dehydrogenase (GAPDH) was used as the loading control. The intensity of each immunoreactive band was measured using the FIJI/ImageJ software. The commercial antibodies used for immunoblotting are listed in Table S2.

#### Triglyceride measurement

Lipids were extracted from the cells using the Folch method,^81^ followed by resuspension in isopropanol. The amount of TG in one-quarter of the extract was measured using the Triglyceride E test (Fujifilm Wako, Osaka, Japan). The TG levels in each fraction were normalized to the protein concentration in the same fraction.

#### Thin-layer chromatography (TLC)

Lipids were extracted from the cells using the Bligh and Dyer extraction method,^82^ followed by resuspension in a chloroform:methanol (1:1) solution. The organic phases were spotted on a TLC silica gel 60 plate (Merck, Darmstadt, Germany) and analyzed using petroleum ether:diethyl ether:acetic acid (80:20:1) as the mobile phase. The TLC plate was stained with iodine vapor and recorded using LAS500. The intensity of each spot band was calculated using FIJI/ImageJ software with triolein (TCI, Tokyo, Japan) as the standard curve. Organic solvents were purchased from Fujifilm Wako (Osaka, Japan).

#### RNA immunoprecipitation (RIP) assay

The RIP assay was performed using a RIP-Assay kit (MBL, Tokyo, Japan; catalog no. RN1001), according to the manufacturer’s instructions. Briefly, the cells were lysed with RIP assay buffer and rotated at 4°C for 10 min. After centrifugation was performed at 12,000 × g for 10 min, the supernatants were collected and incubated with anti-Flag antibody for 4 h at 4°C. The immunocomplexes were precipitated using Protein G Sepharose (GE Healthcare), washed thrice with RIP wash buffer, and eluted.

#### RT-PCR

Total RNA was isolated from cells using Nucleospin RNA II (Takara, Shiga, Japan). RNA samples were reverse-transcribed using MuLV reverse transcriptase (Applied Biosystems, Foster City, CA, USA) in a total volume of 20 μL, and RT-PCR was performed using the StepOnePlus Real-Time PCR system (Applied Biosystems). Aliquots of the reverse transcription products were amplified in 20 μL of a reaction mixture containing PowerUp SYBR Green Master Mix (Applied Biosystems) and 0.5 μM of each primer. The primer pairs used are listed in Table S4.

For the mRNA stability assay, cells were incubated with or without 5 μg/mL actinomycin D for 6 h. Total RNA was isolated from the cells as described above. The RNA samples were reverse-transcribed and RT-PCR was performed as aforementioned.

### Quantification and statistical analysis

Data are presented as mean ± SD. Statistical analyses were performed using one-way analysis of variance followed by the Tukey–Kramer post hoc test using Easy R.^83^ The value of n represents independent experiments, as indicated in the figure legends. Statistical significance was defined as ∗*p* < 0.05.
