# Supplementary material for: NEK2A regulates PLIN2 expression through a SERBP1 dependent pathway
Source: iScience. 2026 Jun 9;29(6):116254. doi: 10.1016/j.isci.2026.116254 (PMC13264020; doi:10.1016/j.isci.2026.116254)

iScience, Volume *29*

Supplemental information

NEK2A regulates PLIN2 expression through a SERBP1 dependent pathway

Tomohiko Makiyama, Toshihiro Aiuchi, Tomoko Mikajiri, Takashi Obama, Masahiro Chatani, Yuki Azetsu, Atsushi Yamada, Kiyohito Sasa, and Hiroyuki Itabe

Figure S1

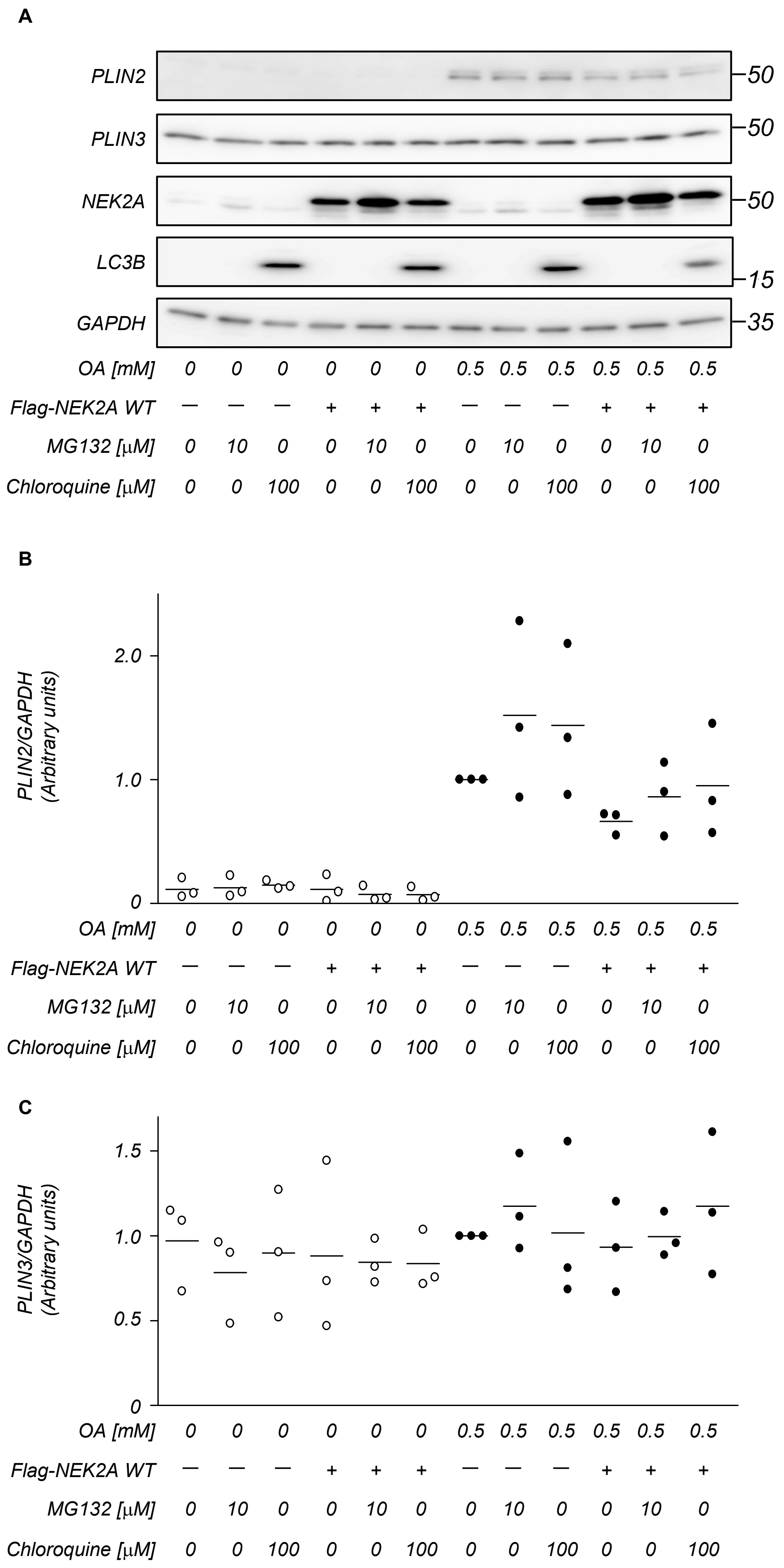
A
PLIN2
PLIN3
NEK2A
LC3B
GAPDH
50
50
50
15
35
OA [mM] 0 0 0 0 0 0 0.5 0.5 0.5 0.5 0.5 0.5
Flag-NEK2A WT — — — + + + — — — + + +
MG132 [μM] 0 10 0 0 10 0 0 10 0 0 10 0
Chloroquine [μM] 0 0 100 0 0 100 0 0 100 0 0 100
B
PLIN2/GAPDH (Arbitrary units)
0
1.0
2.0
OA [mM] 0 0 0 0 0 0 0.5 0.5 0.5 0.5 0.5 0.5
Flag-NEK2A WT — — — + + + — — — + + +
MG132 [μM] 0 10 0 0 10 0 0 10 0 0 10 0
Chloroquine [μM] 0 0 100 0 0 100 0 0 100 0 0 100
C
PLIN3/GAPDH (Arbitrary units)
0
0.5
1.0
1.5
OA [mM] 0 0 0 0 0 0 0.5 0.5 0.5 0.5 0.5 0.5
Flag-NEK2A WT — — — + + + — — — + + +
MG132 [μM] 0 10 0 0 10 0 0 10 0 0 10 0
Chloroquine [μM] 0 0 100 0 0 100 0 0 100 0 0 100

Figure *S*1. PLIN2 degradation is unaffected by proteasome or autophagy inhibition in NEK2A-overexpressing cells

(A) HuH7 cells overexpressing either mock or Flag-NEK2A WT were incubated with or without 0.5 mM oleic acid (OA). Prior to harvesting, cells were treated with or without 10 μM MG132 or 100 μM chloroquine for 1 h. Cell lysates were analyzed by immunoblotting using antibodies against PLIN2, PLIN3, NEK2A, LC3B, and glyceraldehyde 3-phosphate dehydrogenase (GAPDH). Data shown are representative of three independent experiments.

(B, C) The relative protein levels of PLIN2 and PLIN3 were quantified, normalized to GAPDH, and expressed in arbitrary units. Protein levels in mock-overexpressing cells with OA treatment were set to 1.0. The results are shown as scattered dot plots (mean ± SD).

Figure S2

A

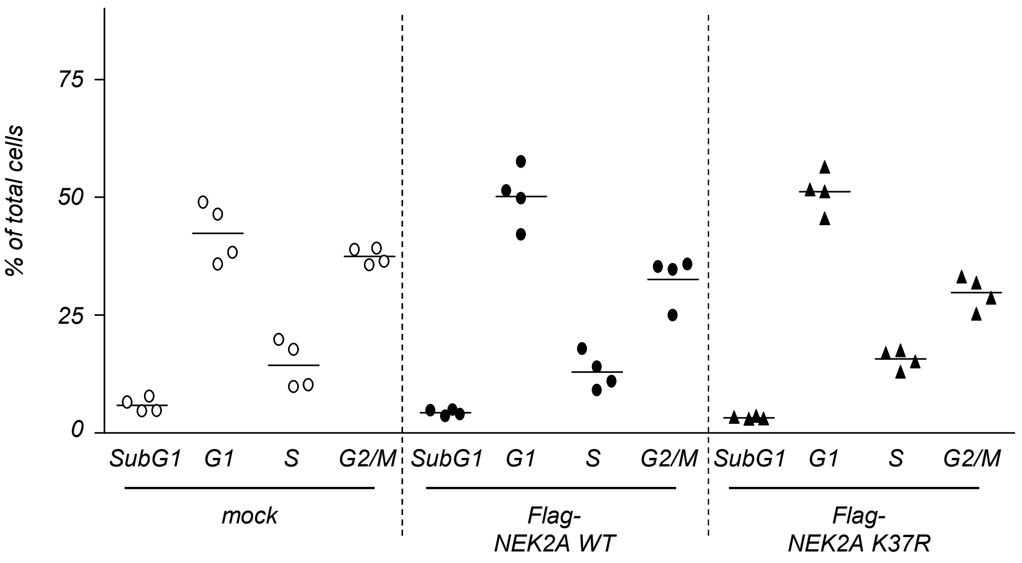

B

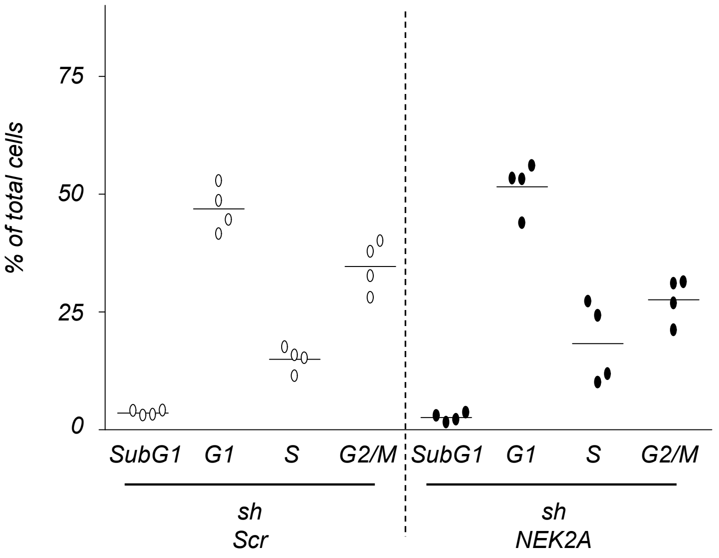

Figure S2. Cell cycle distribution of NEK2A-overexpressing and NEK2A-depleted cells

(A) HuH7 cells overexpressing either mock or Flag-NEK2A WT were incubated with 0.5 mM oleic acid (OA).

(B) HuH7 cells treated with scrambled shRNA (sh Scr) or shRNA against NEK2A (sh NEK2A) were incubated with 0.5 mM OA.

Cells were harvested and analyzed using a Tali Image-Based Cytometer. The results are shown as scattered dot plots (mean ± SD).

SubG1: Cell population including G0 phase cells and cell debris. G1: G1 phase.

S: S phase. G2/M: G2 and M phases.

Figure S3

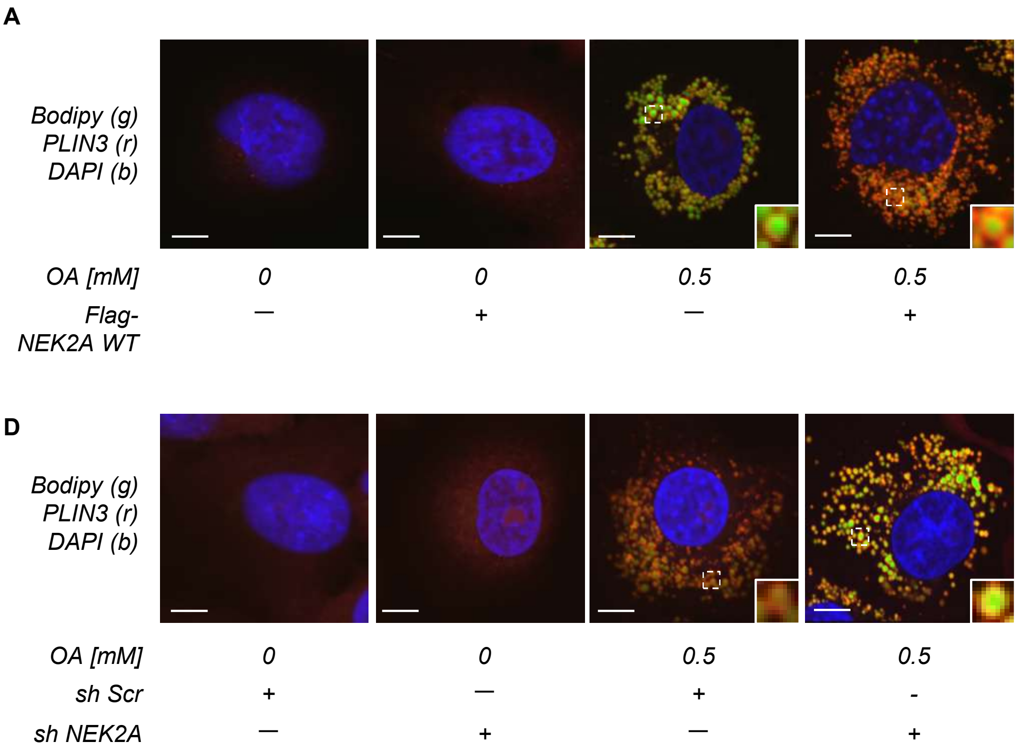

Figure S3. PLIN3 localization in NEK2A-overexpressing and NEK2A-depleted cells

(A) HuH7 cells overexpressing either mock or Flag-NEK2A WT were incubated with or without 0.5 mM oleic acid (OA).

(B) HuH7 cells treated with scrambled shRNA (sh Scr) or shRNA against NEK2A (sh NEK2A) were incubated with or without 0.5 mM OA.

The cells were stained with Bodipy (green, g), PLIN3 (red, r), and DAPI (blue, b). Scale bar: 10 μm.

Figure S4

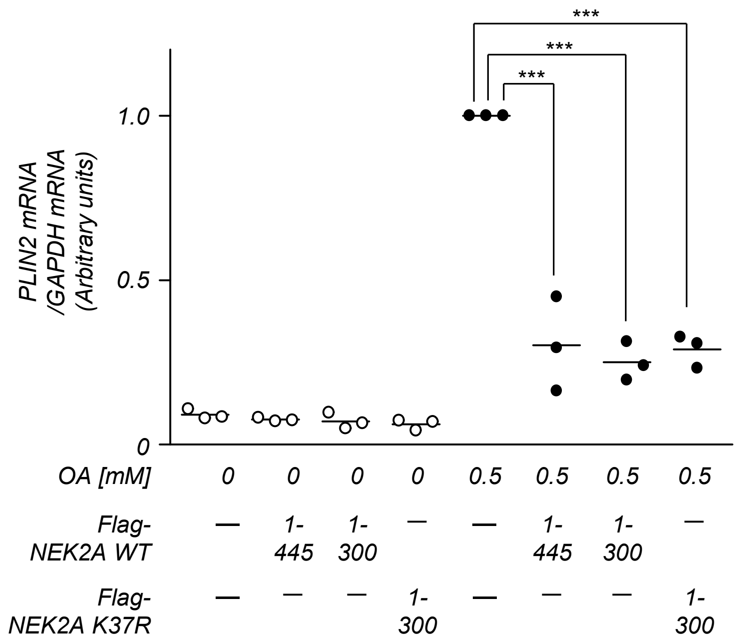

Figure S4. Effect of NEK2A truncation mutants on *PLIN2* mRNA expression

HuH7 cells overexpressing mock, Flag-NEK2A WT (1–445 aa), Flag-NEK2A WT (1–300 aa), or Flag-NEK2A K37R (1–300 aa) were incubated with or without 0.5 mM oleic acid (OA). Total RNA was isolated, and RT-PCR was performed to determine the mRNA levels of PLIN2 normalized to GAPDH. The expression level in OA-treated mock-overexpressing cells was set to 1.0. The results are shown as scattered dot plots (mean ± SD).

$^{***}p<0.001$, one-way ANOVA followed by Tukey–Kramer post hoc test.

Figure S5

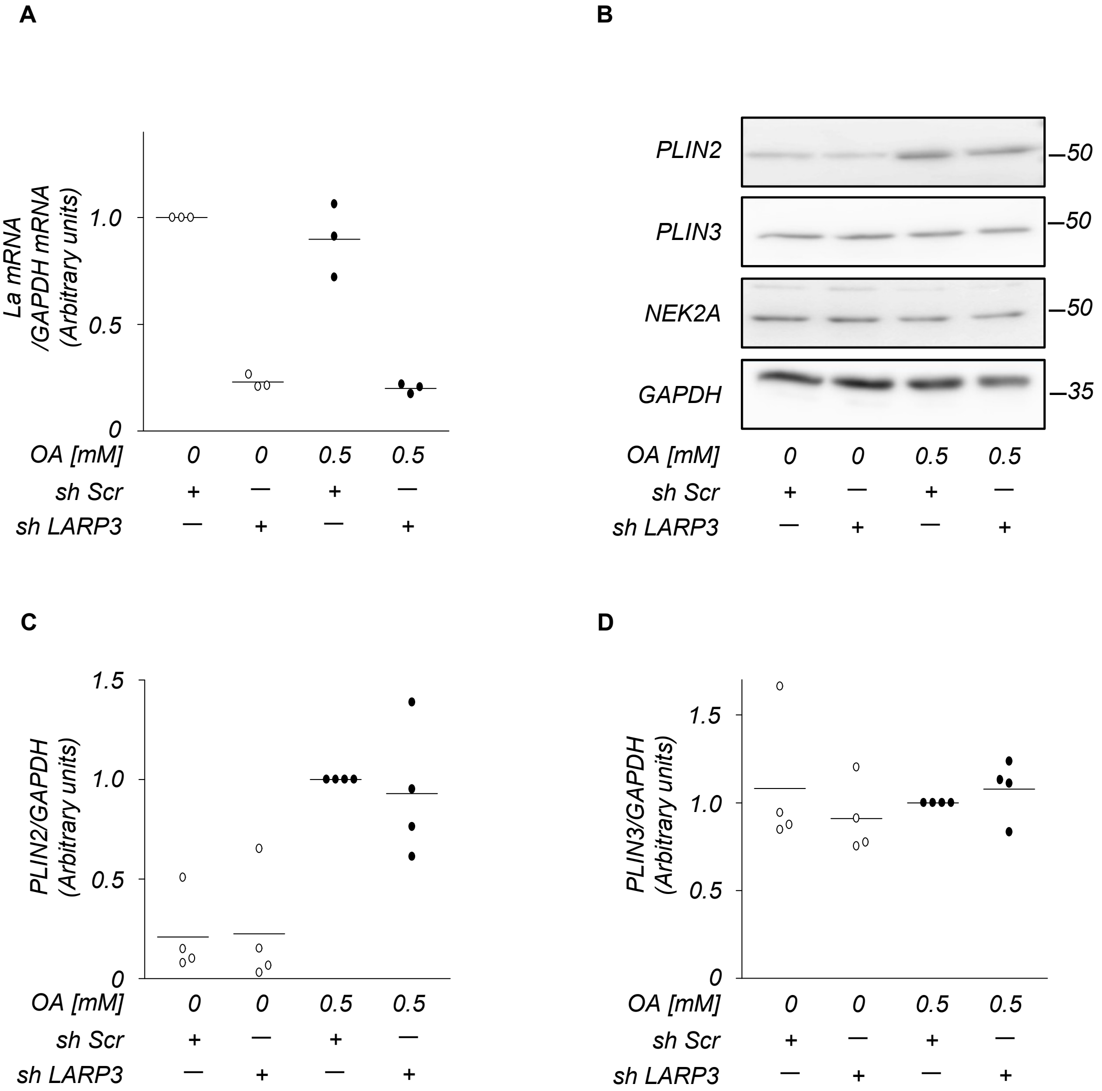

Figure S5. Effect of LARP3 knockdown on PLIN2 and PLIN3 expression

HuH7 cells treated with scrambled shRNA (sh Scr) or shRNA against LARP3 (sh LARP3) were incubated with or without 0.5 mM oleic acid (OA).

(A) Total RNA was isolated, and RT-PCR was performed to determine the mRNA levels of LARP3 normalized to GAPDH. The expression level in sh Scr-treated cells with OA was set to 1.0.

(B) Cell lysates were analyzed by immunoblotting using antibodies against PLIN2, PLIN3, NEK2A, and glyceraldehyde 3-phosphate dehydrogenase (GAPDH). Data shown are representative of three independent experiments.

(C, D) The relative protein levels of PLIN2 (C) and PLIN3 (D) were quantified, normalized to those of GAPDH, and expressed in arbitrary units. PLIN2 and PLIN3 levels in OA-treated sh Scr-treated cells during interphase were set to 1.0. The results are shown as scattered dot plots (mean ± SD).

(E) Quantification of the intracellular TG levels. The results are shown as scattered dot plots (mean ± SD).

Figure S6

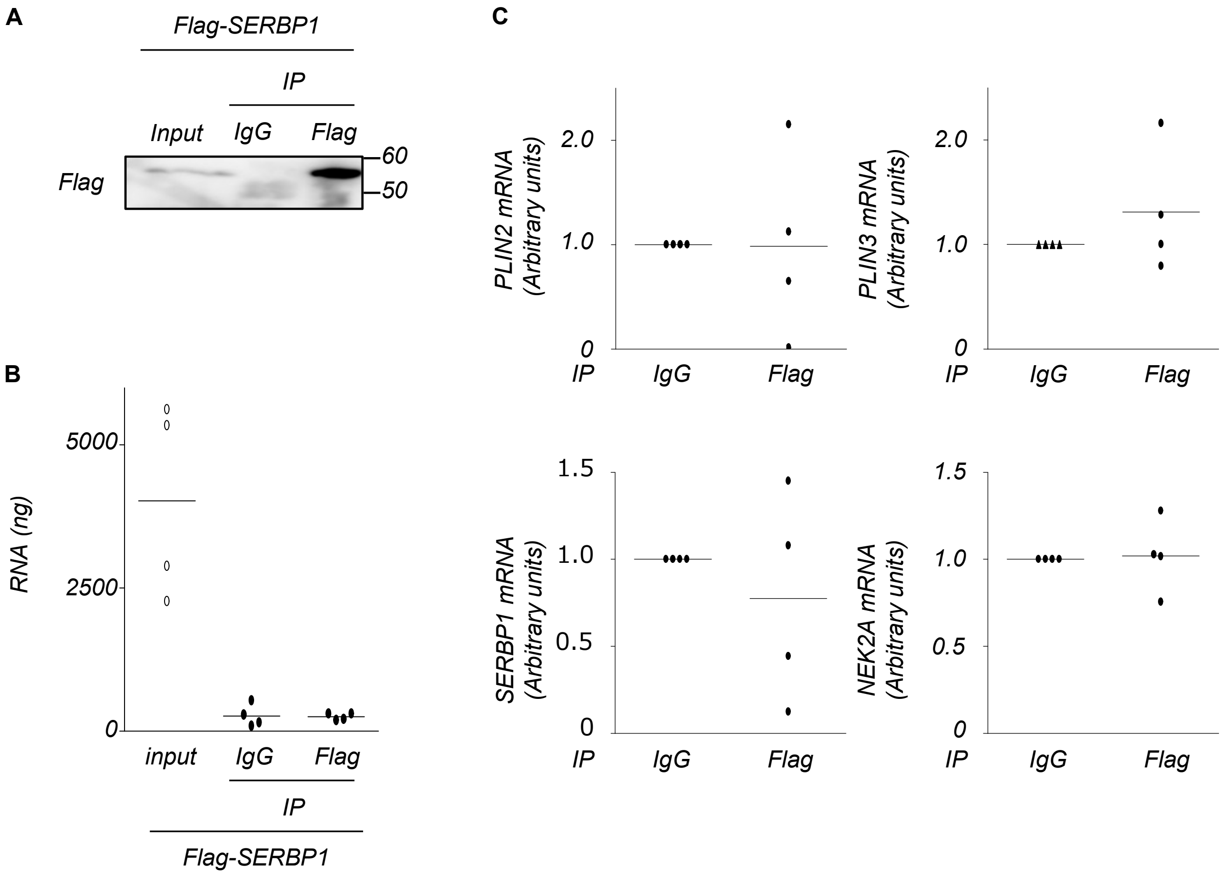

Figure S6. No interaction between SERBP1 protein and PLIN2 mRNA by RNA immunoprecipitation

HuH7 cells expressing 3'-Flag-SERBP1 were incubated with 0.5 mM oleic acid (OA).

(A) Cell lysates were immunoprecipitated with control IgG- or Flag-tag antibodies. Immunoprecipitants were analyzed by immunoblotting using antibodies against Flag. IP: immunoprecipitation.

(B) Total RNA was isolated and quantified using NanoDrop 2000 spectrophotometer.

(C) Total RNA was isolated, and RT-PCR was performed to determine mRNA levels. The expression levels of IgG immunoprecipitants were set to 1.0. The results are shown as scattered dot plots (mean ± SD).

Figure S7

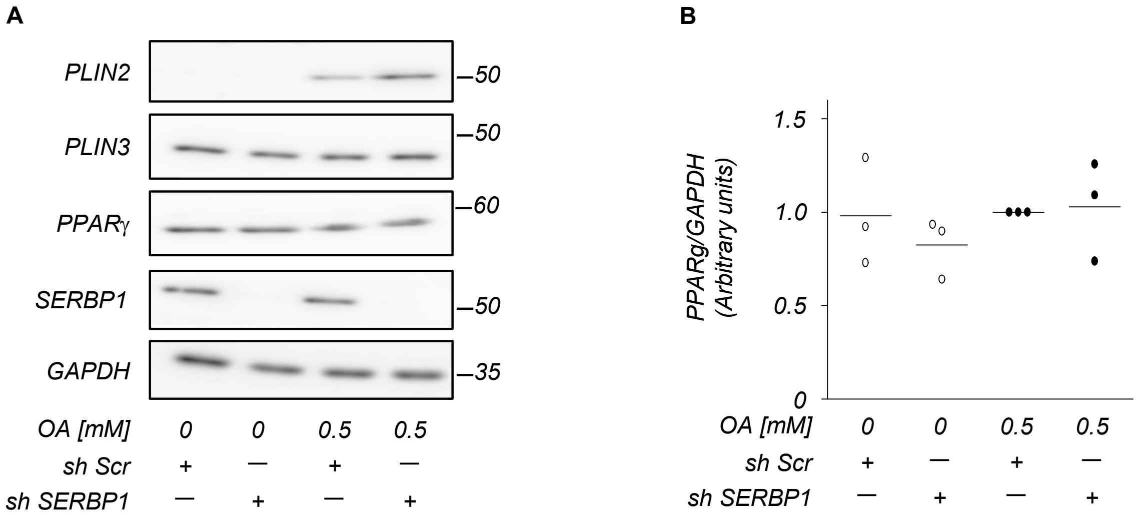

Figure S7. Unchanged total PPARγ expression upon SERBP1 knockdown

(A, B) HuH7 cells were treated with sh scrambled short hairpin RNA (sh Scr) or shRNA against SERBP1 (sh SERBP1). The cells were incubated with or without 0.5 mM oleic acid (OA). (A) Cell lysates were analyzed by immunoblotting using antibodies against PLIN2, PLIN3, PPARγ, SERBP1, and glyceraldehyde 3-phosphate dehydrogenase (GAPDH). Data shown are representative of three independent experiments. (B) The relative protein levels of PPARγ were quantified, normalized to those of GAPDH, and expressed in arbitrary units. PPARγ levels in OA-treated sh Scr-treated cells during interphase were set to 1.0. The results are shown as scattered dot plots (mean ± SD).

Figure S8

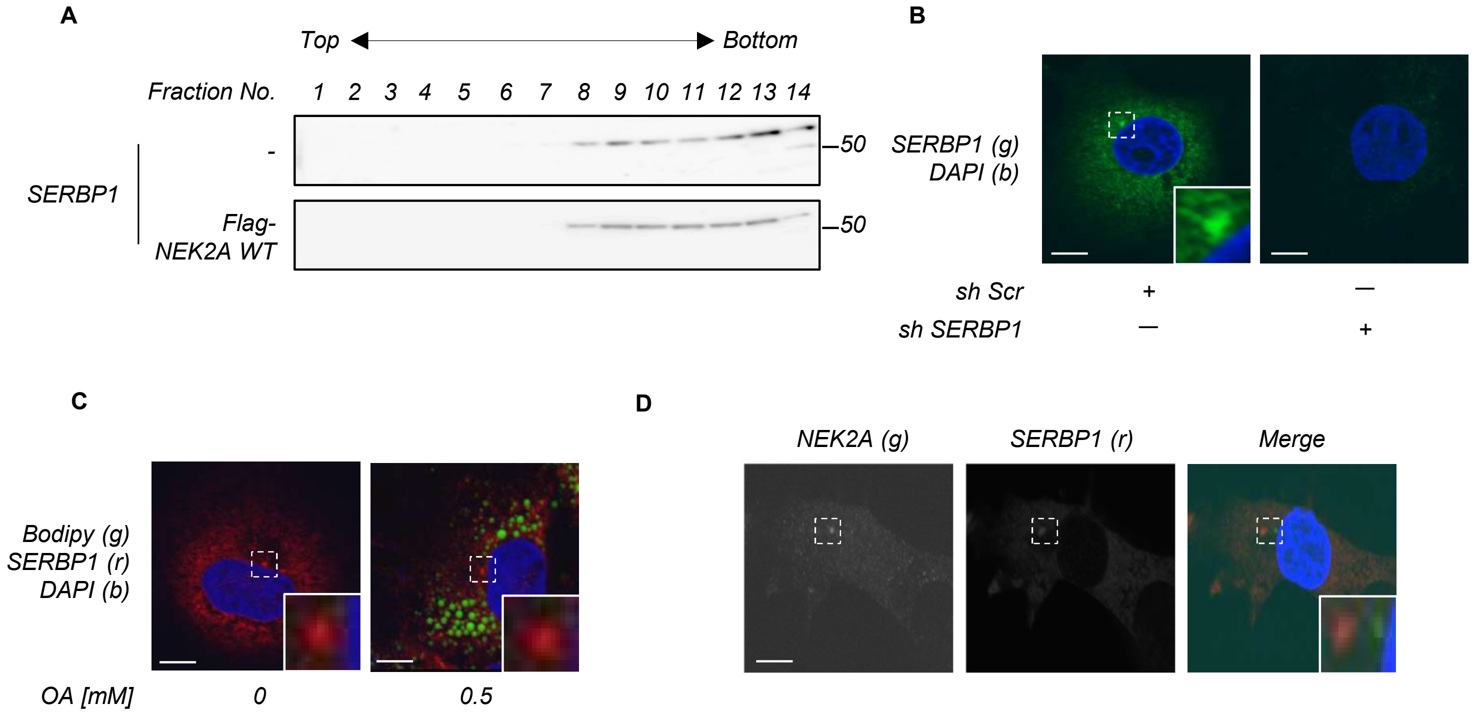

Figure S8. Analysis of SERBP1 localization and expression

(A) HuH7 cells overexpressing mock or Flag-NEK2A WT were incubated with 0.5 mM oleic acid (OA). Lysates from cells containing LDs were fractionated by sucrose density gradient centrifugation. An aliquot of each fraction was analyzed by immunoblotting using antibodies against SERBP1.

(B) HuH7 cells treated with scrambled shRNA (sh Scr) or shRNA against SERBP1 (sh SERBP1) were stained for SERBP1 (green, g) and DAPI (blue, b). Scale bar: 10 μm.

(C) HuH7 cells were incubated with or without 0.5 mM OA. The cells were stained for Bodipy (green, g), SERBP1 (red, r), and DAPI (blue, b). Scale bar: 10 μm.

(D) HuH7 cells were incubated with 0.5 mM OA and stained for NEK2A (green, g), SERBP1 (red, r), and DAPI (blue, b). Scale bar: 10 μm.

Supplementary Table 1. Sequences for shRNA

ShRNA	*Sequences (5'-3')*
Human NEK2A	*sense: GGATTATTGACCGGACCAATA*
Human SERBP1	*sense: GCATAGTGGAAGTGATAGA*
Human AURKA	*sense: AATGCCCTGTCTTACTGTCATTC*
Human LARP3	*sense: GCAACTCTTGATGACATAA*

AURKA, the gene for Aurora A kinase; LARP3, La-related protein 3

Supplementary Table 2. Antibodies used in this study

Antibody	*Source*	*Dilution*	*Company (#Catalogue)*
Aurora A	*rabbit*	*1:500 (IB)*	*CST (4718T)*
Flag	*rabbit*	*1:1000 (IB)*	*CST (14793S)*
Flag	*mouse*	*2 μg (IP)*	*Sigma (F3165)*
GAPDH	*rabbit*	*1:5000 (IB)*	*Sigma (G9545)*
HRP-rabbit	*donkey*	*1:5000 (IB)*	*GE healthcare (NA934)*
HRP-mouse	*sheep*	*1:2000 (IB)*	*GE healthcare (NA931)*
IgG	*mouse*	*2 μg (IP)*	*Sigma (I5381)*
LC3	*rabbit*	*1:1000 (IB)*	*Protein tech (14600-1-AP)*
NEK2A	*mouse*	*1:500 (IB)*	*BD bioscience (610593)*
PPARγ	*rabbit*	*1:1000 (IB)*	*Protein tech (16643-1-AP)*
PLIN2	*rabbit*	*1:1000 (IB)*<br>*1:500 (IF)*	*Protein tech (15294-1-AP)*
PLIN3	*rabbit*	*1:2000 (IB)*<br>*1:500 (IF)*	*Protein tech (10694-1-AP)*
SERBP1	*rabbit*	*1:1000 (IB)*	*Protein tech (10729-1-AP)*
	rabbit	*1:500 (IF)*	*Thermo (A303938AT)*

IB: Immunoblotting
IF: Immunofluorescence
IP: Immunoprecipitation

Supplementary Table 3. Dyes for immunofluorescence (IF)

Dye	*Source*	*Dilution*	*Company (#Catalogue)*
Bodipy493/503		*1 μg/ml*	*Sigma (D3922)*
LipidTox Red		*1 μg/ml*	*Invitrogen (H34476)*
DAPI		*1:1000*	*Dojindo (340-07971)*
Alexa488-rabbit	*goat*	*1:500*	*Thermo (A11034)*
Alexa594-rabbit	*goat*	*1:500*	*Thermo (A11037)*
Alexa488-mouse	*donkey*	*1:500*	*Thermo (A21202)*
Alexa594-mouse	*donkey*	*1:500*	*Thermo (A21203)*

Supplementary Table 4. Sequences of the qPCR primers

qPCR primers	*Sequences (5'-3')*
Human CD36 (forward)	*CAGGTCAACCTATTGGTCAAGCC*
Human CD36 (reverse)	*GCCTTCTCATCACCAATGGTCC*
Human GAPDH (forward)	*CCTGTTCGACAGTCAGCCG*
Human GAPDH (reverse)	*CGACCAAATCCGTTGACTCC*
Human PLIN2 (forward)	*TCAGCTCCATTCTACTGTTCACC*
Human PLIN2 (reverse)	*CCTGAATTTTCTGATTGGCACT*
Human PLIN3 (forward)	*AAGCAGAGGGCACAGGAG*
Human PLIN3 (reverse)	*ACGCCTTGCTTGACAGTTTC*
Human NEK2A (forward)	*CATTGGCACAGGCTCCTAC*
Human NEK2A (reverse)	*TGGAGCCATAGTCAAGTTCTTTC*
Human SERBP1 (forward)	*AGAAAGGCGACCACCTCGTGAA*
Human SERBP1 (reverse)	*ACCTCTTCCAAGACCACCACGA*
Human LARP3 (forward)	*GGATAGACTTCGTCAGAGGAGC*
Human LARP3 (reverse)	*TTTTCCACCTCTCCTTCTAGTACT*

Data S1.
Annotated raw uncropped immunoblot images corresponding to the immunoblot figures presented in this study.

Fig1A NEK2A

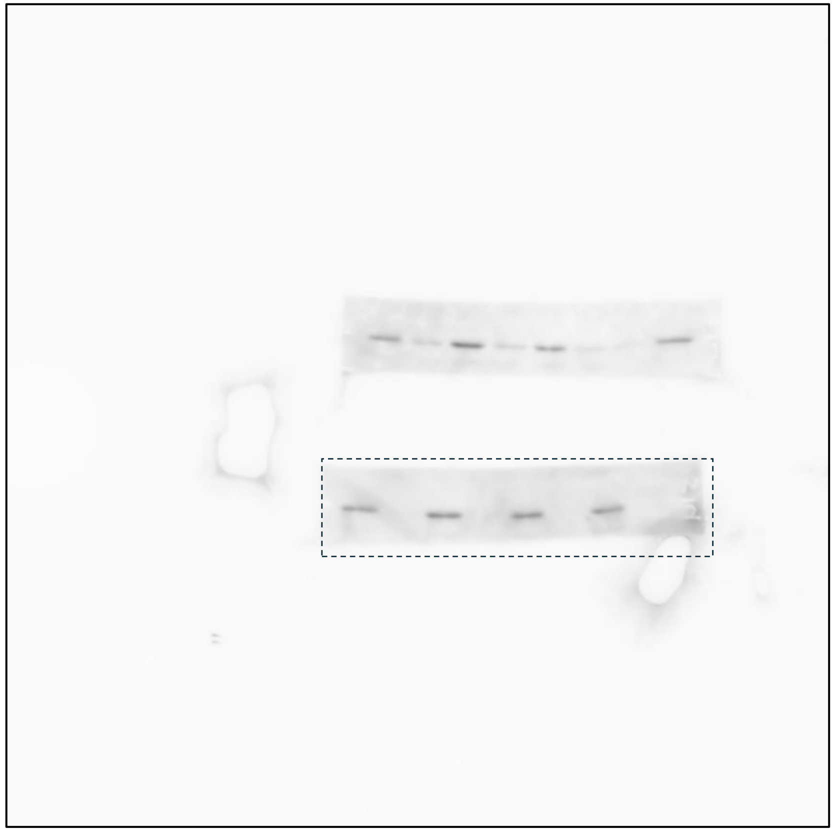

Fig1A PLIN2

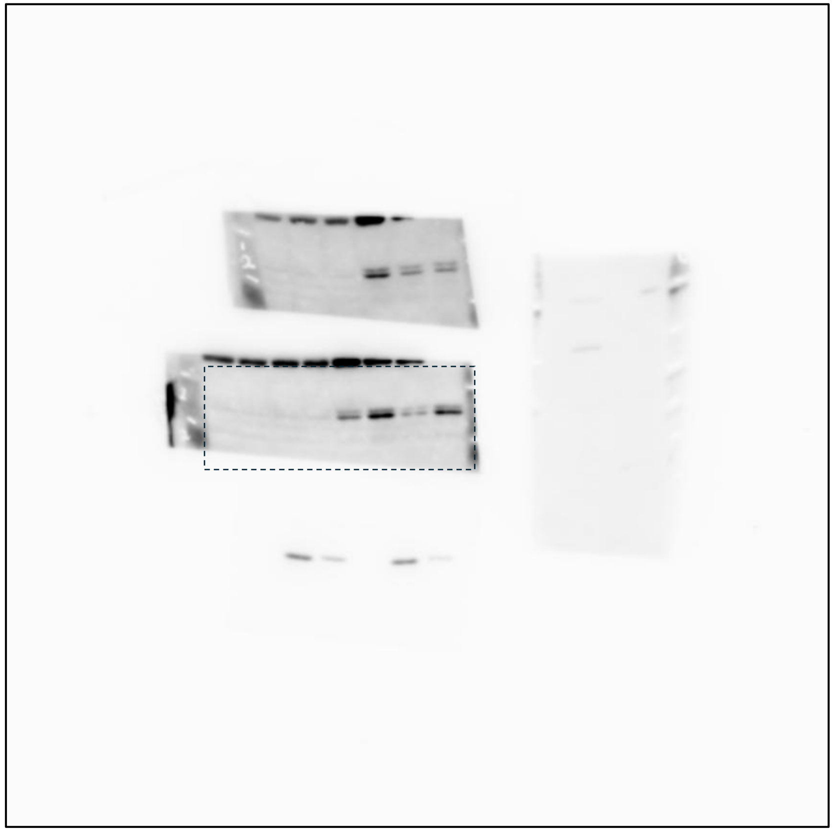

Fig1A GAPDH (upper), PLIN3 (lower)

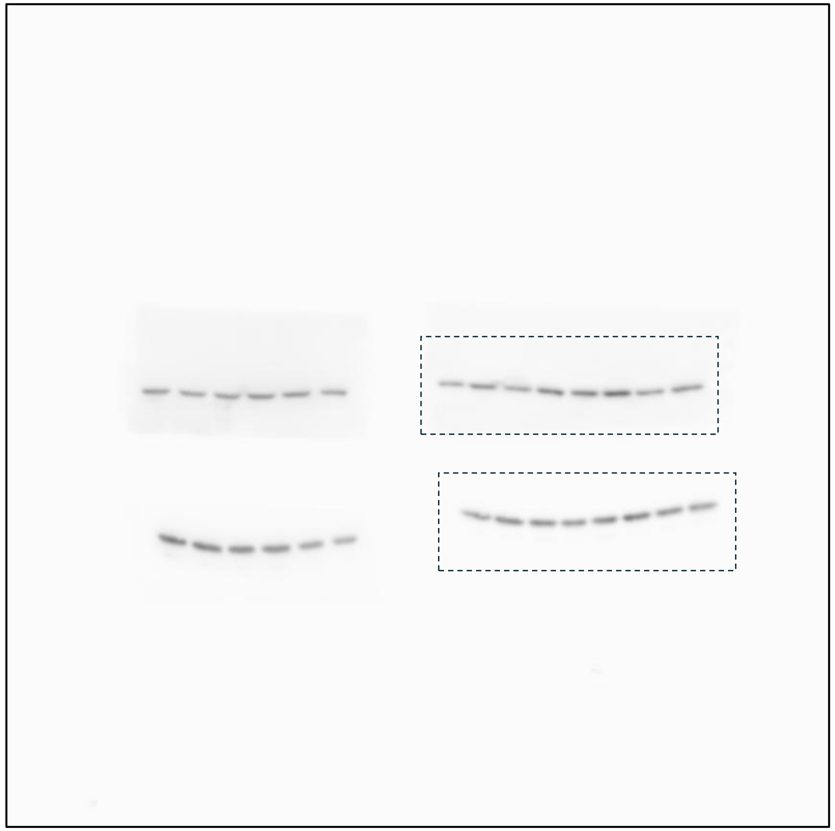

Fig1E Aurora A

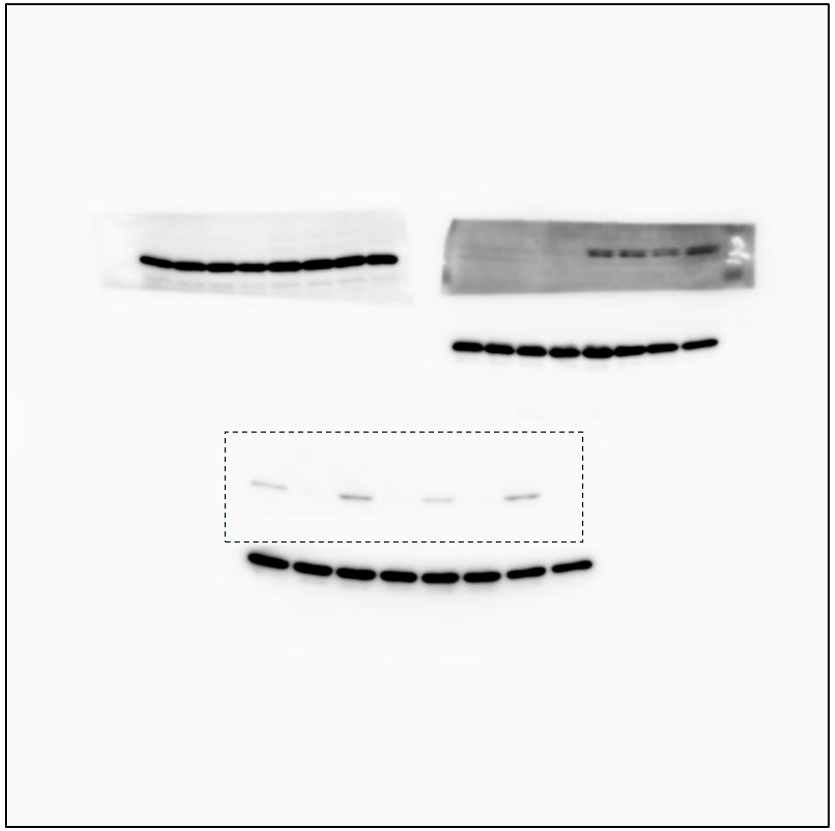

Fig1E GAPDH

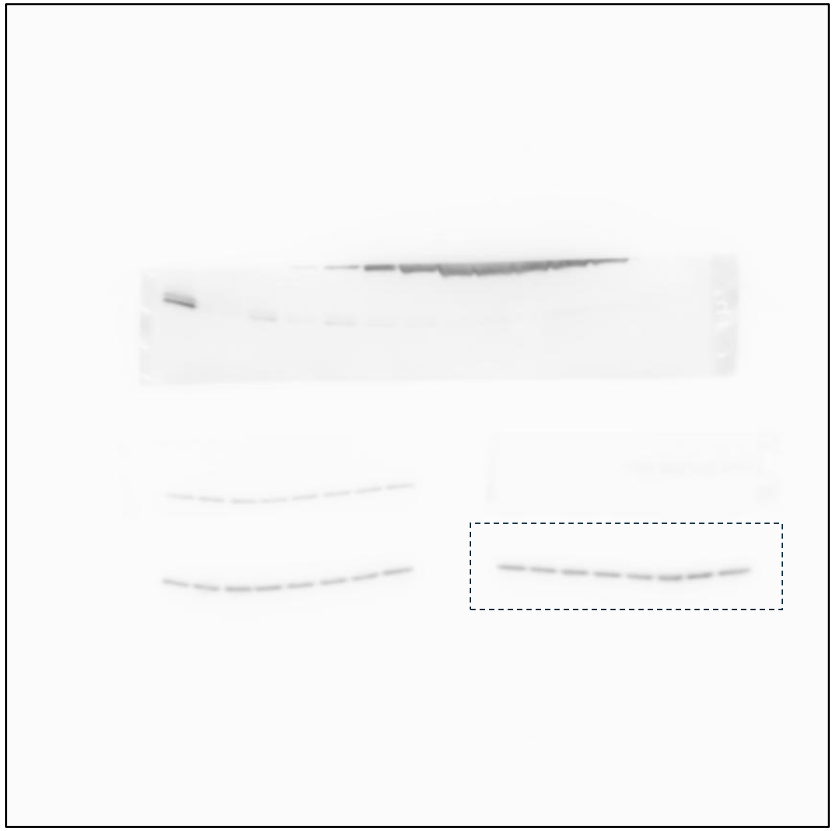

Fig1E PLIN2

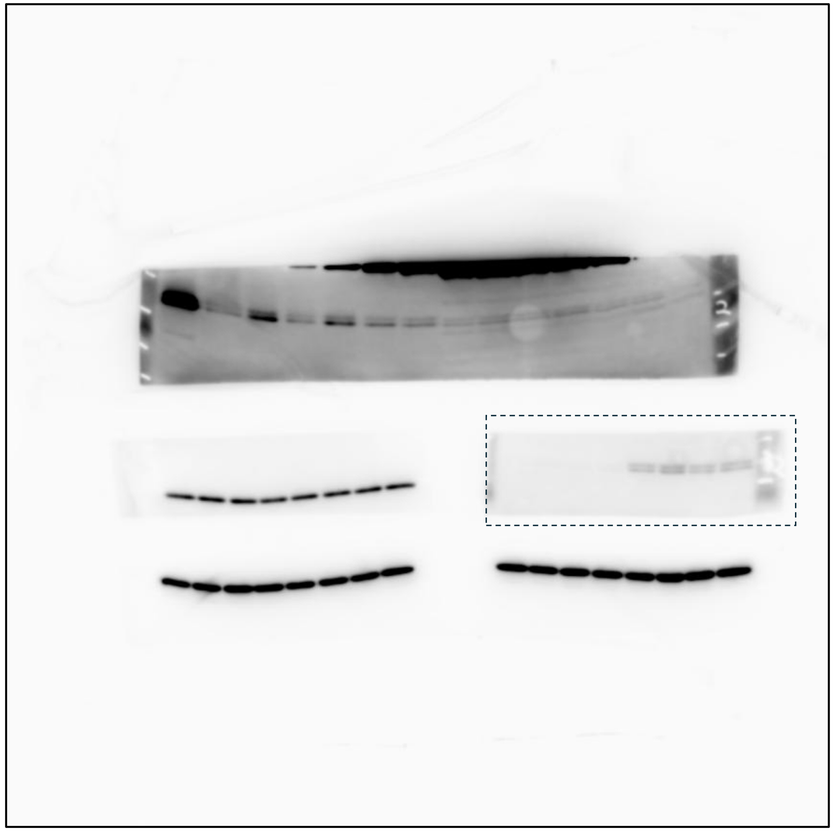

Fig1E PLIN3

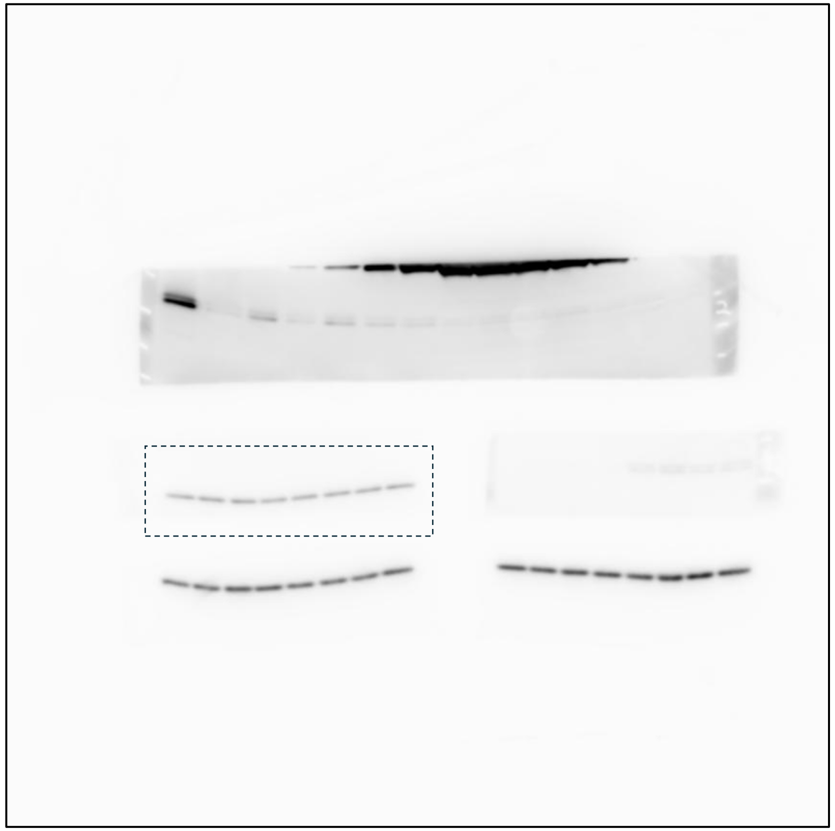

Fig2A Flag

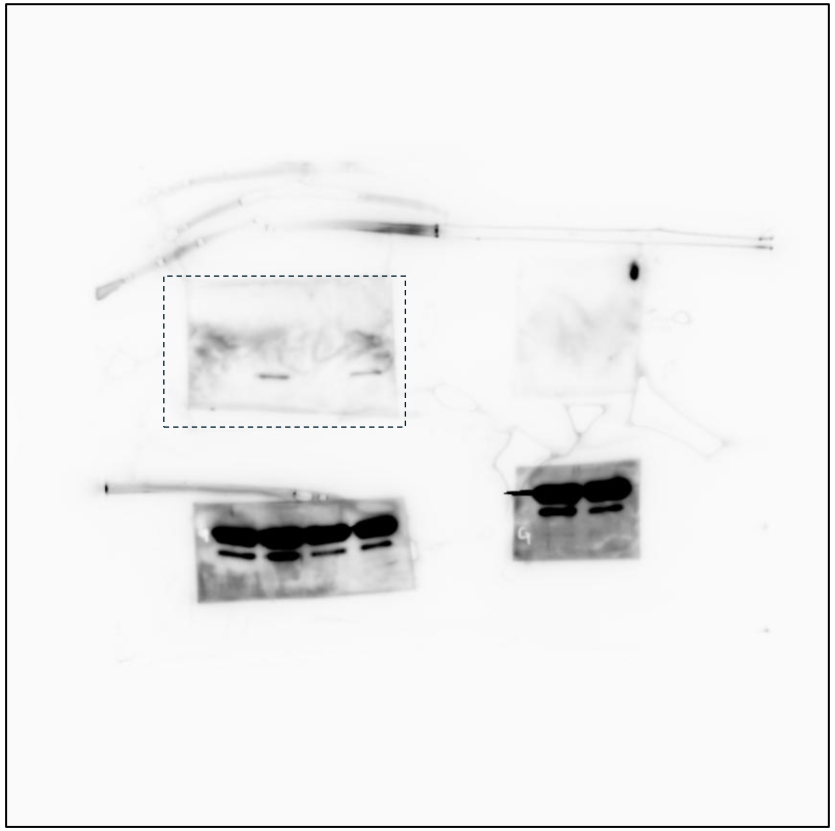

Fig2A PLIN3 (left), GAPDH (right)

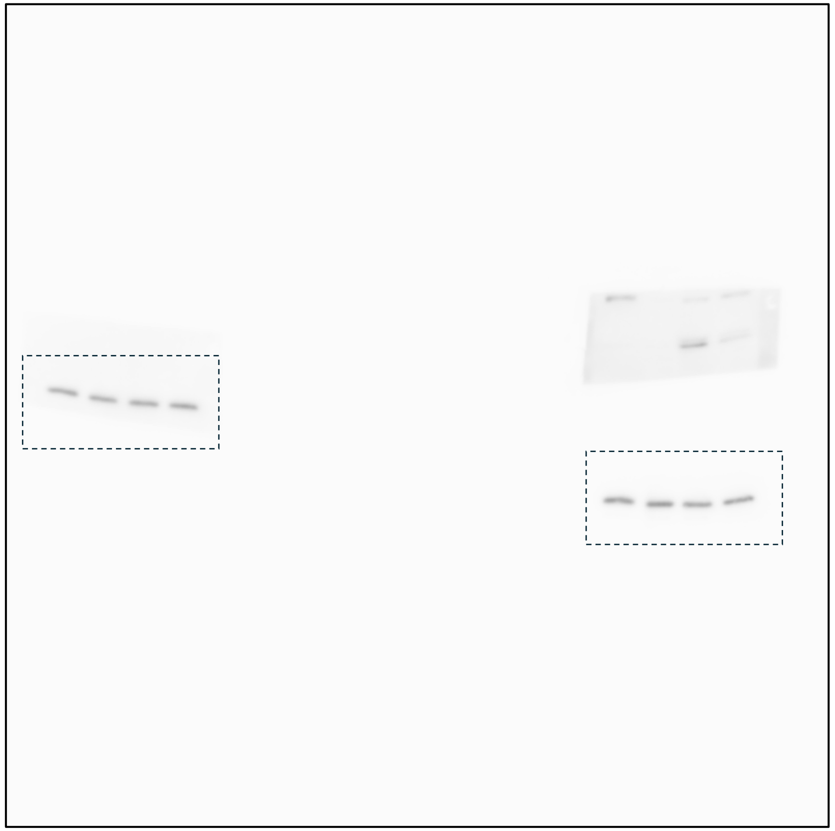

Fig2A NEK2A

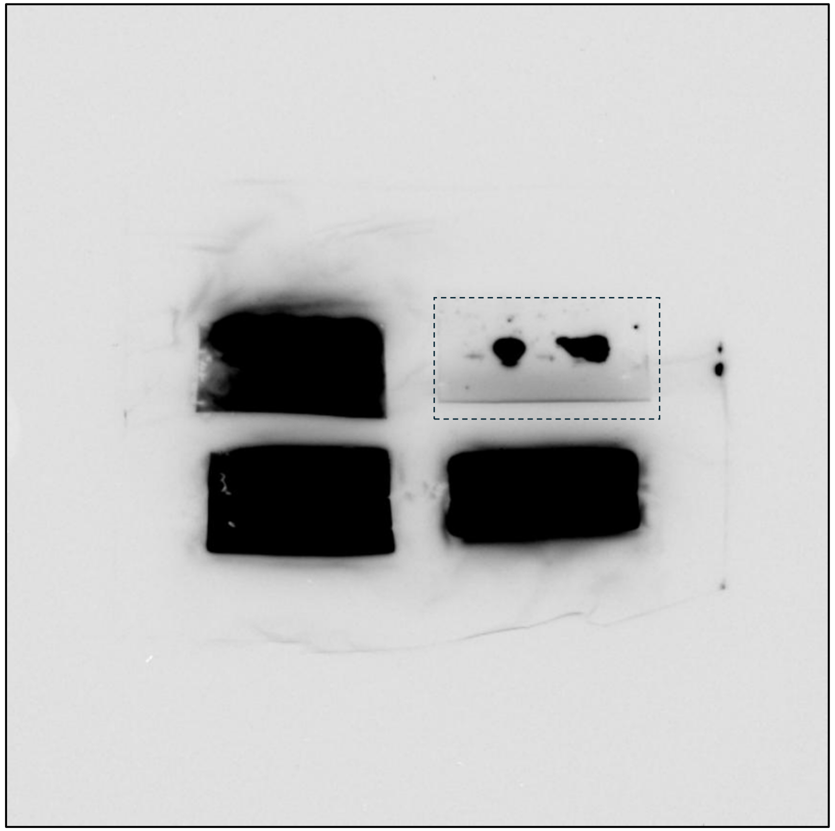

Fig2A PLIN2

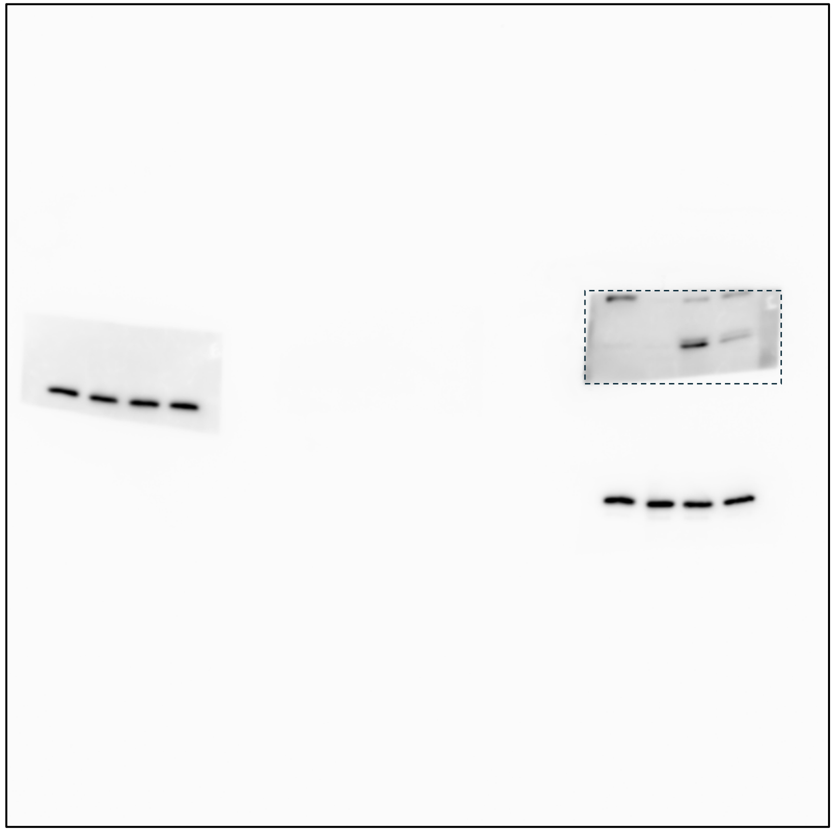

Fig3A PLIN3 (upper), PLIN2 (lower)

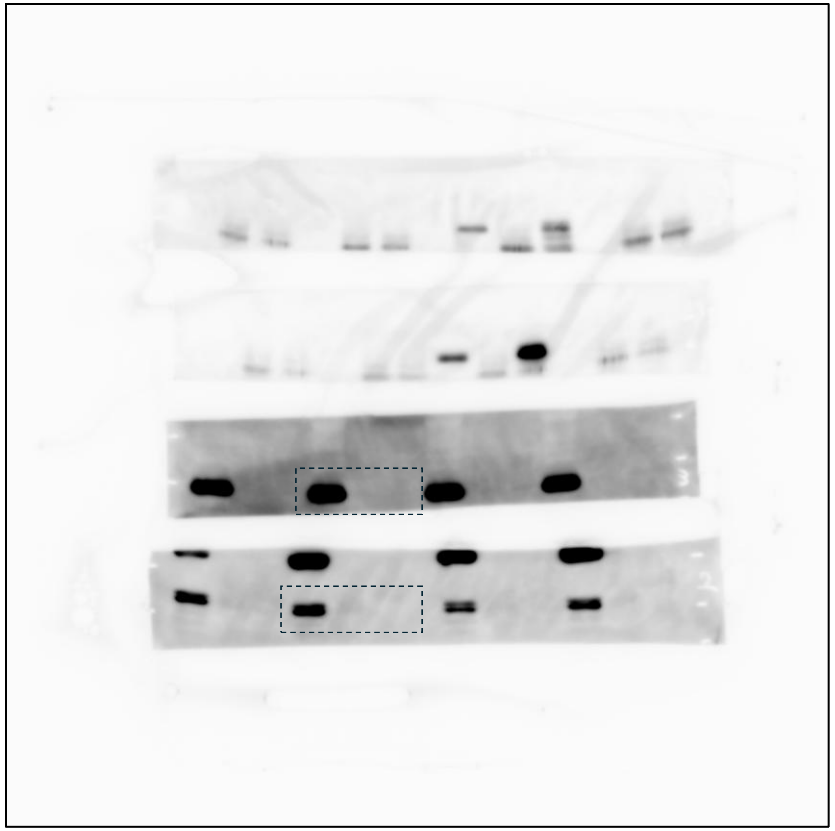

Fig3A Flag

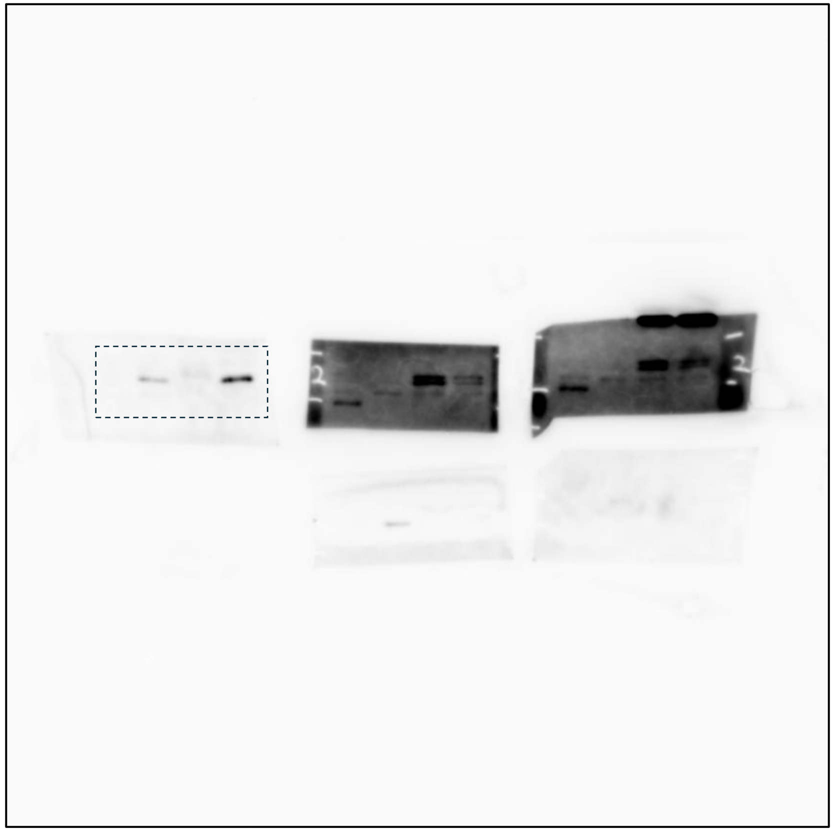

Fig3B None PLIN2

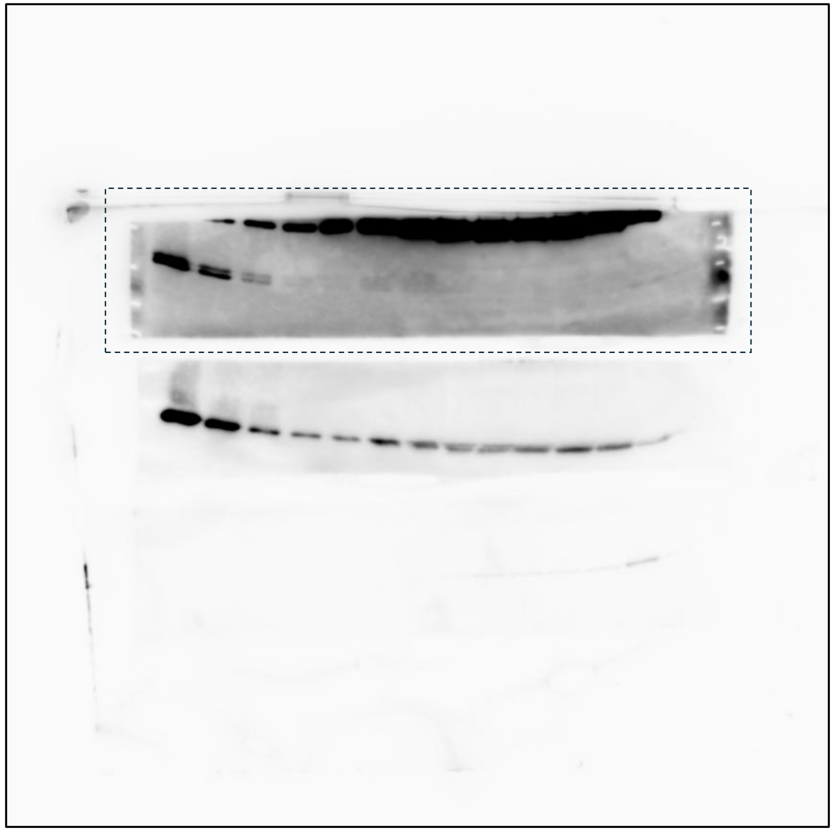

Fig3B None PLIN3

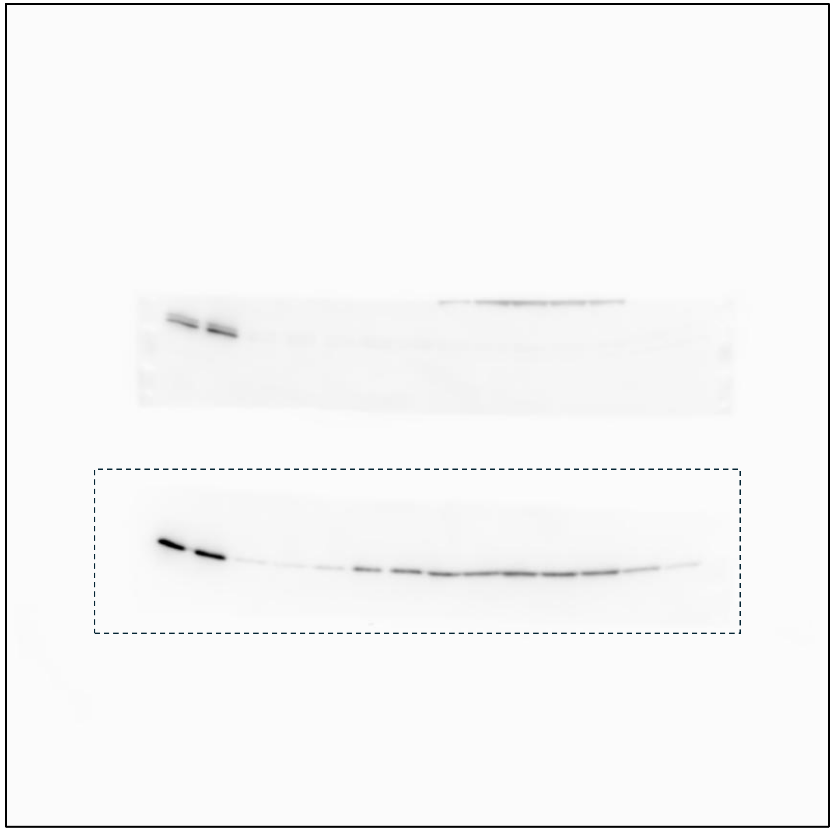

Fig3B WT PLIN2

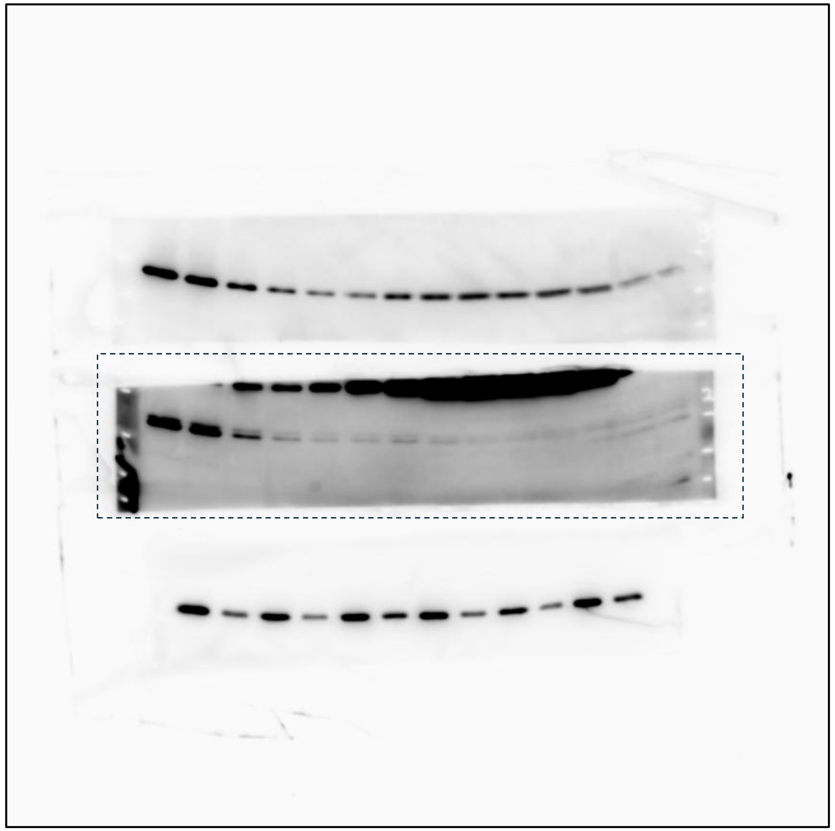

Fig3B WT PLIN3

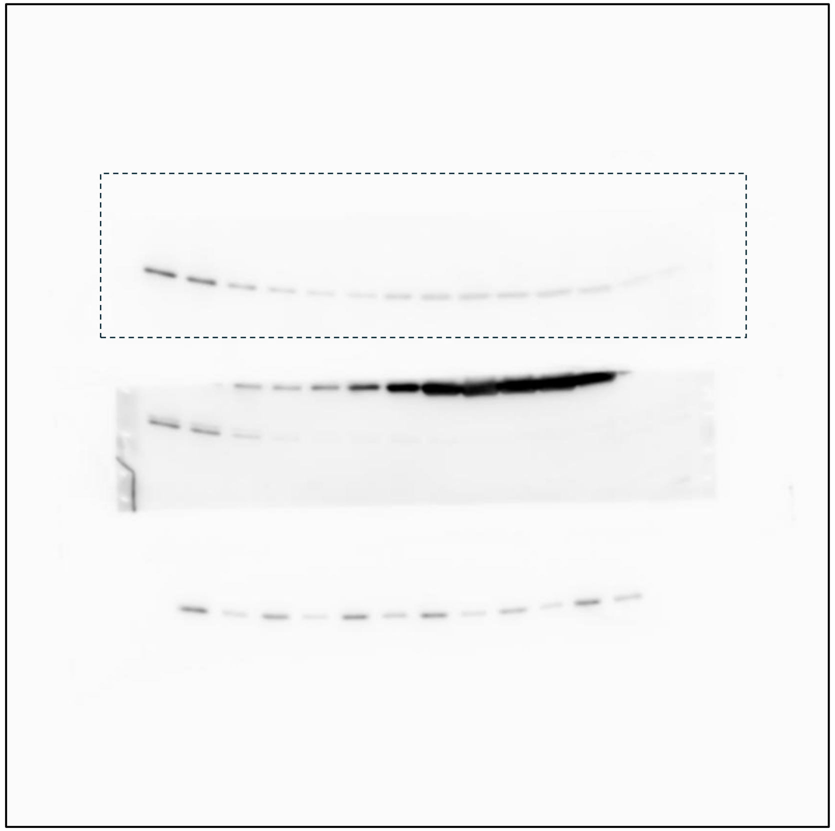

Fig3B WT Flag

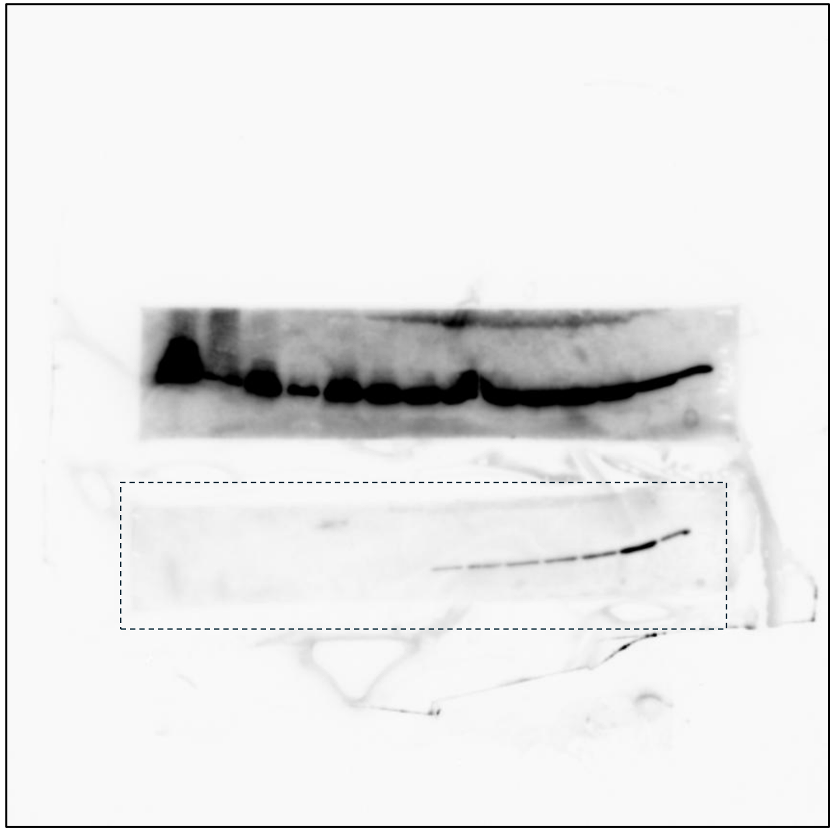

Fig6A Flag

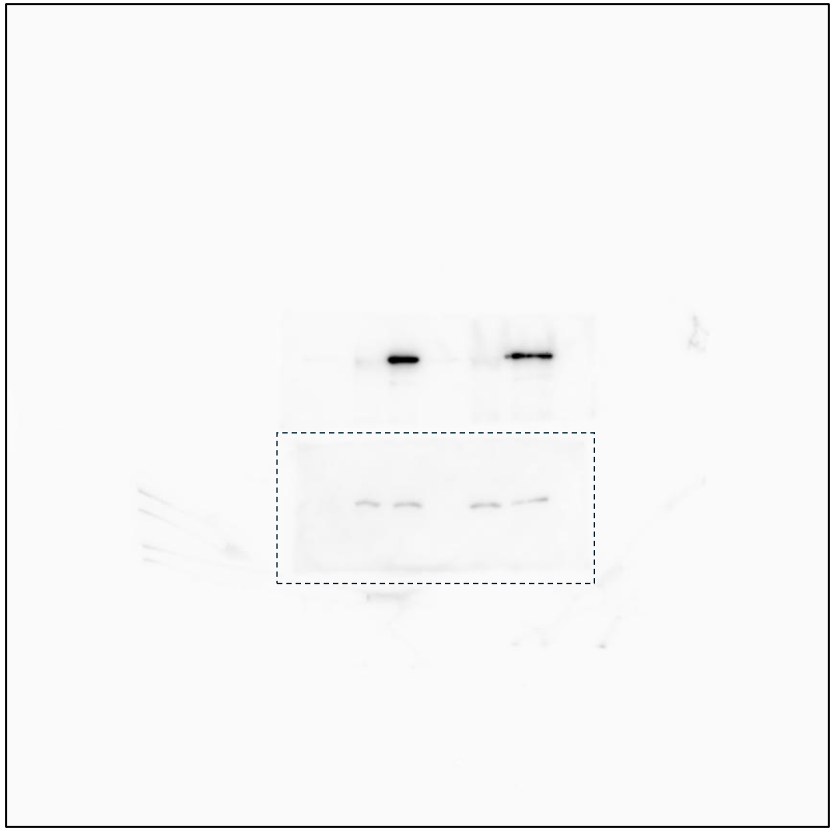

Fig6A GAPDH

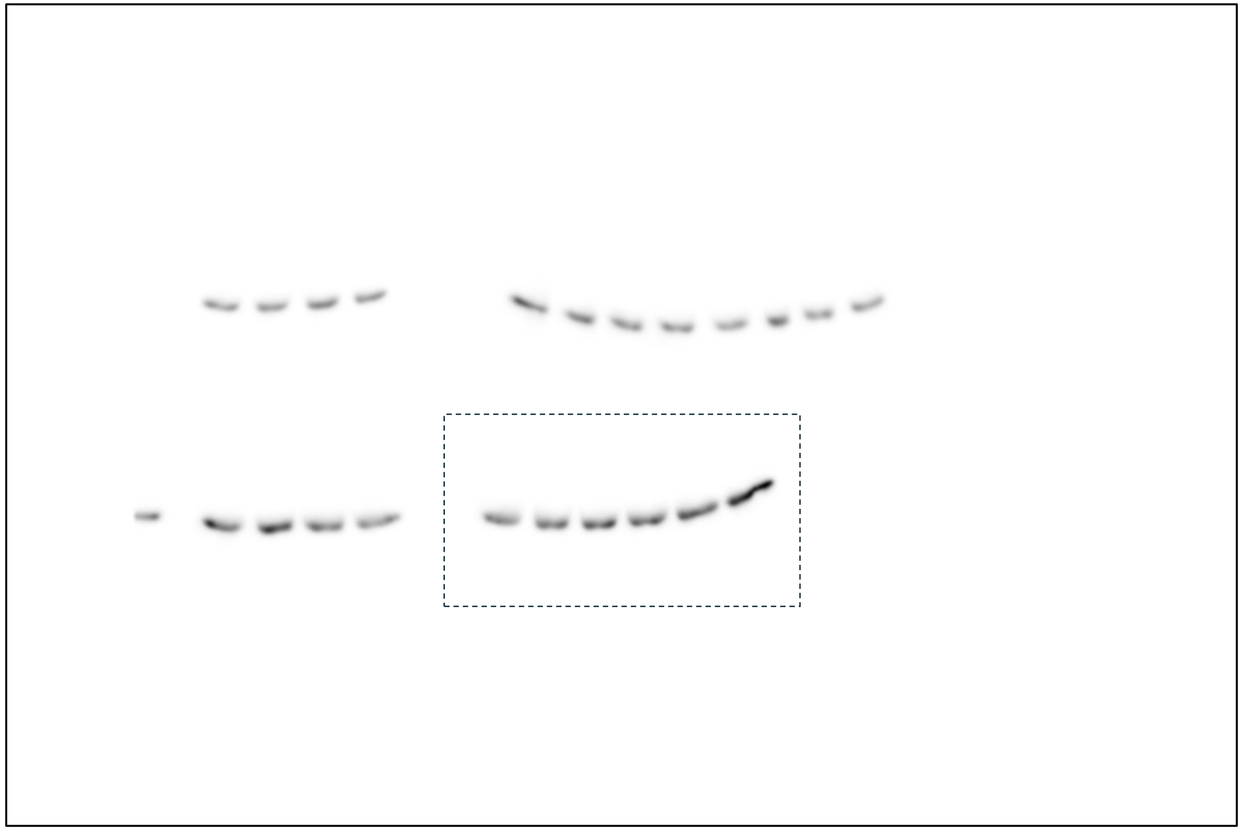

Fig6A NEK2A

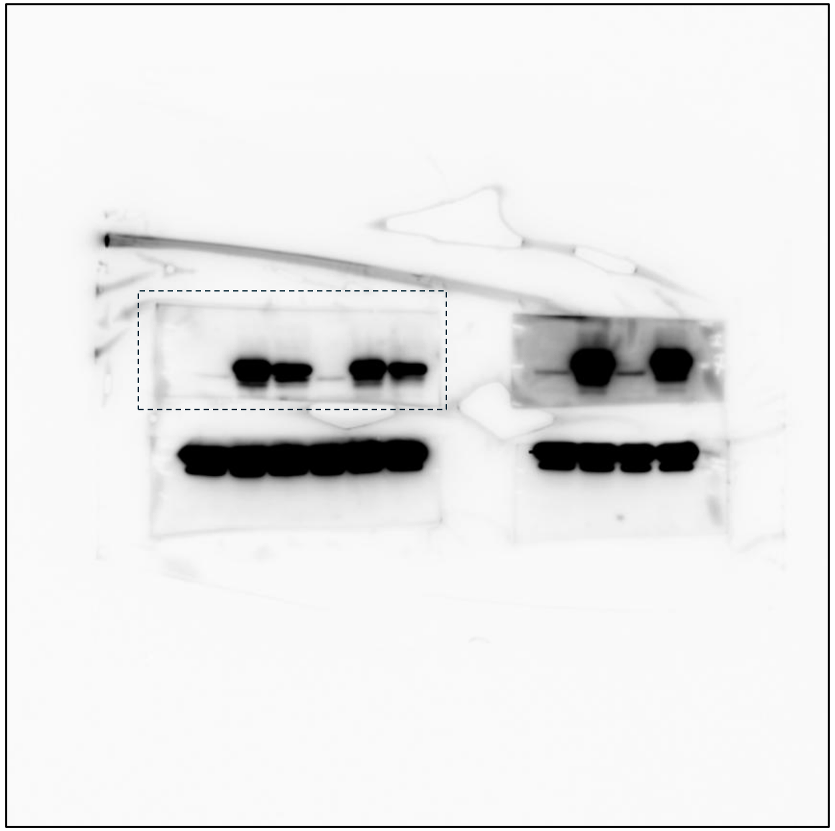

Fig6A PLIN2

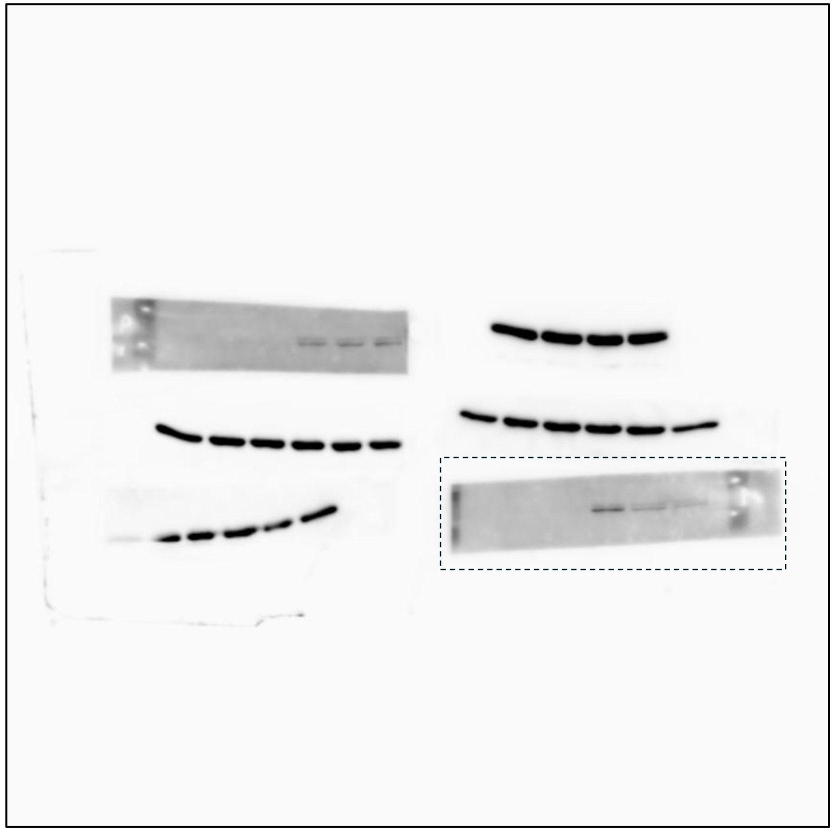

Fig6A PLIN3

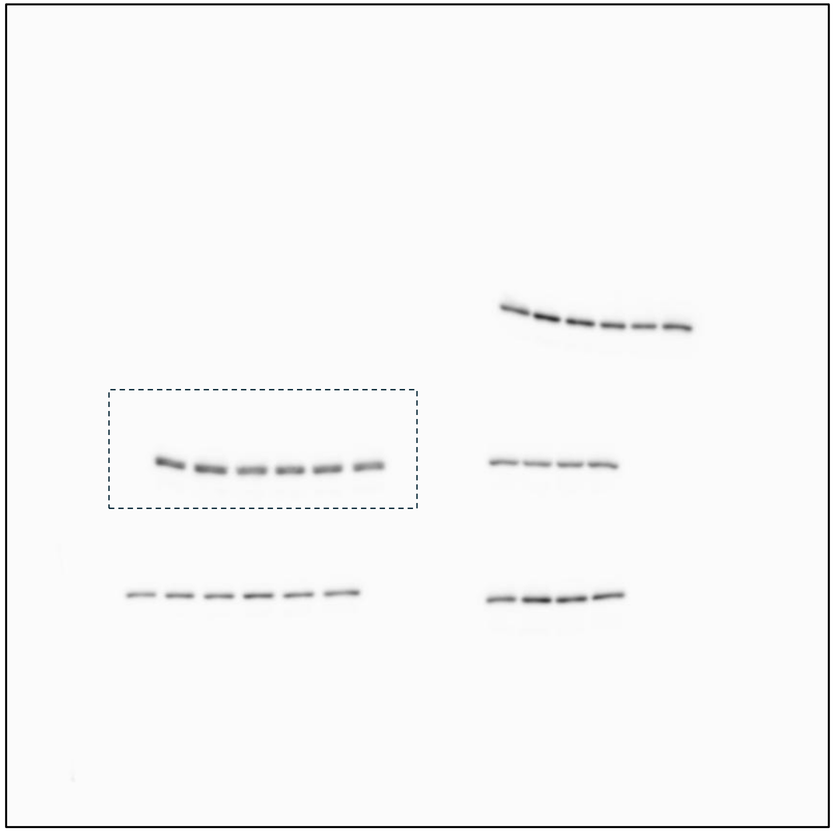

Fig7B Flag

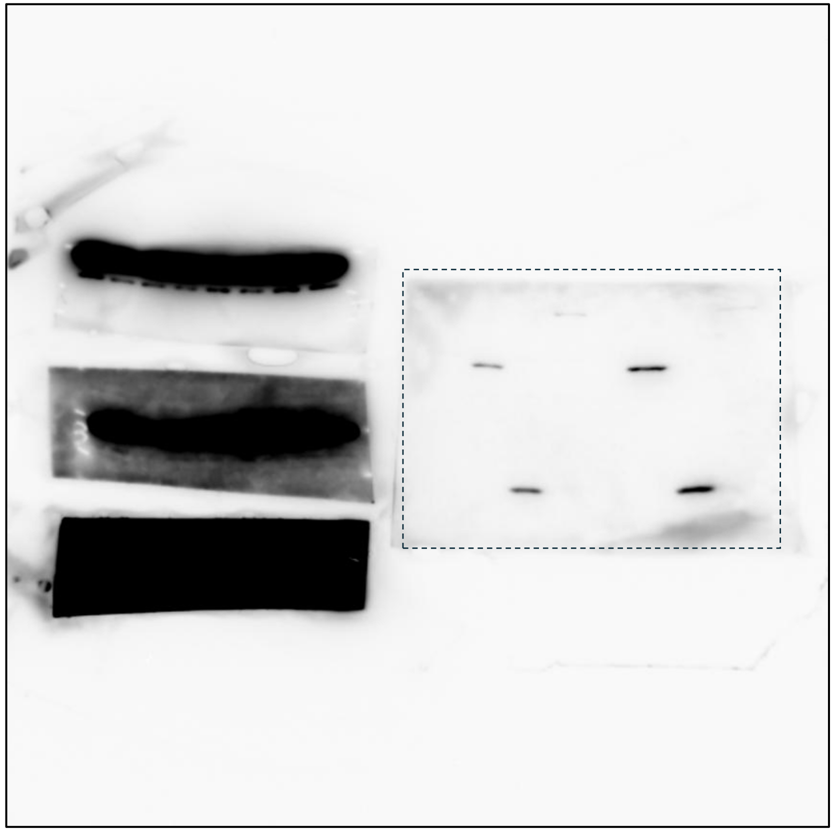

Fig7B GAPDH (upper)

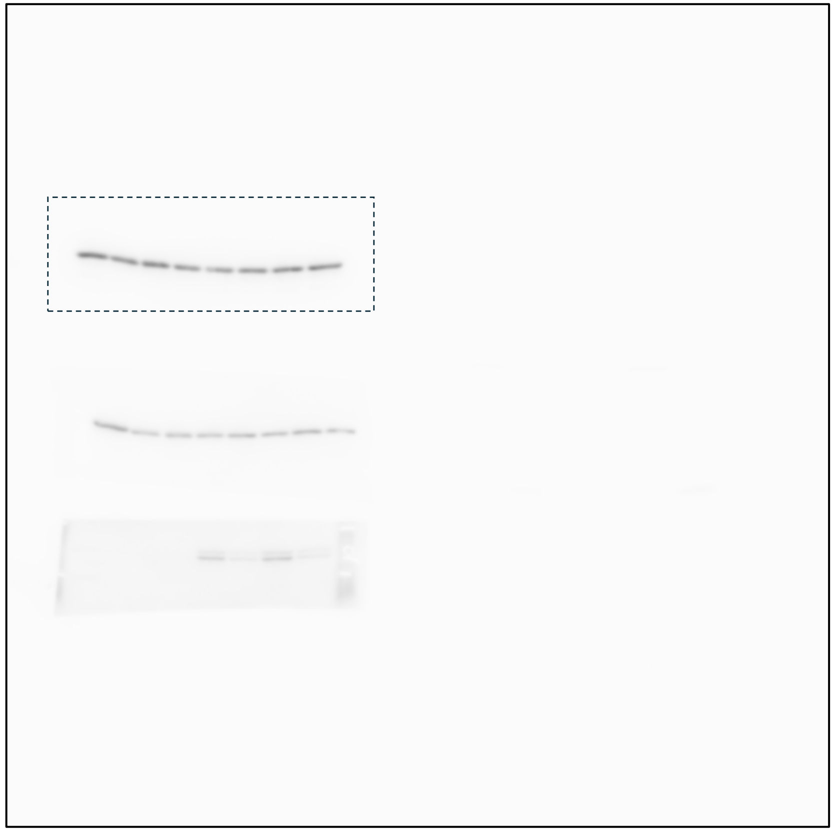

Fig7B PLIN3 (upper), PLIN2 (lower)

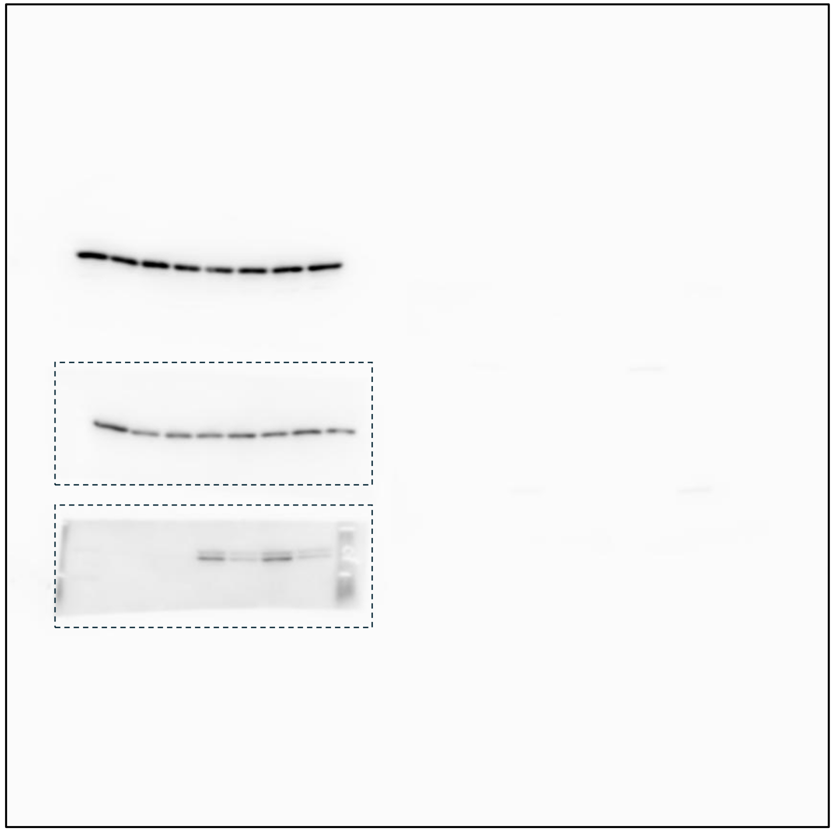

Fig7E PLIN3 (left), GAPDH (right)

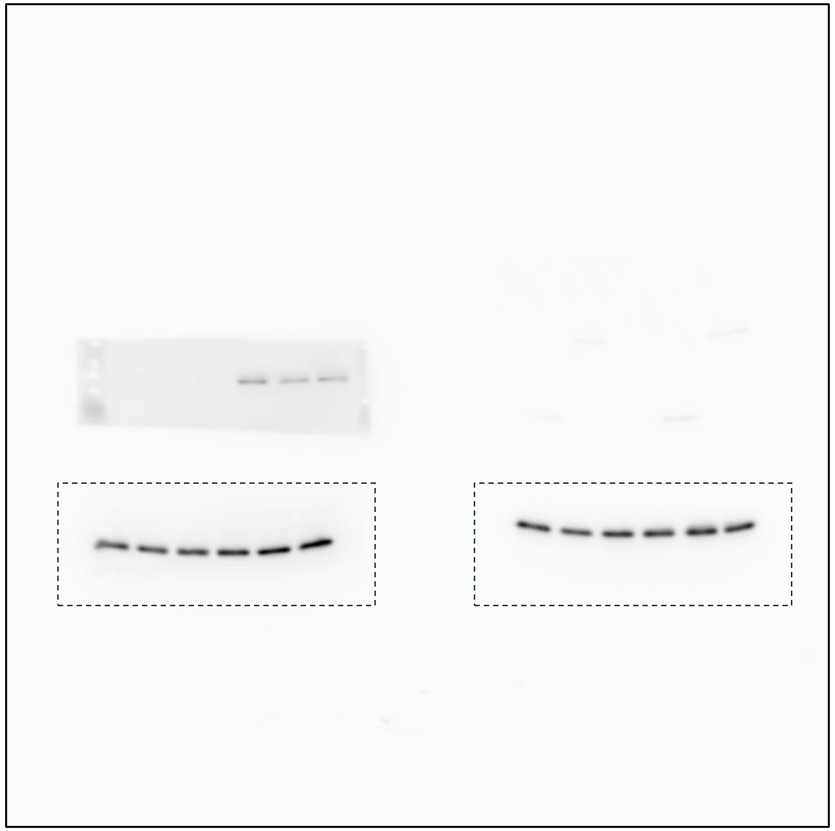

Fig7E PLIN2 (upper)

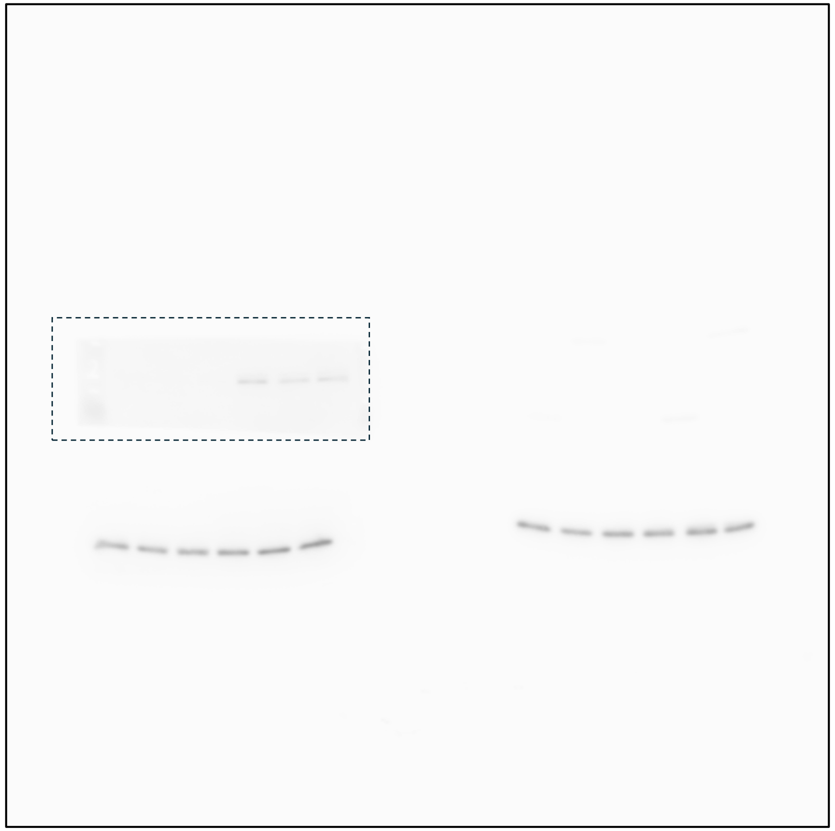

Fig7E Flag

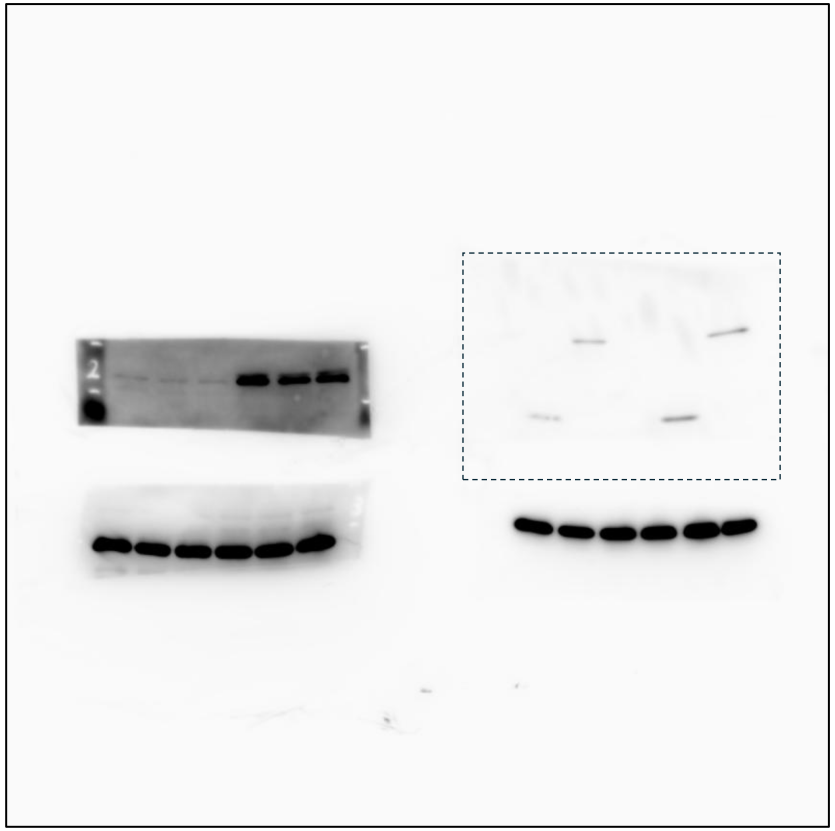

Fig8B WT 1-300 Flag

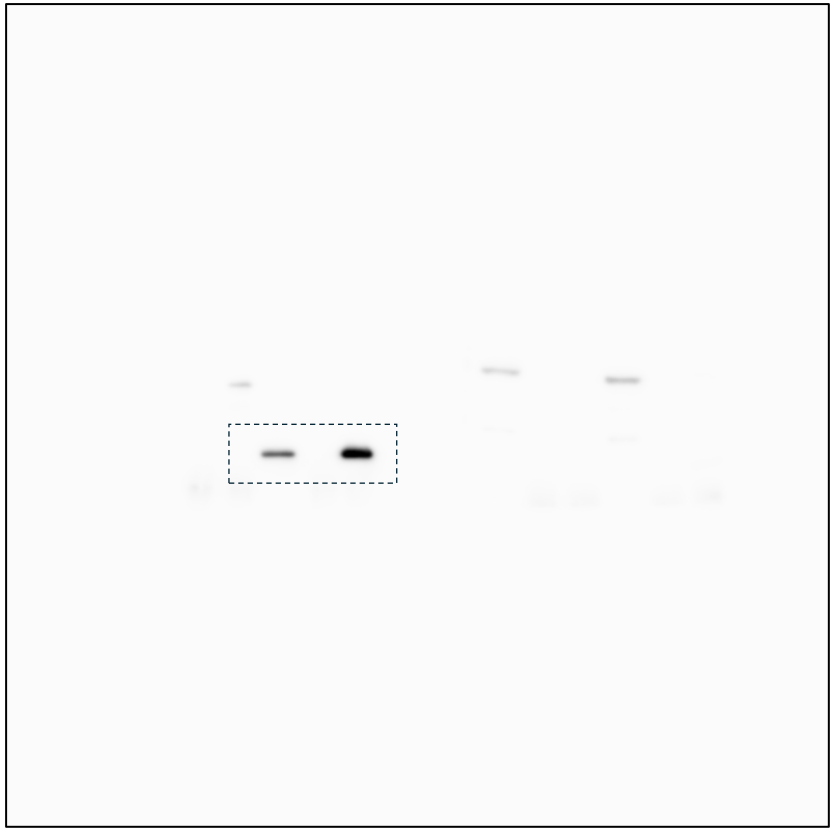

Fig8B WT 1-445 Flag (left),
WT 1-300 SERBP1 (right)

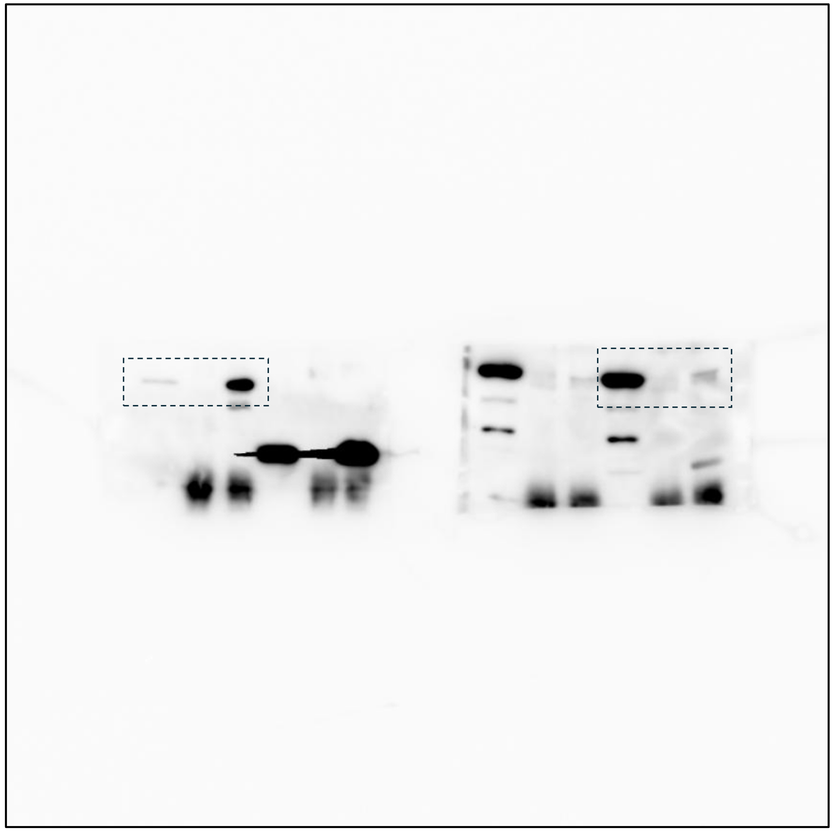

Fig8B WT 1-445 SERBP1

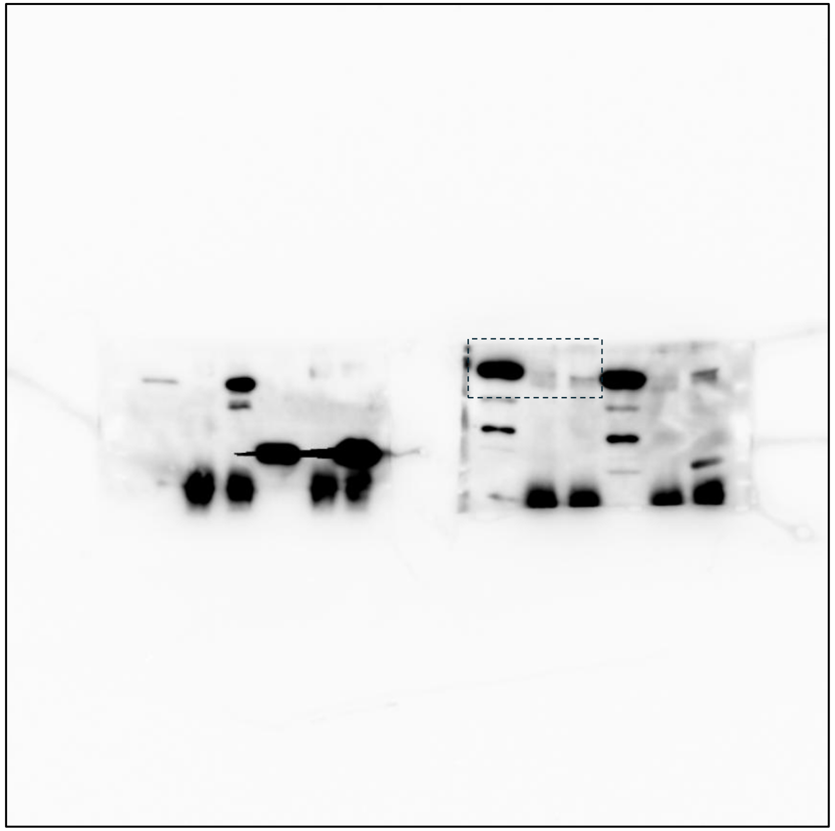

Fig8B WT 301-445 Flag

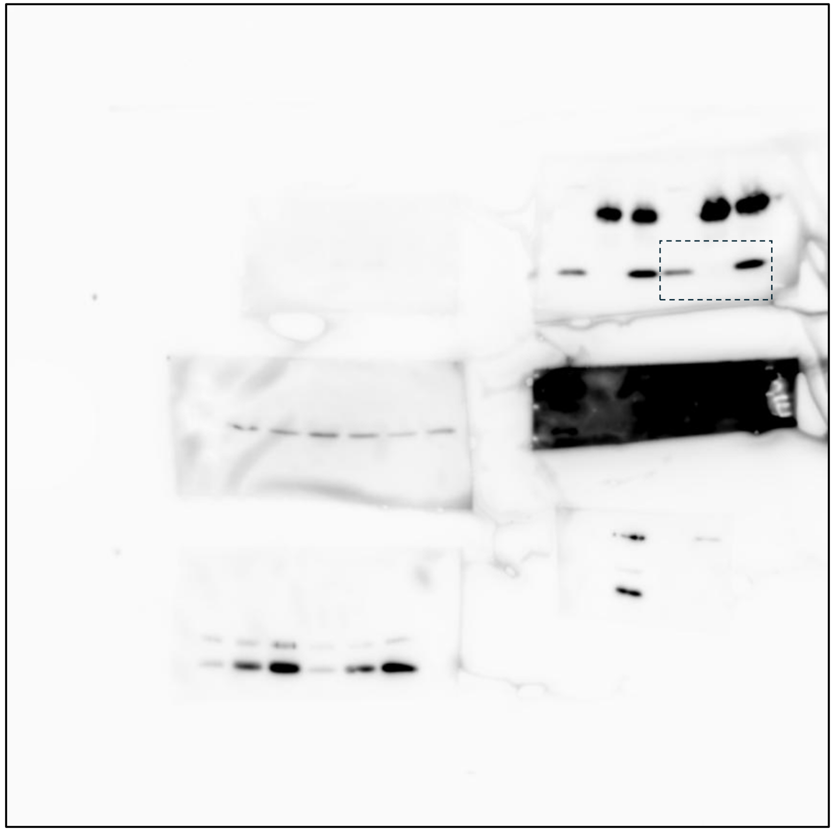

Fig8B WT 301-445 SERBP1

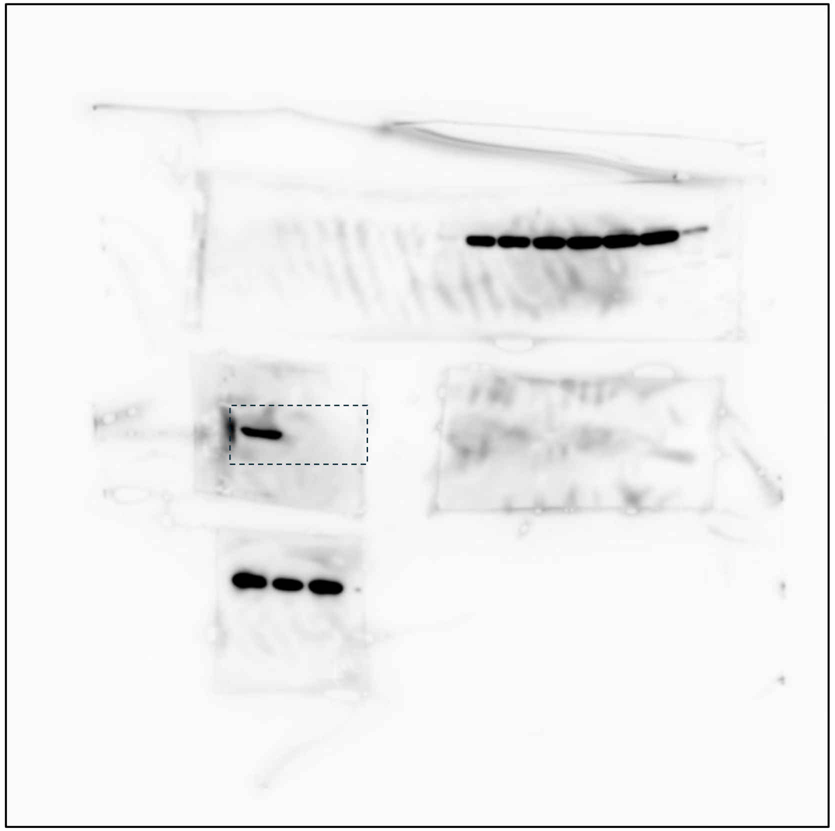

Fig8B K37R 1-445 Flag

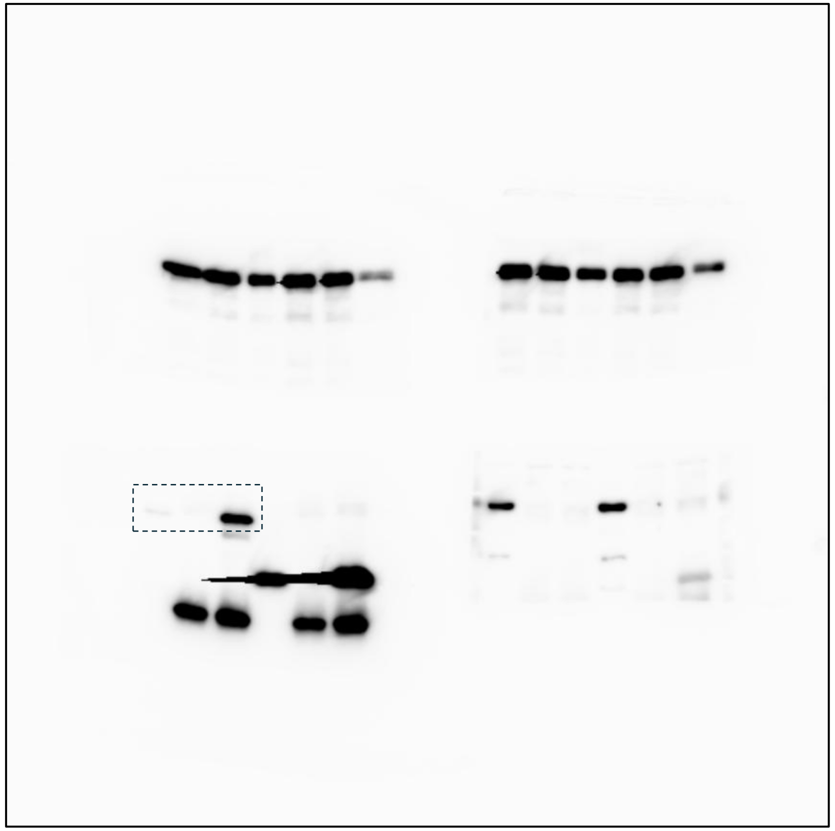

Fig8B K37R 1-445 SERBP1

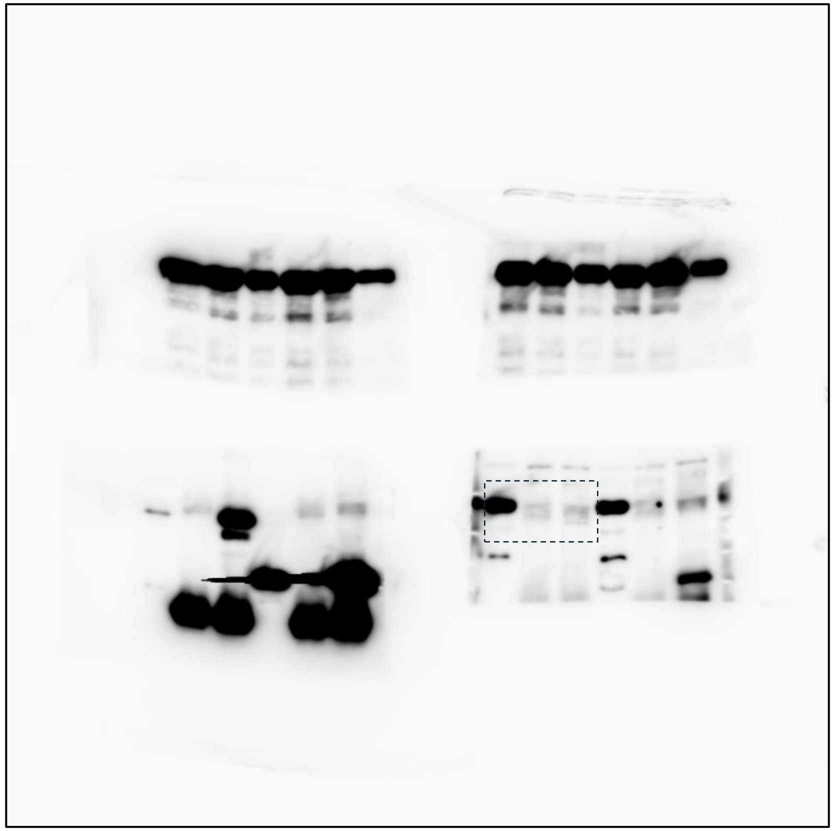

Fig8B K37R 1-300 Flag

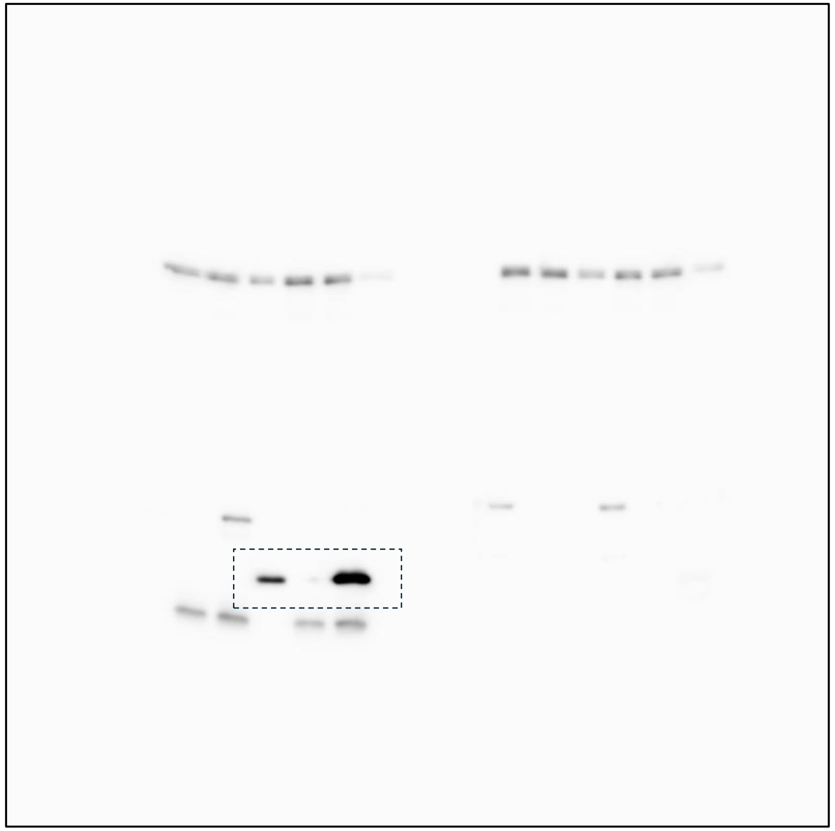

Fig8B K37R 1-300 SERBP1

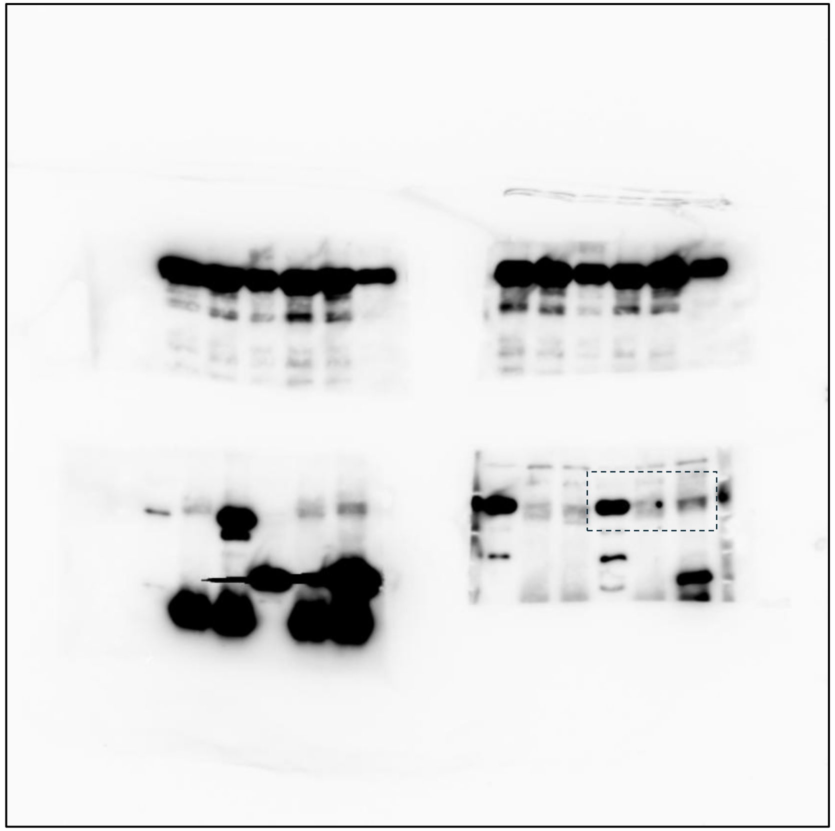

Fig9A PLIN2

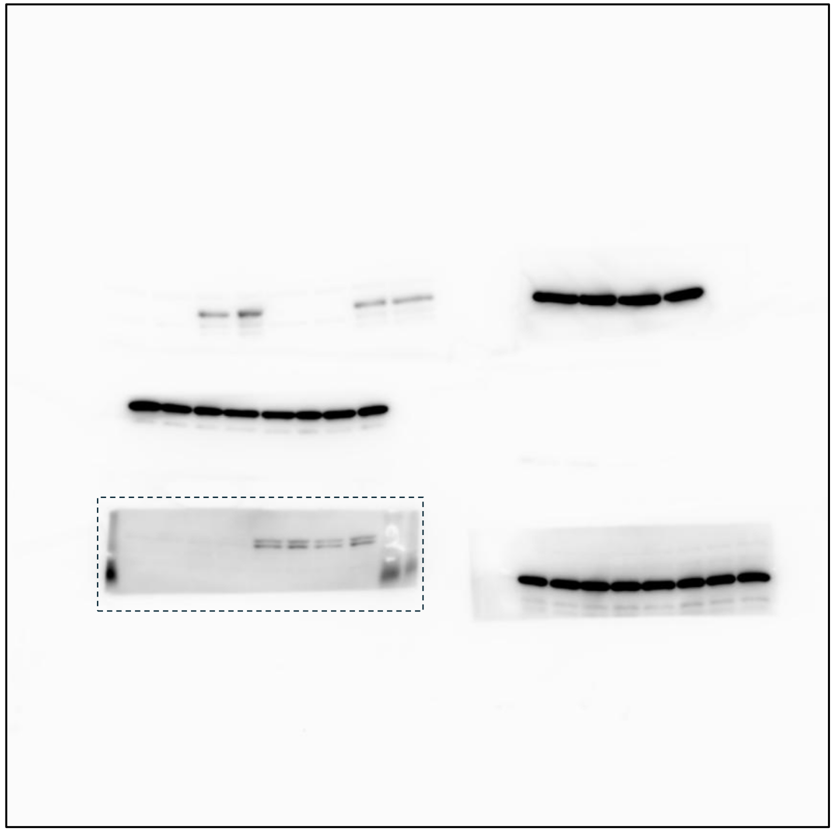

Fig9A GAPDH (left), PLIN3 (right)

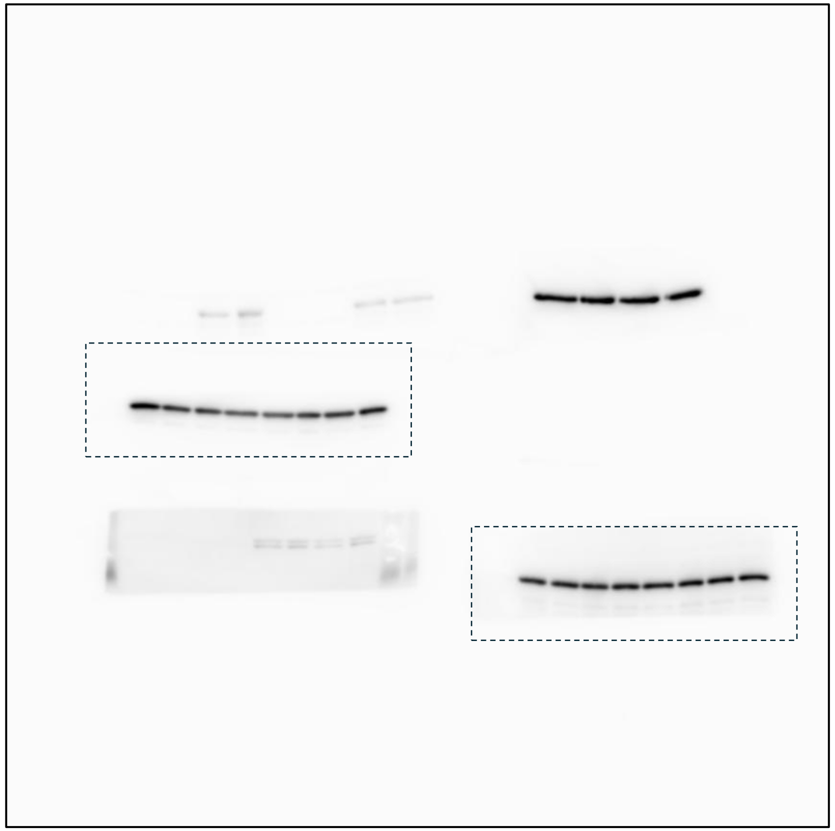

Fig9A NEK2

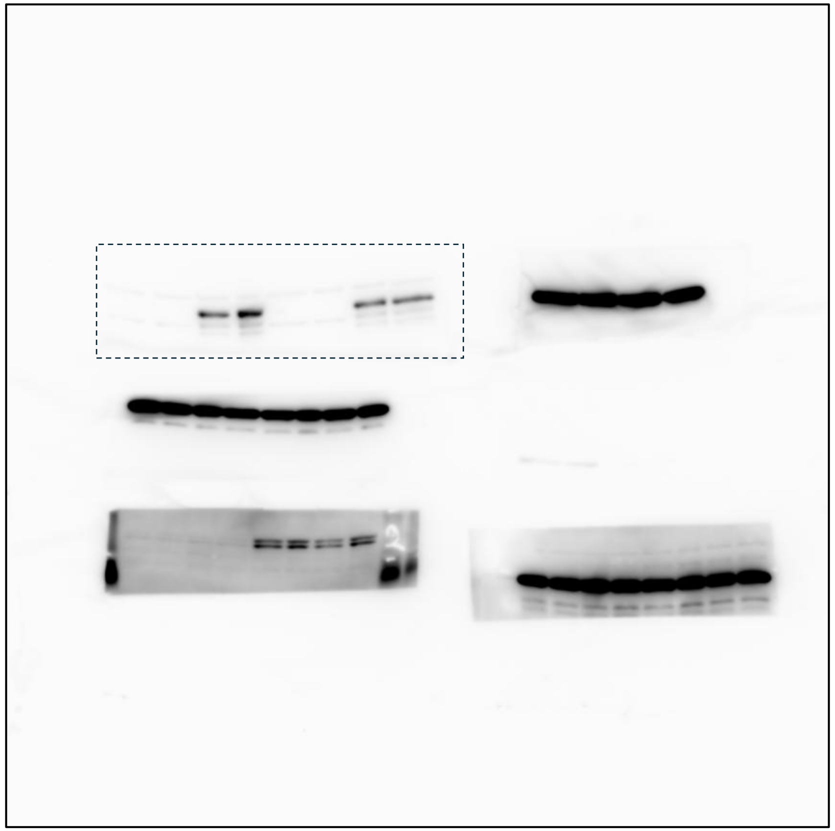

Fig9A SERBP1

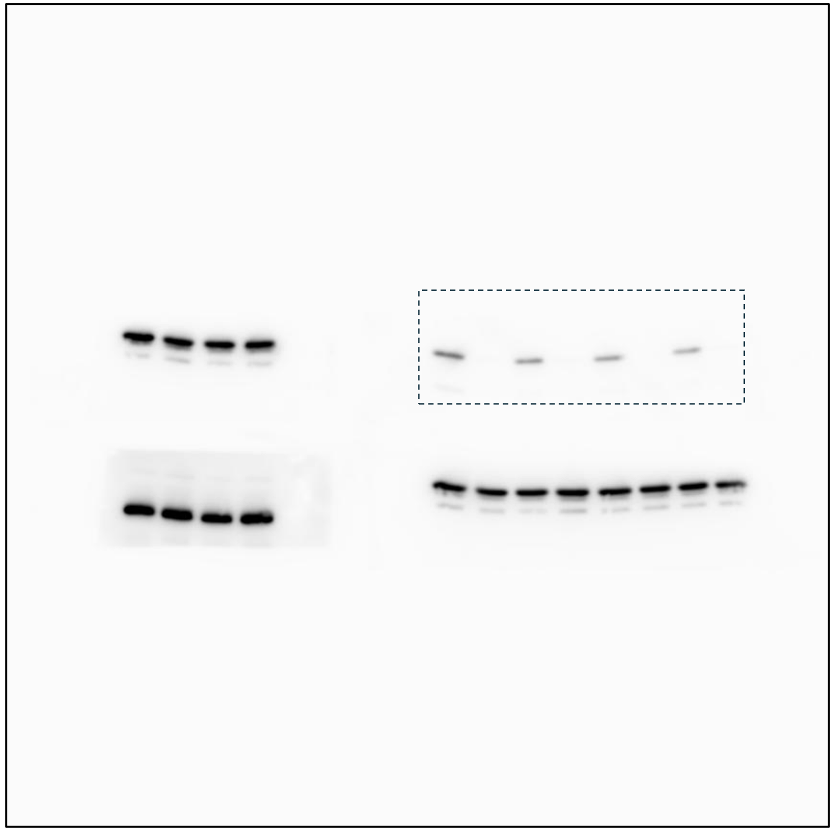

Fig12A GAPDH

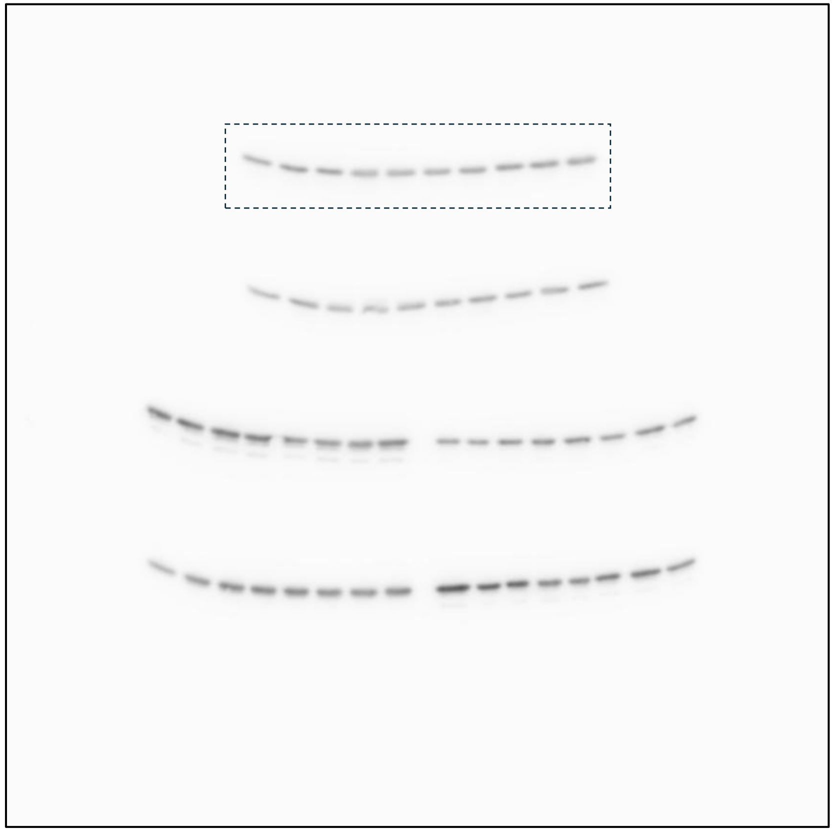

Fig12A PLIN2

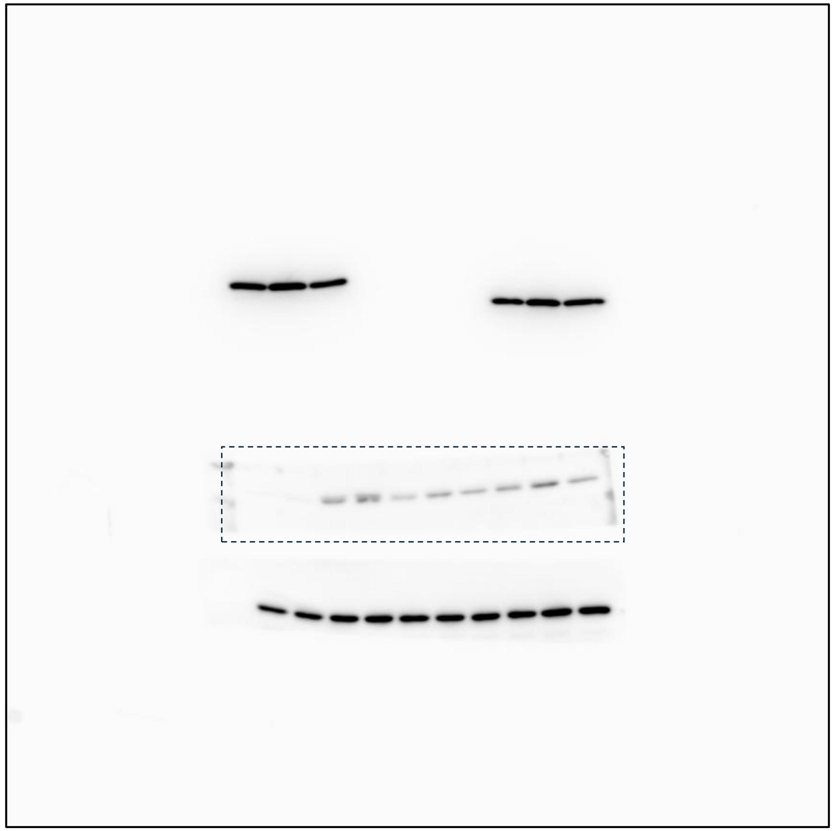

Fig12A PLIN3

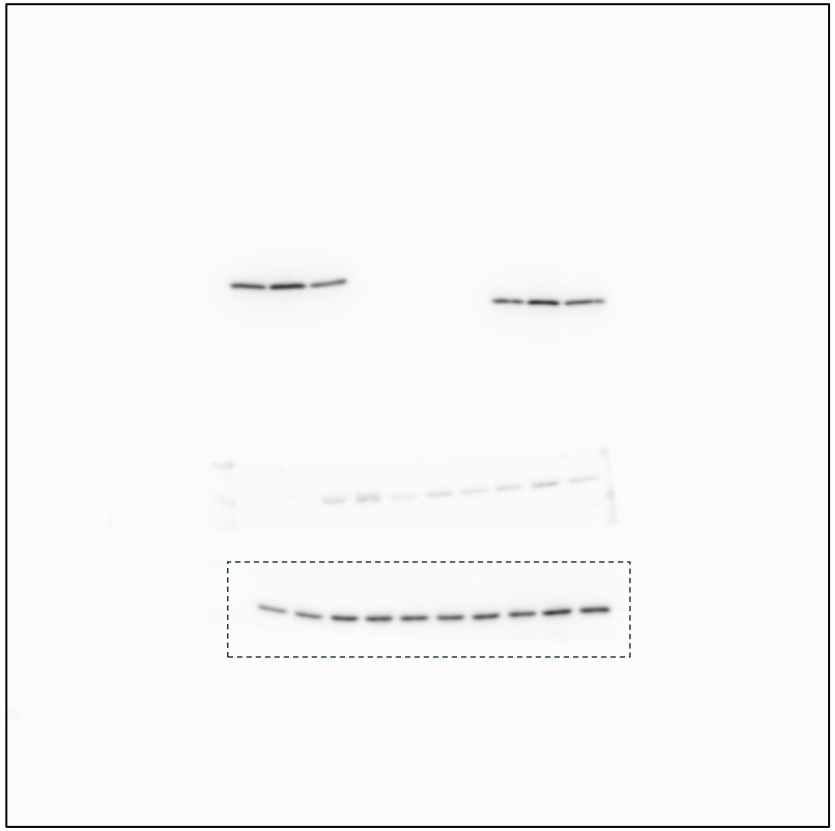

Fig12A SERBP1

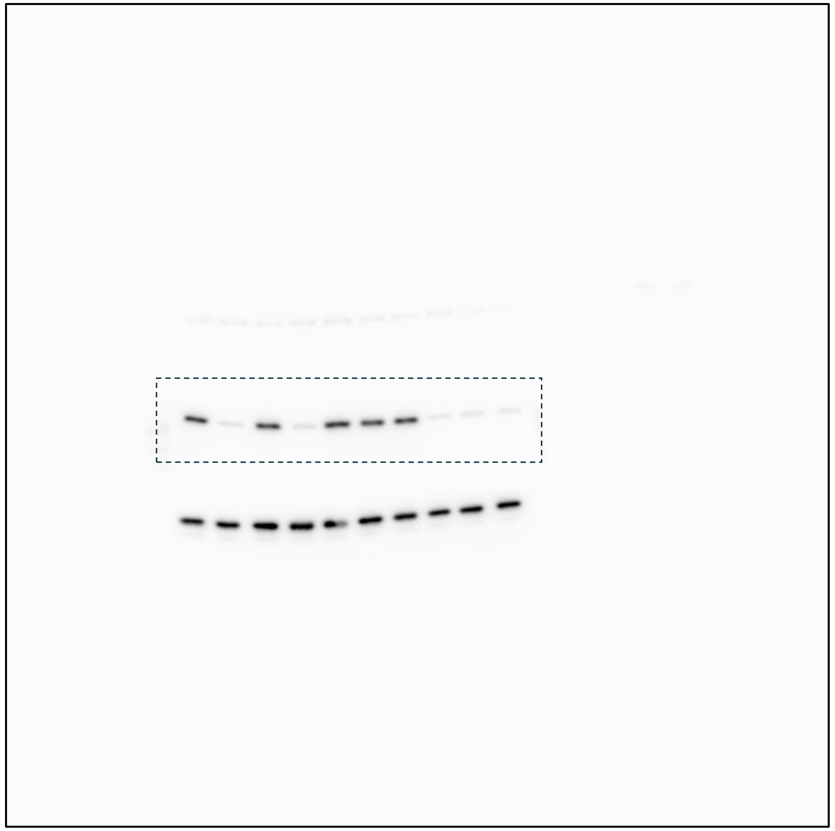

FigS1 PLIN2

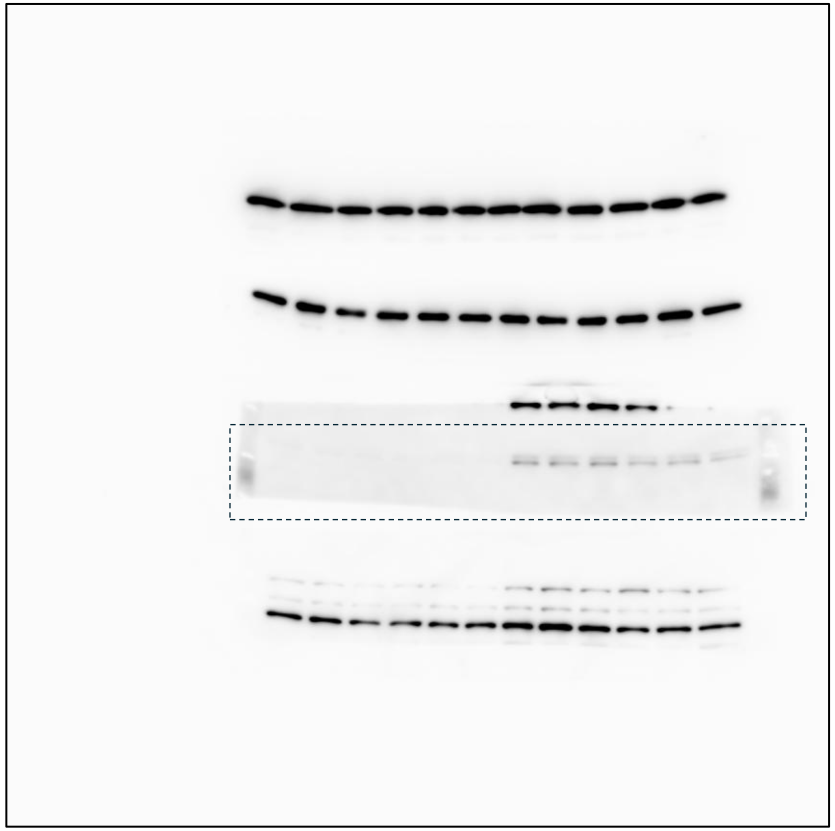

FigS1 PLIN3 (upper), GAPDH (lower)

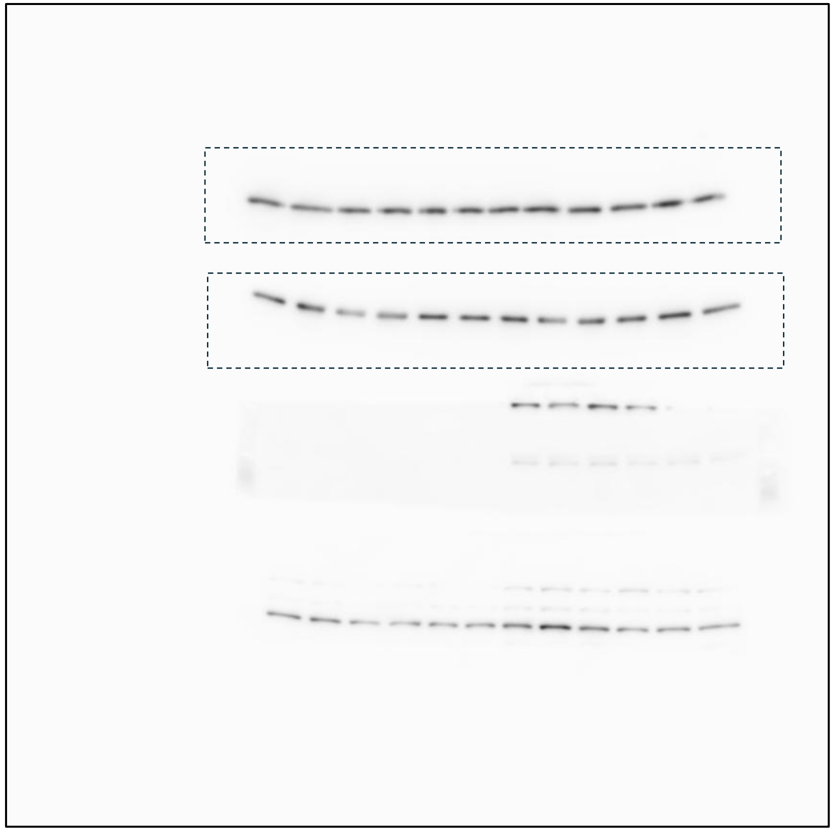

FigS1 NEK2

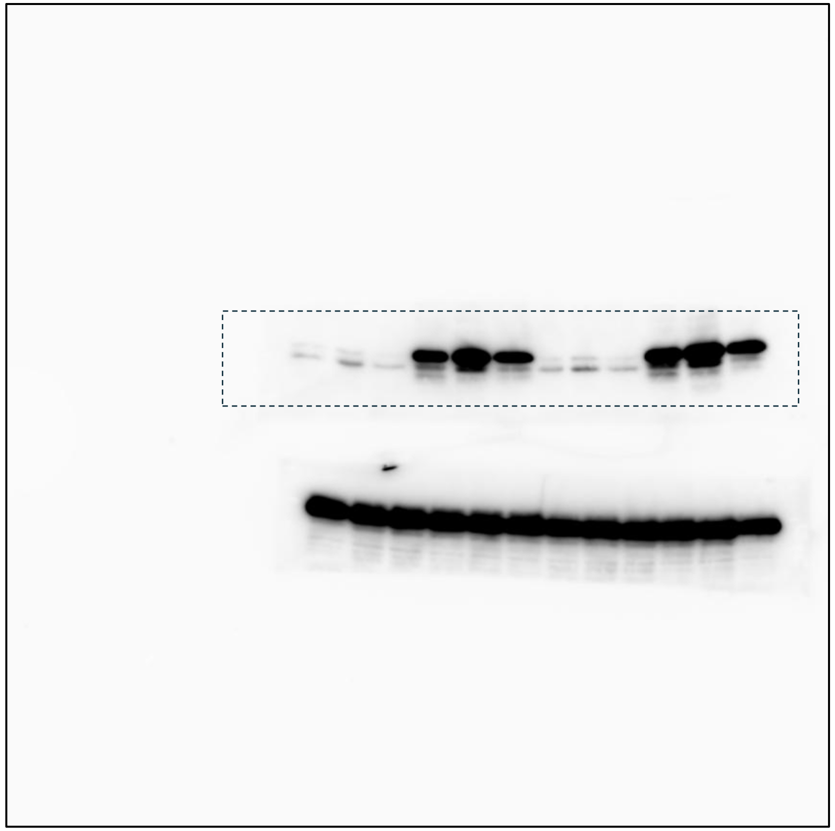

FigS1 LC3

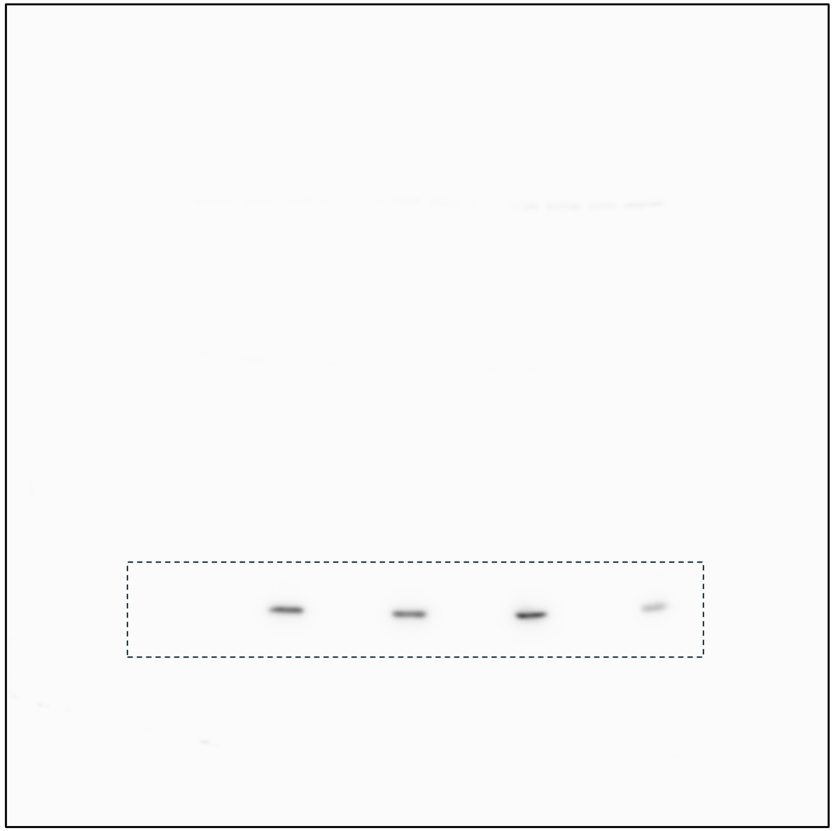

FigS5 GAPDH (upper), PLIN3 (lower)

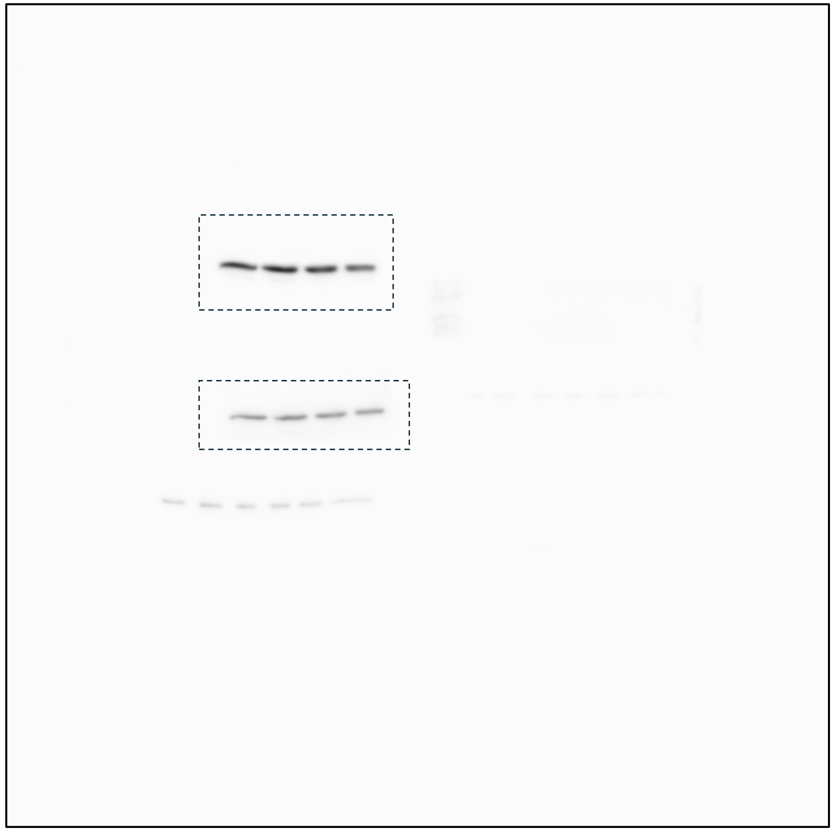

FigS5 NEK2A

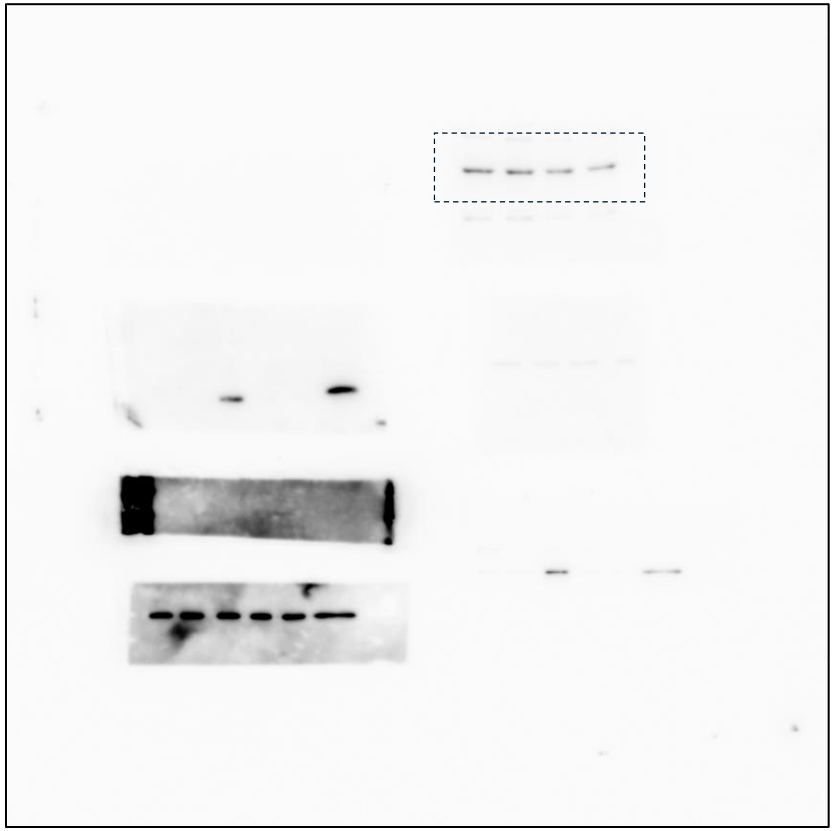

FigS5 PLIN2

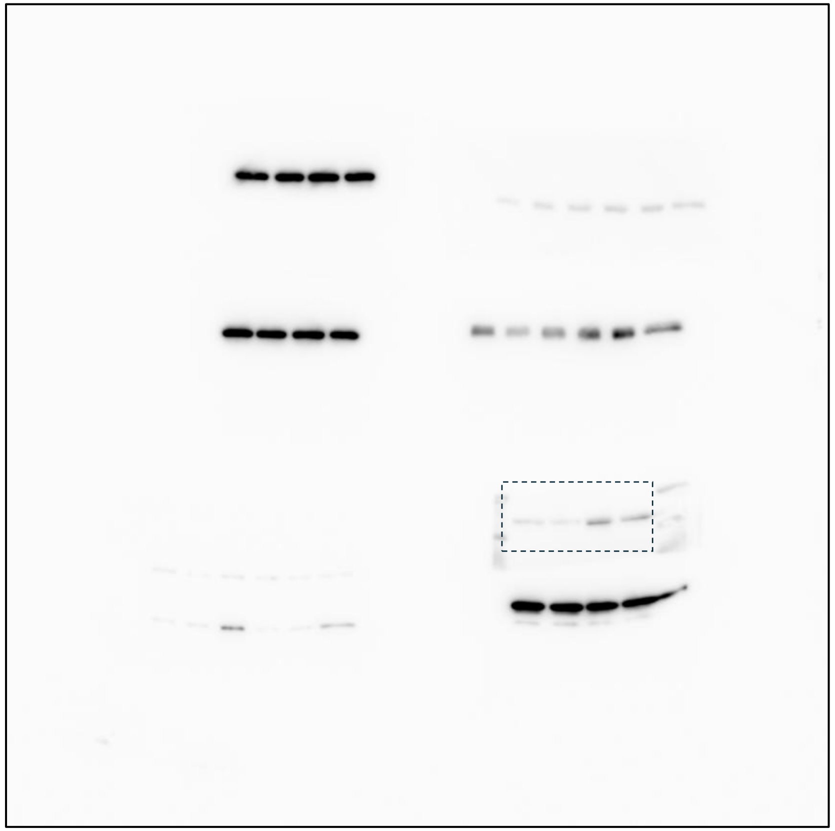

FigS6 Flag

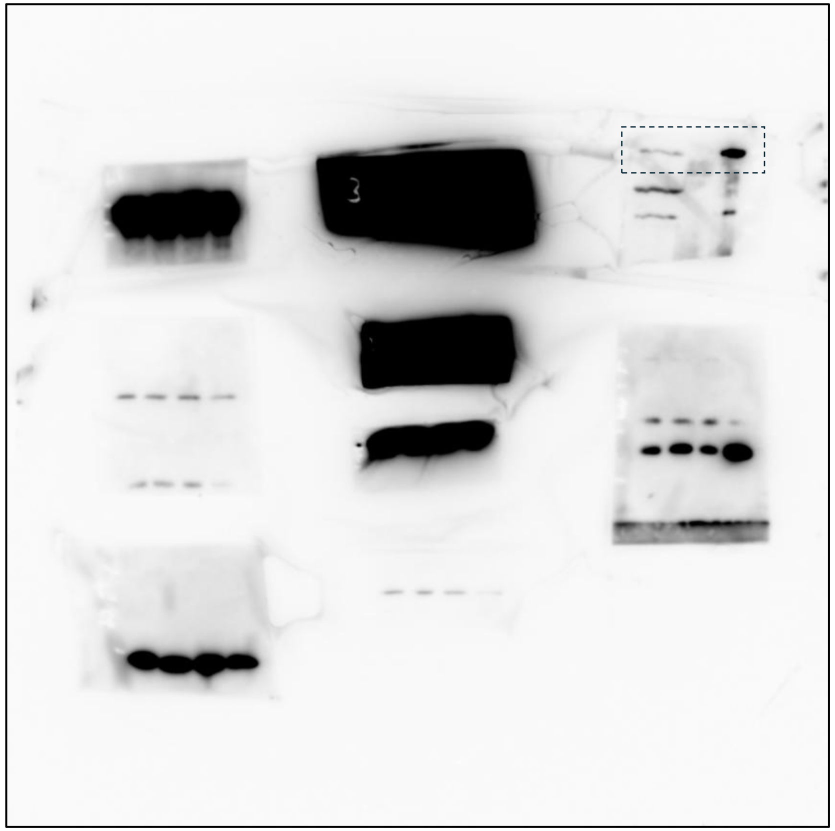

FigS7 GAPDH

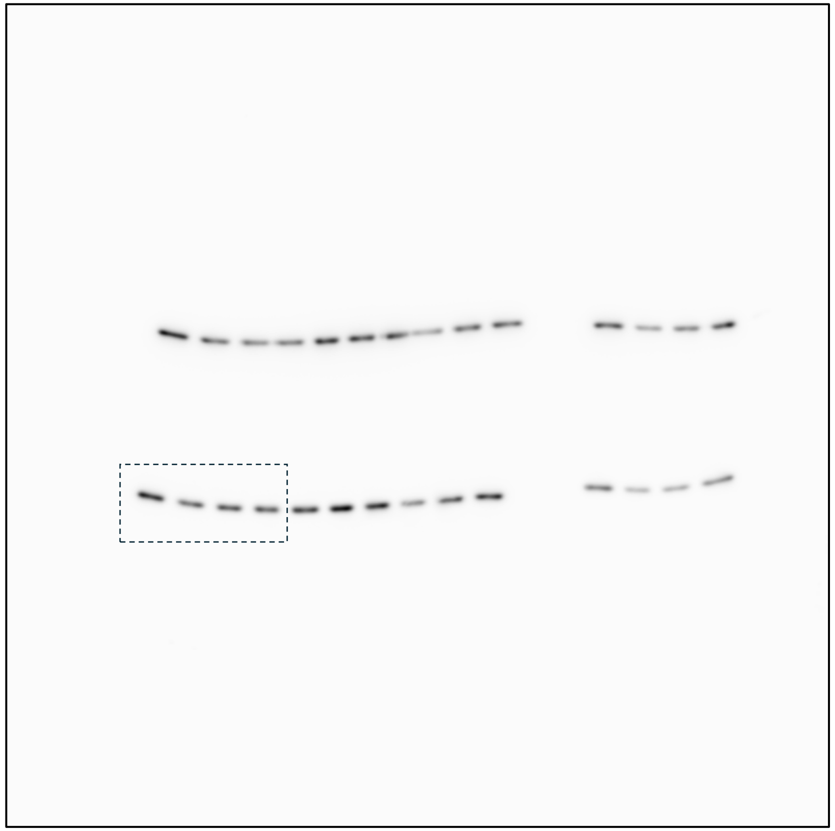

FigS7 PLIN2

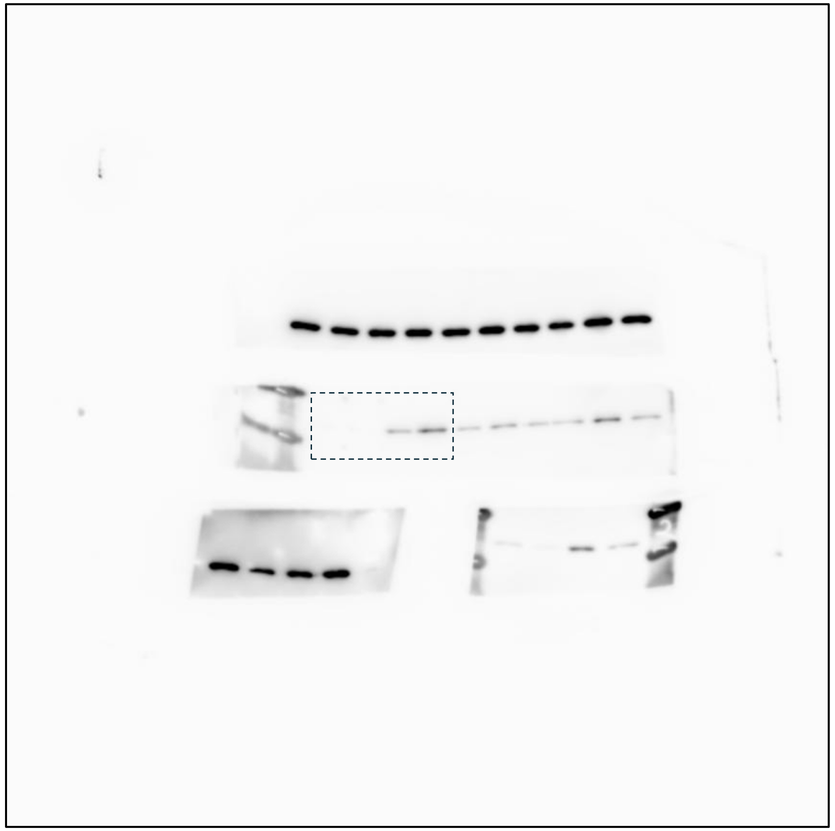

FigS7 PLIN3

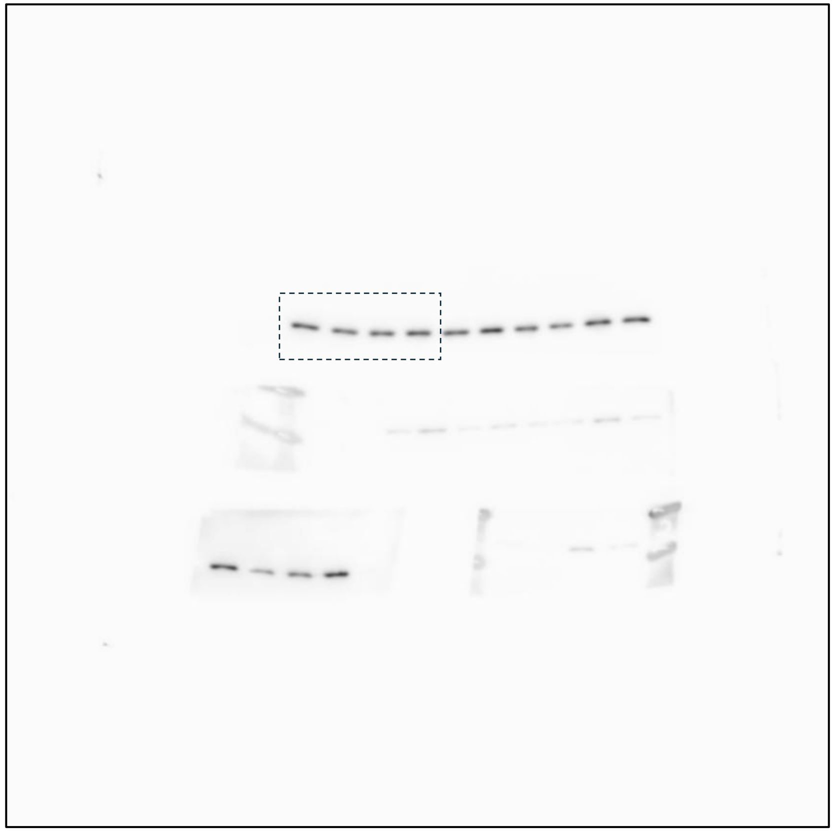

FigS7 SERBP1

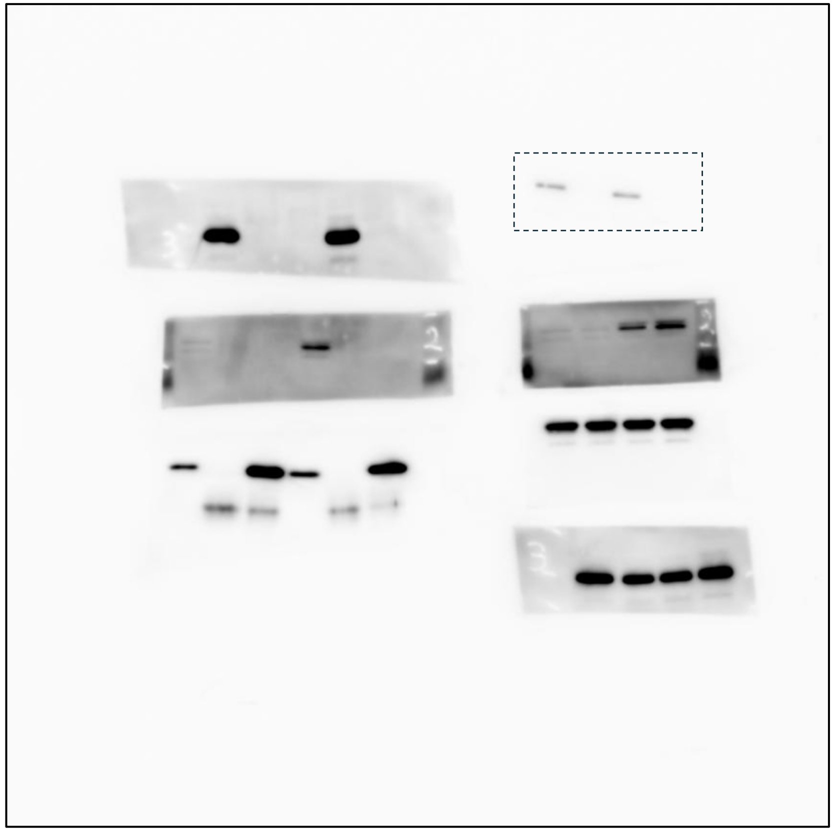

FigS7 PPARγ

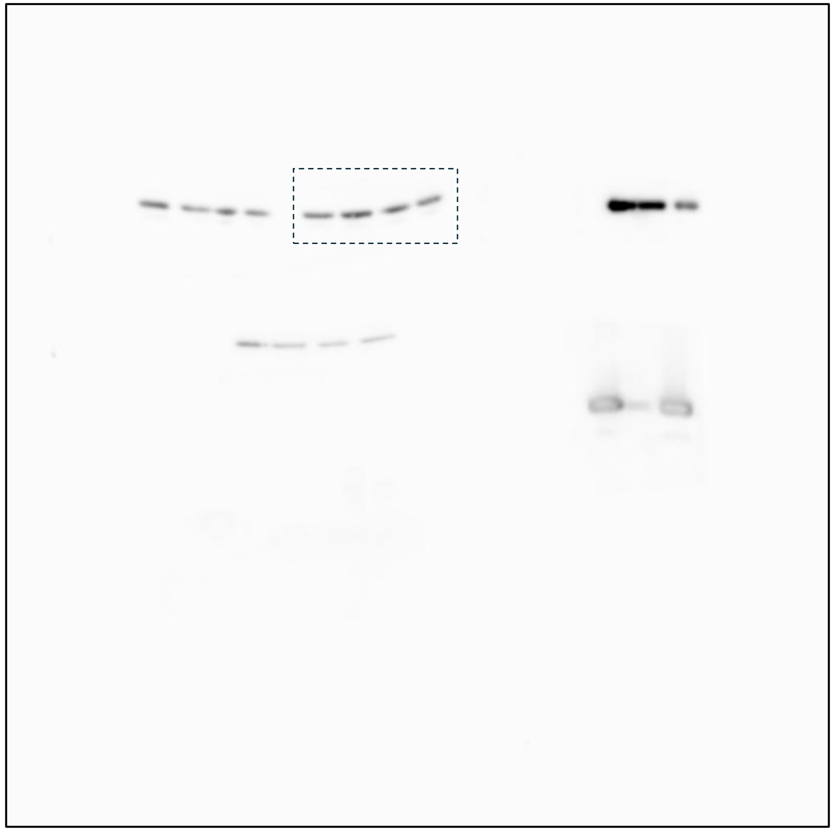

FigS8 - SERBP1

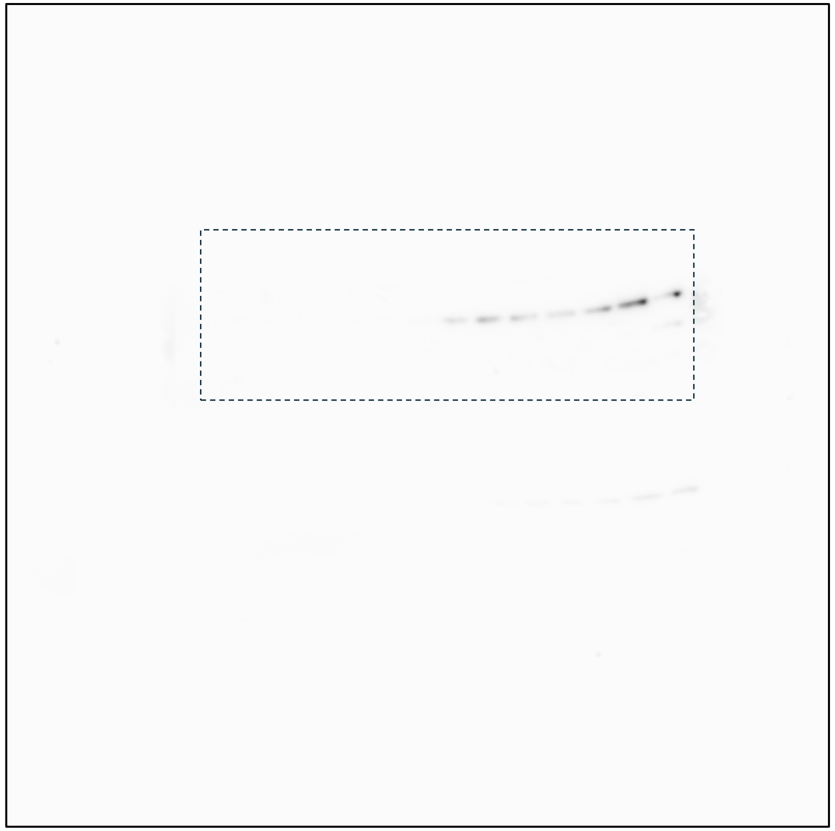

FigS8 WT SERBP1

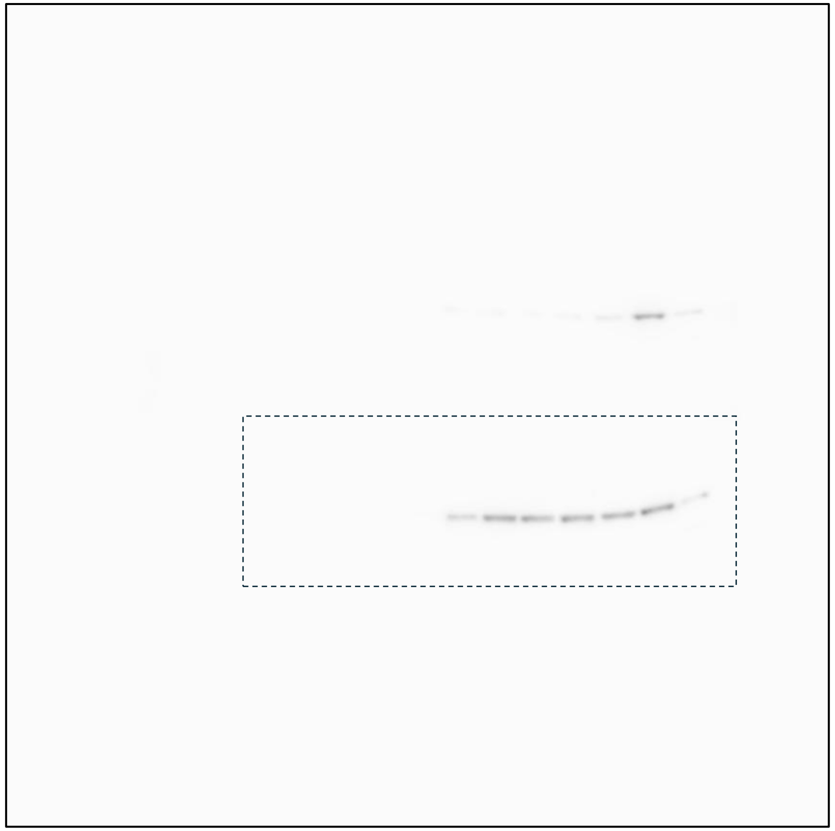

Supplement: Document S1. Figures S1–S8, Tables S1–S4, Data S1 [file mmc1.pdf]
